# A database of global marine commercial, small-scale, illegal and unreported fisheries catch 1950–2014

**DOI:** 10.1038/sdata.2017.39

**Published:** 2017-04-11

**Authors:** Reg A. Watson

**Affiliations:** 1 Institute for Marine and Antarctic Studies, University of Tasmania, Private Bag 49, Hobart, Tasmania 7001, Australia

**Keywords:** Environmental chemistry, Ecosystem services, Marine biology, Fisheries

## Abstract

Global fisheries landings data from a range of public sources was harmonised and mapped to 30-min spatial cells based on the distribution of the reported taxa and the fishing fleets involved. This data was extended to include the associated fishing gear used, as well as estimates of illegal, unregulated and unreported catch (IUU) and discards at sea. Expressed as catch rates, these results also separated small-scale fisheries from other fishing operations. The dataset covers 1950 to 2014 inclusive. Mapped catch allows study of the impacts of fisheries on habitats and fauna, on overlap with the diets of marine birds and mammals, and on the related use of fuels and release of greenhouse gases. The fine-scale spatial data can be aggregated to the exclusive economic zone claims of countries and will allow study of the value of landed marine products to their economies and food security, and to those of their trading partners.

## Background & Summary

Fishing operations span the globe and occur in all but the deepest and most remote places in global oceans^1,2^. Fishing remains central to the food security of many countries. It provides much needed protein and income to those with few alternatives^3^. To wealthier nations it is associated with an extremely valuable and a highly globalised seafood trade^4,5^. The world’s oceans hold continued promise to provide a range of vital services, and fishing will remain important. Conflict for coastal land use, pollution^6^ and other increasing population-based demands^7^ are compounded by ocean acidification, warming^8,9^, spread of pests, deoxygenation and toxic algae blooms. Humans need to guard marine resources, and mapping global fisheries is an important element. Fishing effort continues to increase, putting pressure on marine resources^10,11^. Large ocean areas have been set aside from fishing as marine protected areas but placing these also requires knowledge of fishing patterns^12^. Knowing the details of global fishing operations remains an important part of ensuring that the ocean’s services and productivity are not misused^13,14^.

Examining the relationship between global fisheries and the marine environment, including its wildlife^15^ and sensitive habitats^16^ is challenging but is necessary before the impact on biodiversity and its values can be estimated^17^. Publically available fisheries records are vague, especially in locating where fishing occurs. Nevertheless, it is vital to map fishing and use all available information to do so. This information includes all public sources covering various spatial scales, and auxiliary data such as the distribution of the reported taxa, and information on the distribution of fishing fleets based on access rights and on their observed behaviour. Datasets have been compiled with increasing skill since 1999 and are renewed as more data become available. The approach here is to use a harmonised global dataset from the best public sources, interpolate missing taxonomic data, then map the records to a grid of 30-min spatial cells so as to remain consistent with all available auxiliary data and to make that dataset publically available (Data Citation 1).

**Figure 1:**
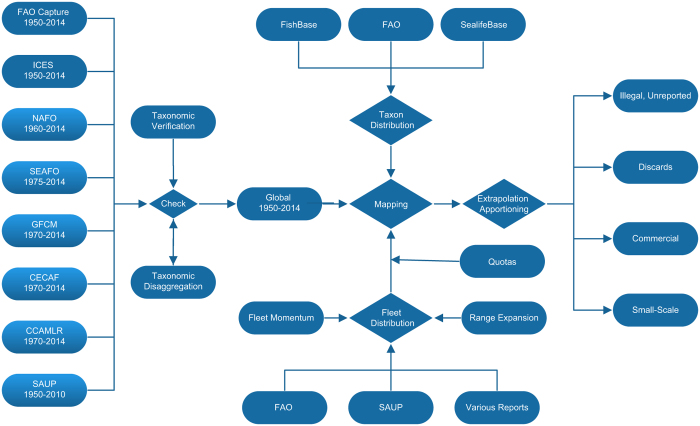


Data was sourced from a range of public sources (Fig. 1). These were harmonised into a single global dataset with common coding. For each location and year, the best coverage from the available sources was selected and overlapping data removed. This dataset was filtered to retain only marine animals but excludes amphibians, reptiles, birds and mammals. The records were mapped to candidate cells within a system of nearly 300 k global 30-min spatial cells using information on the reported fished taxon’s distribution, the behaviour and access of the reported fishing fleets and any area description provided. A portion of the reported landings represented by each record of the unmapped global dataset was mapped to each candidate cell following a gradient based on the reported taxon’s expected distribution based on depth, habitat and other requirements. Known quotas imposed on fishing fleets were applied.

The result was a mapped dataset of catch rates (tonnes per square km of ocean) for each spatial cell separated by year, fishing nation and fished taxa. This data set was further extended to breakdown the reported landings by fishing gear type based on associations with year/country/taxa. Following this, the catch rate of illegal and unreported landings was estimated for each data record. An estimate of discards (not necessarily of the reported taxa) is also made. Though much of the input landings would be derived from large-scale fishing operations it was possible to estimate rates from small-scale fishing and adjust catch rates to minimise duplicate reporting.

**Figure 2:**
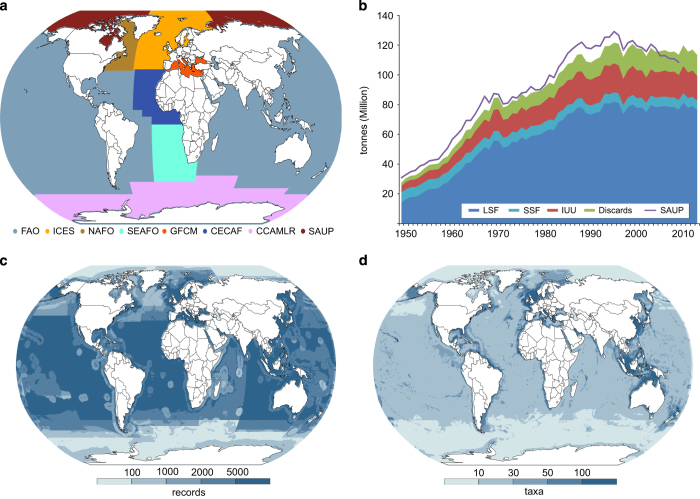
Breakdown of database contents. (**a**) Map of database source coverage used (see Table 1); (**b**) Total tonnage and breakdown from 1950 to 2014, LSF is large-scale fishing, SSF is small-scale fishing, IUU is illegal and unreported fishing, Discards are rates of discard at sea and SAUP provides a comparison with the global total for on-line country catch reconstructions by SAUP^18^; (**c**) Map of number of database records; (**d**) Number of reported taxa.

Non-overlapping data sources are selected as input (Fig. 2a). In general, the United Nation’s Food and Agriculture’s (FAO) dataset was the only source that provides global coverage but spatial resolution can be quite coarse. FAO’s various regional bodies provide finer spatial definition for several areas and those were used when available and possible. The breakdown of global tonnage represented by records (Fig. 2b) correlates generally to those developed by reconstructions of individual countries in another global dataset (SAUP)^18^, which uses different methodology. The number of database records varies spatially (Fig. 2c) and was impacted by the diversity and intensity of fishing and the level of management control. The number of different taxa reported also varies and is greater in coastal areas (Fig. 2d).

## Methods

### Data sources

Input data were collected via the Internet from a range of public sources (Table 1). Each covers a range of years, however, until recently, only capture landings data from FAO provided global coverage. Data sources used here are accessible via the Internet and allow use of their products with suitable requested acknowledgement. Typically the data is released under a create commons licence in which the user agrees to acknowledge the data sources. Governments, which have the obligation to collect and provide their fishery statistics to the international organizations responsible to manage these fishery resources, are required to maintain costly infrastructure to collect and analyse such data. It is common for government bodies with each country to gather statistics, which are provided directly to the agencies sourced here or through in-country expert consultants. Ultimately the provision of the data and the support of international and regional bodies is taxpayer funded but essential. Acknowledgement of original data sources is suggested, however, even these sources are compilations of national and regional datasets with many unnamed, dedicated experts. These national bodies have in turn received much of their information from other groups within their respective jurisdictions including fishing companies and their respective organisations. In many cases the initial reporting may have originated on a compulsory and voluntary basis from individual fishers and their respective logbooks. Thus every year global fisheries statistics are produced there may conservatively be thousands of people involved most of which will receive no recognition.

For many aspects of fishing, for any jurisdiction, there is often a choice of data sources. Some like the FAO’s dataset are global landing compilations from national contributions; others like the Regional Fisheries Management Organizations (RFMO) can be more detailed including catch and effort distribution focused on one particular area and vary in their coverage of fished taxa (e.g., ICES, NAFO and some tuna RFMOs). There is, however, no harmonization between many of these more detailed statistics, and thus when not available or not possible due to time or confidentiality constraints, FAO statistics were used for these areas or species as this source typically includes all the landings that RFMOs’ provide. In the case of tuna RFMO data, a decision was taken to use FAO’s tuna statistics, to which tuna RFMOs contribute, but to use the information available from the Atlas of Tuna and Billfish which display tuna and billfishes global catches by gear by 5×5 degree resolution (http://www.fao.org/figis/geoserver/tunaatlas/) to provide information on annual fishing patterns for tuna and billfish. This decision was taken because the tuna RFMO data did not cover all landings by non-industrial means, and typically had missing years in their time series. There were often considerable lags until all bodies provided annual information. This decision will be reviewed before each new data version is produced and efforts to include the more detailed tuna RFMO publically available data will be attempted in future updates.

All of these sourced data sets had to be converted to flat files from a variety of formats, and provided with common coding for fishing country, fished taxa and reporting areas.

Each global area was represented in any year by the best-input data, and selection was based on the completeness of reporting (details of fished taxa) and the spatial precision provided in the statistical areas used. For example, the ICES source for the northeast Atlantic had better spatial definition with its small statistical areas than did the FAO data for the same ocean area so the former was used. Typically regional bodies provided a finer breakdown than sources covering global fishing. As Fig. 2a indicates, much of the world’s oceans are covered by FAO official reporting only, but it was possible to replace whole ocean areas by more informative data sources in several cases. The harmonized global dataset subsequently mapped used the sources identified in Table 1.

### Taxa excluded

Only taxa with a marine origin were used—though some species occur in marine, estuarine and freshwater environments. Aquaculture production was not used from the sources, only wild capture was included and mapped. Records of shells, coral and similar were not used. Reports of amphibian, reptile, bird or mammal captures were also not used.

### Correction to reported taxa

In situations where the reported taxon was highly aggregated and described in ways like ‘marine fishes’, the record was disaggregated to the compatible breakdown of neighbouring countries with better detail (taxonomic disaggregation). The range of animals reported was checked with known distribution limits^19^ and obvious misidentifications were corrected (taxonomic verification). Corrections sometimes required adjusting the taxonomic description to a more general level, for example, from the species to the family level.

### Correction to fishing country

Unfortunately there is, as yet, little information to guide attempts to reverse the reflagging of fishing vessels. Sometimes when a country that reflags many vessels reports catch which originated in areas that their fleets do not fish and in fact do not have access rights to, it was possible to consult a list of the likely original flag nation and attempt to map the catch record with that nationality. Thus far this can only be attempted with reflagged European vessel based on a single reference^20^. This is still a relatively crude and unsatisfactory process while records of reflagging are kept confidential and appropriate databases to guide the process are very limited.

Where possible reflagged fleets are corrected to their likely true identity as this relates to access rights assumed in the mapping process. As most marine resources are in the shallower inshore areas now claimed by coastal countries as their Exclusive Economic Zones (EEZ), the fishing access of fleets is an important part of mapping global fishing and is a major part of the resource management^21^.

### Auxiliary data

Auxiliary data is a term used to describe other factors known about the landings other than the actual weights reported. These include the taxonomic identity of the reported catch. This is usually provided by data sources as the common English name for one or more species. This in turn provides information about the distribution of the taxon and its preferred and required habits. This is essential information for the subsequent mapping process. Distributions of most landed taxa are known and described in a number of useful sources^19,22^ including Fishbase http://www.fishbase.org. These distributions are usually associated with marine habitats that can be described by such factors as the presence of macroalgae, coral reefs, seagrasses, seamounts, distance from shore and many others.

There are published associations between the catch of marine taxa by countries by year and the fishing gears used^16,23^. The literature and internet were used to create a database that associates up to 5 different types of fishing gear with the capture of each taxon by country and year. Extrapolations were made to provide associations with all catch database records^16,23^ and these were used to prorate the mapped landings into catches by each gear type.

Fishing fleets also have known behaviours^24^. Some do not leave their national waters, others travel to the waters of other countries to fish, while some fish on the high seas. Databases of fishing arrangements^25^, researched through trade papers and journals, can suggest where fleets access specific taxa in the EEZ claims of other countries.

### Corrections for fishing fleet range

It was important to correct for the offshore limits to fishing fleet operations when assigning catch to spatial cells. It is accepted that the range of fishing fleets have evolved over time based on changes to technology and also the general depletion of stocks closes to harbours and shores. A documented expansion for fishing has been observed and operates in a ratchet-like way generally related to the development of the fishery as characterised by the annual trajectory of reporting landings^2^. This correction was applied independently to each taxa/country combination within each of FAO’s large ocean-scale statistical reporting areas. Typically the assumption used is that when a fishery for a taxon reaches its historical peak in an ocean area, the fleet’s fishing range eclipses the entire distributional range of that taxon. That is the fleet’s fishing range and the taxon’s distributional range overlap. No subsequent contraction of fishing range is used.

### Mapping

The harmonised and verified global dataset of reported landings (1950–2014) prepared as described above was mapped. The spatial disaggregation process chose spatial cells that hosted the reported taxa within the nominated reporting area but were also accessible by fishing fleets of the reporting nation for those taxa in that year. The latter condition is based on known fishing arrangements^25^ or derived fishing fleet behaviour (historical observations). Within this collection of candidate spatial fished cells, the reported tonnes were distributed using the gradient of the reported taxa’s habitat requirements and the area of ocean in each cell^24^. Note that 30-min spatial cells are largest at the equator and decrease to the poles, and might lie only partially on ocean areas. The results were catch rates (tonnes per square km of ocean) for each spatial cell for each fishing nation and fished taxa available annually from 1950 to 2014.

### Association with fishing gears

The mapped catch rates were then associated with fishing gears based on reports in the literature and on-line. Associations depend on the fished taxon, the fishing country and the year it was fished^16,23^. This process increases the number of database records as it prorates each record into a number of records for each associated gear.

### Illegal, unreported, and unreported catch and discard estimates

It is important to recognise that not all catch that is landed is reported and would appear in the pubic record, and that many marine animals are collected and subsequently discarded (not landed) and may also not be documented. The reported landings dataset was extended to produce a breakdown of illegal, unreported and discard catch rates by fishing gear type based on ranked associations with year/country/taxa^23^. Following this, for each record, an estimate of the catch rate of associated IUU was calculated^26^ as well as an estimate of discards^27^. IUU were retained catches which for whatever reason were not included in the public record. Discards are those catches which are not retained or landed. Discard catch rates that were estimated were not expected to be the same taxon as the reported landed taxon for each record, but rather would be comprised of a variety of taxa, targeted and non-targeted, many of which would not be represented in the original landings source data.

### Small-scale fisheries

In addition, for each record, there was an estimate of small-scale fishing provided^28^, with the original landing estimate adjusted when reporting may overlap. The portion that was not deemed small-scale is referred to as large-scale (commercial) catch rates. Small-scale fisheries were deemed to occur only in spatial cells that were both within 200 km of shore and with depths of 50 m or less.

The reported small-scale catch rates for each country, based on its development status^28^ were prorated amongst candidate mapped records. An assumption was made that the degree to which small-scale fishing was fully represented in the original data sources was proportional to the national corruption index, that is, countries with high levels of corruption^29^ are unlikely to forward full and complete records to the agencies compiling fisheries statistics and small-scale fisheries would be under-reported. When some or all of the small-scale fishing was deemed as already represented in the landings provided by data sources then the ‘large-scale’ catch rate was prorated accordingly to avoid overlap.

### Overview

The process overall is one where public sources of reported fisheries landings are harmonised to present the best global dataset which is then mapped to spatial cells based on taxonomic and fisheries logistic considerations. This is associated with fishing gears. Following this, IUU and discards are added. Small-scale operations are added and adjustments made for overlaps.

### Code availability

The programmed procedures used to process data were written in Microsoft’s VB.net and employed Microsoft Access and SQL server databases. The distributions of taxa and other supporting auxiliary data are available from a range of sources collated originally by the Sea Around Us project at the University of British Columbia and they can be contacted about availability. Relevant scripts/code used for data assembly and mapping are available as Supplementary Information.

### Future updates

Every year the organisations providing the source data provide updates. Most have a lag of a year or more from current fishing. The current plan is to update the data annually after the release of source updates and to include the best sources of data. Unfortunately it is not possible to know if an agency has altered any of its previous data from it last update. That means that the entire dataset is currently prepared annually though changes to years more than 5-years back or more are unlikely.

## Data Records

Each data record is comprised of 12 descriptive fields, which collectively describe the fishing process associated with the output catch rates (Table 2). These describe the year of fishing, the location by 30-min spatial cell, the fishing country, the reported taxon and the associated fishing gear. For each there is an estimate in catch rate (tonnes per km^−2^) of catch associated with large-scale, small scale, illegal and unreported, and discards. Text fields within each record describe all codes used. The spatial cells have their central latitude, longitude and enclosed ocean area provided. Countries have their UN English name provided. Each reported taxon has both an accepted scientific and common name provided. Fishing gears have descriptive name included. Each record has an estimated catch rate in tonnes km^−2^ yr^−1^ for large-scale fishing, small-scale fishing, illegal (and otherwise unreported) fishing and discards at sea.

## Technical Validation

**Figure 3:**
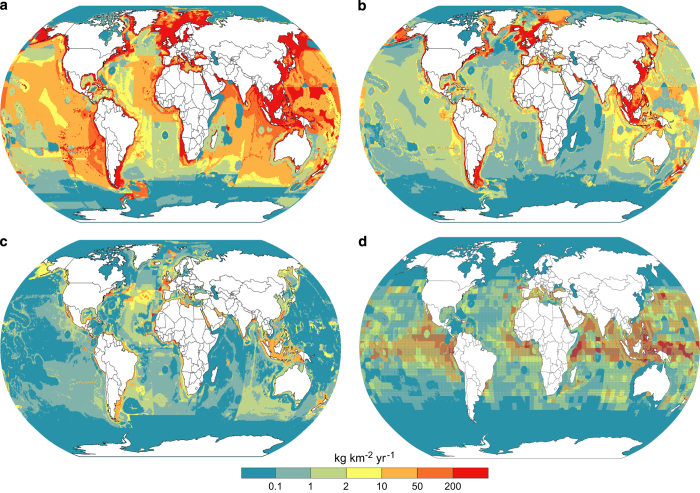
Examples of database use with mapped catch rates (kg km^−2^ yr^−1^). (**a**) Average annual reported catch rates (including IUU) for 2010–2014; (**b**) Average annual catch rate of discarded marine products 2000–2004; (**c**) Average catch rate of sharks and rays 2010–2014; (**d**) Average catch rate of tunas and billfish 2010–2014.

The dataset and its unpublished earlier versions continue to provide a valuable resource for a range of researchers. They are commonly are used to report levels of fishing activity within defined areas such as national EEZ claims or the widely used large marine ecosystems. Some examples are presented here. Figure 3a shows the estimated catch for 2010 to 2014. More productive areas are clearly visible. Similarly it is possible to examine where most discarding at sea is occurring such as for 2000–2004 shown in Fig. 3b. Knowing where fishing is occurring and how the patterns are changing is important to examining possible impacts on vulnerable habitats (coral, seagrass etc.) or on resources used by wildlife like seabirds or marine mammals. The data can help explore where potentially vulnerable groups like sharks and rays (Fig. 3c), or important fisheries like tunas (Fig. 3d) are being taken globally or in any management region.

The data was validated and mapped according to rules that dictate that reported taxa must be available at the location and accessible by the fleets of the reporting country in the year fished by the associated fishing gear. As such the mapping is constrained by many supporting databases that are encyclopaedic in scale (for example FishBase http://www.fishbase.org). Though unlikely to capture details available from tracing ship movements by satellite or even logbooks kept by vessels, it was necessary to use a broad, top-down approach because otherwise a global treatment would not be achievable. Where possible the access of fishing fleets and their fishing behaviour was updated annually (for example with tuna fishing) but often their arrangements are approximated by more static fishing agreements. The distributions of the fished taxa will change with ocean warming and other factors though at broader scales fishing patterns may be slow to change except where thawing icecaps allows new access. Some of the challenges have been discussed elsewhere^14,24^.

## Usage Notes

Most users will find that the complete dataset of nearly 868 million records is quite large to work with. The dataset is available in 5-year blocks starting from 1950. Work with the data has involved either reading flat files or bulk-importing data into a robust database program and ensuring that relevant fields are well indexed. The various descriptive fields will allow useful sub-setting of the data.

Spatial resolution is poorest when the source data had vast statistical reporting areas, when fleets roam globally or when the reported fished taxa are widely distributed. In practice these are not a large problem if the results are used in larger spatial aggregations such as large marine ecosystem but use of data from only a small selection of cells (<100) is discouraged.

As described, the estimate of discarded catch is unlikely to be the taxon reported in that record. That is, if a record describes the catch rate of a shrimp with associated discards then those discards should not be assumed to be that target species but rather a rather large range of poorly valued marine animals best identified by surveys of the area.

The online datasets provided by the Sea Around Us project^18^ are prepared in a different manner but historically had some commonality in basic methodology and supporting databases (such as the ranges of fished taxa). Data here is not reconstructed on a national basis, assigned to national or high seas water areas but rather is leveraged from sources provided directly by reporting agencies (Table 1). Both approaches have their merits. Given the large role that public agency data (such as the UN’s FAO capture fishery dataset) play in providing the ‘backbone’ over which datasets with a more restricted but focused approach are added, it is not surprising that the general patterns of landings in time and space are similar.

The RAM legacy database^30^ provides global insights on global fisheries from the perspective of those fisheries with stock assessments. Assessments tend to be common in developed countries but rare elsewhere. Importantly such databases are essential to make predictions about how exploited stocks are likely to react with future changes, whether due to the environment and through change to the marine environment.

## Additional Information

**How to cite this article:** Watson, R. A. A database of global marine commercial, small-scale, illegal and unreported fisheries catch 1950–2014. *Sci. Data* 4:170039 doi: 10.1038/sdata.2017.39 (2017).

**Publisher’s note:** Springer Nature remains neutral with regard to jurisdictional claims in published maps and institutional affiliations.

## Supplementary Material



Supplementary Information

## Figures and Tables

**Table 1:** Data sources.

**Data Source**	**Description**	**Link**
FAO	Capture Production 1950–2014 (Release date: March 2016)	www.fao.org
ICES	International Committee for the Exploration of the Sea 1950–2014	www.ices.dk
NAFO	Northwest Atlantic Fisheries Organisation Catch and Effort 1960–2014	www.nafo.int
SEAFO	Southeast Atlantic Capture Production 1975–2014 (Release date: June 2016)	www.seafo.org
GFCM	General Fisheries Commission for the Mediterranean Capture production 1970–2014 (Release date: April 2016)	www.gfcm.org
CECAF	Fishery Committee for the Eastern Central Atlantic Capture production 1970–2014 (Release date: May 2016)	http://www.fao.org/fishery/statistics/cecaf-capture-production/2/en
CCAMLR	Commission for the Conservation of Antarctic Marine Living Resources Statistical Bulletin 2016 Vol. 28 1970–2014	www.ccamlr.org
SAUP	Sea Around Us project—records for FAO area 18 (Arctic) v1 1950 TO 2010 (extrapolated to 2014)^18^	www.seaaroundus.org

**Table 2:** Data field definitions.

**Name**	**Long name**	**Units of Measurement**	**Description**
Year	Year (Calendar)	None	Year of fishing (reported)
Seq	Spatial cell code	None	Unique spatial 'half-degree' spatial cell in a gird of 259,200 (360 rows×720 columns) each 0.5 degrees of latitude by 0.5 degrees of longitude. Cell 1 is centered at 90 North and 179.75 West. Numbering is first by row then by column so cell 2 is at 90 N and 179.25 W. Cells numbered from North to South, and within each latitude, from West to East. Centre of each spatial cell is identified by the Lon (Longitude) and Lat (Latitude) fields and the OceanArea field estimates the surface area of ocean within the spatial cell.
Lat	Latitude of centre of spatial cell	Angular degrees in geographical global coordinate system	Centre is.25 degrees from any cell country as these are half degree (or 30 min) spatial cells
Lon	Longitude of centre of spatial cell	Angular degrees in geographical global coordinate system	Centre is.25 degrees from any cell border as these are half degree (or 30 min) spatial cells
OceanArea	Area (estimated) of ocean surface in the spatial cell	Square kilometres	Used to translate catch rates in tonnes per square kilometre of ocean into absolute weights (tonnes)
CNumber	Country Code	None	Code for each country/fishing entity—relates to FAO name, relates to CountryName field
CountryName	Country Name	None	UN’s English name for reporting/fishing country coded by CNumber field
Taxonkey	Taxon Code	None	Code for the reported taxon based on Froese, R., Pauly, D. E., (2006) FishBase. World Wide Web electronic publication.and other sources—relates to TaxonName and CommonName fields
TaxonName	Scientific Taxonomic Name	None	Developed from FishBase and other on-line sources—coded by Taxonkey field
CommonName	Common English Name for taxon	None	Adopted from FishBase and other on-line sources—coded by Taxonkey field
Gear	Fishing gear code	None	Code for the fishing gear used (after von Brandt) see Watson, *et al.*^16^
GearName	Fishing gear name	None	Coded by gear code.
LSF_CR	Large-scale fishing catch rate	Tonnes per sq km of ocean in year	Catch rate of reported landings not attributed to small-scale (SSF_CR) fishing
SSF_CR	Small-scale fisheries catch rate	Tonnes per sq km of ocean in year	Catch rate of small-scale marine fisheries catches based on reported landings and on estimates in Chuenpagdee *et al.*^28^, depending on the reporting accuracy assumed for the country some SSF catch rates might have been reported originally combined with large-scale catch rates and the LLS_CR was adjusted accordingly
IUU_CR	Rate of illegal and otherwise unreported catch	Tonnes per sq km of ocean in year	Catch rate of illegal catch estimated based on Agnew *et al.*^26^
Discards_CR	Rate of associated discards at sea	Tonnes per sq km of ocean in year	Catch rate of discarded marine life estimated based on Kelleher, K.^27^ NOTE: the discards are often not the same taxon as that reported/targeted through fishing so this is only a general estimate with no specific taxonomic identity
