# Supplementary material for: A database of global marine commercial, small-scale, illegal and unreported fisheries catch 1950–2014
Source: Sci Data. 2017 Apr 11;4:170039. doi: 10.1038/sdata.2017.39 (PMC5387926; doi:10.1038/sdata.2017.39)
Supplement: Supplementary Information [file sdata201739-s2.docx]

**Supplemental Information**

**Software and Procedures used to create Global Fisheries Catch dataset**

These notes and code describe the preparation of the Gloal Fisheries dataset.

**1. Sourcing**

Initial processing requires securing the latest public datasets from online sites viz:

**Table 1. Data sources**

| **Data Source** | **Description** | Link |
| --- | --- | --- |
| FAO | Capture Production 1950-2014 (Release date: March 2016) | www.fao.org |
| ICES | International Committee for the Exploration of the Sea 1950-2014 | www.ices.dk |
| NAFO | Northwest Atlantic Fisheries Organisation Catch and Effort 1960-2014 | www.nafo.int |
| SEAFO | Southeast Atlantic Capture Production 1975-2014 (Release date: June 2016) | www.seafo.org |
| GFCM | General Fisheries Commission for the Mediterranean Capture production 1970-2014 (Release date: April 2016) | www.gfcm.org |
| CECAF | Fishery Committee for the Eastern Central Atlantic Capture production 1970-2014 (Release date: May 2016) | www.fao.org/fishery/rfb/ecaf |
| CCAMLR | Commission for the Conservation of Antarctic Marine Living Resources Statistical Bulletin 2016 Vol. 28 1970-2014 | www.ccamlr.org |
| CATCH | Sea Around Us project – records for FAO area 18 (Arctic) v1 1950 TO 2010 (extrapolated to 2014) | www.seaaroundus.org |

Most of these sites update their data annually. They may revise any of their material from any year in any subsequent year without reporting this. Similarly they may recode. Generally more taxa are included in reporting each year.

Some of these dataset are in the FishStatJ format and require FAO’s java-based software to extract the contents. The site is <http://www.fao.org/fishery/statistics/software/fishstatj/en>.

Here are current notes on preparing the datasets for recoding and compilation:

These have to be flattened to a csv with all necessary fields represented. These then have to encoded with codes for each reported taxa (those not included such as freshwater fishes or algae deleted), country (might have to be updated), FAO area etc.

Install the latest FishStatJ (datasets may not work with older versions). Usually requires the Windows operating system. It allows you to download the datasets such as global capture and regional captures. Load each. Right-hand mouse click allows selection of all data. Use menu to export to .csv file to the appropriate original file folder. Open these in Excel and export as .txt tab-delimited form into the processed folder.

Typically the Global Capture dataset is done first. The regional datasets are used to replace data from matching FAO statistical areas as these offer better spatial definition and may offer more detail in general.

Keep the original files intact in a separate daughter folder (original). Take each csv and export it in Excel as a tab delimited txt files in a separate daughter folder (processed). This avoids problems with embedded commas in fields. Text characters in what should be strictly numeric fields is a problem but so are commas embedded in text fields.

See **code section #1** for Microsoft visual basic code which reads and converts fields to codes, and exports a compiled, flattened CSV file.

The function uses txt (tab or semicolon delimited) rather than comma-delimited (csv) files to avoide the problem of embedded commas in the conversion between text strings representing countries, taxon and areas – and their equivalent codes in the system. It will check to see which text strings are not found and list these so they can be added. This will involve several passes (each one is very quick) as new codes and their conversion are added.

Taxa – often common names but sometime three letter codes must be matched to a common taxon code. Each taxonomic entry must be in the matching list, even for plants and freshwater taxa - even if the code is 999999 and therefore ignored. Remove any “ in the exported tab-delimited text file (from the xls).

A ‘zero’ entry in data supplied in table format is assumed to be zero and no record is written to the flat or assembled global dataset. If there is a zero provided in a provided flattened file format then it is assumed to be a minimum weight (100 kg) but non-zero weight, as there would be no reason to have a real zero appearing in this format.

Careful that you end up with the right delimits (usually semicolon) on the datafiles. Typically work on xls/.csv version then change in text editor replacing, for example tabs with semicolons.

Careful new ICES columns appear in reverse ordered year-wise.

CCAMLR comes as a macro-driven mdb file. These do not open on all machines and are for windows only. You must stop the macro running on start up by pressing shift. Then you can use the queries inside like queryTable9_1 to get the records which you can cut and paste into excel.

In order to summarise it I used a query based on their query

SELECT qryTable09_1.ASD, qryTable09_1.SpeciesCode, qryTable09_1.CountryCode, qryTable09_1.SeasonYear, Sum(qryTable09_1.[CatchWeight(t)]) AS [SumOfCatchWeight(t)]

FROM qryTable09_1

GROUP BY qryTable09_1.ASD, qryTable09_1.SpeciesCode, qryTable09_1.CountryCode, qryTable09_1.SeasonYear

ORDER BY qryTable09_1.SeasonYear;

Results can be pasted into Microsof Excel, and saved as a tab-delimited file for input.

The assembled dataset is Catchout.csv (comma-delimited) and this must be imported in to the CATCH.mdb access database, copied or renamed as CATCH2, and indexed (ID field added) so that further processing can proceed. Ensure this is in the correct directory and that all links to external supporting tables are updated and work.

In the CATCH2 data table made from the Catchout.csv produced by this function, the CatchID is a continuous ID across all data sources as they are parsed, flattened and written to source files. These are unique (before disaggregation of taxa) but not all these records and therefore catchIDs are used in the Catchout.csv (which becomes the CATCH2 mdb table).

As some of the incoming source data lines are selected to join the aggregate global dataset, each one has a unique and incremental CATCHID field added. For CATCHID this is a continuous and unique identifier until some records are removed by the function that Checks CATCH2.

This function takes about 10 min to run.

===============================================

**2. Compilation**

Once the datasets have been flattened and coded, they can be combined. The procedure is that the FAO capture dataset forms the ‘backbone’ and for individual major FAO statistical reporting areas their data is selectively replaced with datasets that provide more detailed coverage such as from regional RFMOs. Once this compilation is created there are data that much be filtered out. Note that properly this dataset only describes **landings** – reported and landed global statistics which misses some small scale fishing and does not account for catches that are unreported (whether illegal or not) and those catches that are discarded at sea.

See **code section #2** Compilation:

Code takes the access catch database CATCH.mdb and performs a series of steps on the CATCH2 table. Each one is documented in a text file report that is produced.

Step 1. Remove Unwanted Groups (removeUnwantedGroups function)

Uses query "DELETE CATCH2.* FROM CATCH2 INNER JOIN TaxonNom ON CATCH2.TaxonKey = TaxonNom.TaxonKey WHERE ((TaxonNom.ISSCAAP<23) Or (TaxonNom.ISSCAAP In (41,51,61,62,63,64,71,72,73,77)) Or (TaxonNom.ISSCAAP>77))"

Which ensures:

1) No freshwater fish (Isscaap=22)

2) No freshwater crustacean (Isscaap=41)

3) No freshwater molluscs (Isscaap=51)

4) No higher ISSCAAP groups except for Sea-squirts and other tunicates; Horseshoe crabs and other arachnoids; Sea-urchins and other echinoderms

5) No Artemia salina

Step 2. Correct Country (correctCountry function)

Step 3. Correct Taxonomy (correctTaxonomy function)

Step 4. Correct Areas (correctArea function)

Step 5. **Check Integrity** of CATCH2 (checkCATCH2 function)

Makes a report that shows potential problems

This function (even with the optional integrity reporting) takes only minutes to run

**3. Disaggregation**

Before mapping is attempted there must be adjustments to the dataset records. One such process is called taxonomic disaggregation. For records where the reported taxon is more general than a Family of animals (ie Order, Class or one of FAO’s ISSCAAP groups such as Misc Marine Fishes) there is an attempt (described in publications and shown in code provided) to prorate these landed tonnages to a series of likely candidates which are suggested by more fully described statistics of other countries fishing in the same large marine ecosystem (ie likely encountering similar fishing targets). At this stage the original reported taxon is kept along with any new more specific prorated portion so that changes at this stage are documented and the actual reported taxa will be known. The number of data records after this process will increase, as what was once one landing from an aggregated taxon like an Order of fish, becomes a series of smaller more specific one (families, genera and species). Note, it is not always possible to find suitable candidates for the taxonomic disaggregation and if so the record is left unmodified.

See **code section #3** Disaggregation

By disaggregating the CATCH2 records and adding fields it makes the CATCH3 mdb table that is used in the mapping allocation procedures.

Takes **2-3 hours** to run.

Puts a file CATCH3.CSV in the **output directory location.** This can be set in the program code. The text fields are “ delimited. **It has to be imported into the CATCH.mdb database with a new index ID added.**

In CATCH3 you have to add fields Distant (Yes/No), Prob (Yes/No) and Added (Double) to support further processing.

**4. Finalizing Pre-Mapped Compilation and Auxiliary Data Preparation**

The records of Catch3.csv have a boolean field called Distant, which is set to TRUE when a country reports landings from an area outside of their waters.

See **code section #4**

For each taxon in the Catch3 table there must be a global distribution of its occurrence. This can be static or annual. Simple extrapolation and interpolation will be used. The format requires that for each 30-min spatial cell the proportion of its global occurrence be provided (for that year). For most taxa this is a static representation informed by FishBase (references) or other sources. For the major tuna and bill fish species these is prepared on an annual bases using the spatial information they provide on their webpages. These are annual harvest distributions are they are only a portion of the species global distribution.

For each reporting country in the Catch3 table there must be a record which describes when the country began fishing outside of its EEZ claimed waters, and when it began to fish on the highseas. If these dates did not occur then it is coded as 9999.

For each reporting country there must be a record of which other countries their fleets have arrangements to fish in or have been observed (if agreement not known) to fish in. These permissions are by broad groups of species (called Target groups – example ‘small pelagics’) taken by the same gear types. This information can pertain to all or a subset of the fished taxa. In addition, post-processing may have to enforce quotas associated with fishing access agreements (see post-possessing).

**5. Mapping**

See **code section #5**

The records of Catch3, using the information about the global distribution of fished taxa (and for tuna and billfish the annual harvest distributions) and the access of the reporting country’s fleets to EEZ claimed waters, are mapped to a network of 30-min spatial cells. The amount of ocean represented in each 30-min spatial cell is known. This varies because some are coastal or have islands, and they decrease in size toward the poles with maximum sizes at the equators. Therefore the weight of associated landings is prorated to valid cells (meeting conditions of taxon distribution and fishing access) within the reporting area provided by the data source, and this is adjusted to represent the catch rate (tonnes per sq kilometer of ocean) in the cell. To maintain accuracy with lower data storage requirements this is multiplied by 100,000 and integers stored.

The data is prepared in 5-year blocks to allow easier manipulation later.

The mapping process has to proceed sequentially from 1950 to the final year (2014 in the current version). This is because there is a schedule in place (representing logistic and political momentum) that controls the uptake of foreign fishing grounds by national fleets ie when an agreement is signed there is only an initially small but increasing portion of a national fishing fleet which takes up the option. This means that the previous year’s situation influences the current year’s distribution of fishing effort forcing sequential processing.

The mapping process takes multiple days to run on a powerful laptop. It uses all the source landings data, and the supporting databases on taxon distribution, fleet access and statistical areas to prorate the tonnages in the landings to output files which represent the catch rate achieved in each spatial cell, for that incoming record (year, fishing country, fished taxon and area fished). These output files are understandably large and to be further processed are imported (bulk process) to a Microsoft sqlserver database where the post-processing takes place.

**6. Post-Processing**

Mapping will not succeed for all incoming records. For some, the location provided either does not overlap with the known distribution of the landed taxon, or the fishing fleets reporting it do not have known access to these waters (typically the inshore EEZ claim of another country). These are marked as problems and with the help of special mapping software the various necessary elements can be visualized and decisions taken on how to proceed. The provided data might be in error or the supporting databases used in the mapping may be insufficient or outdated. Typically less than 0.005% of records will be left unresolved and omitted from the mapped dataset. These will amount to an insignificant portion of the annual catch and well within the margins of error normally associated with the data sources themselves.

Once the mapped landings are presented in .csv files these are imported to an sqlserver for further processing.

See **code section #6**

Some areas such as within an EEZ claims might allow legal fishing by foreign fleets but limit their landings via quotas. This is integrated into the reporting by many groups like ICES, ie they will not report landings that exceed quotas. For other jurisdictions, access arrangements associated with known quotas must be applied to the mapped data after the initial mapping to spatial cells. This is done by adjusting catches within the quota area for the species involved until the quota is not exceeded. The extra quota catch is distributed proportionally to increase catch reporting values outside of quota areas. No catch is lost in this process and it is only a spatial redistribution.

The mapped catch is associated with commercial fishing gear type as in:

Watson, R., Revenga, C., Kura, Y. (2006) *Fishing gear associated with global marine catches. II. Trends in trawling and dredging.* Fisheries Research **79,** 103-111.

Watson, R., Revenga, C., Kura, Y. (2006.) *Fishing gear associated with global marine catches: I Database development*. Fisheries Research **79,** 97-102.

A process is applied that prorates the mapped catch dataset into another dataset that is associated with coded fishing gear types. This increases the number of database records but does not change the weight of catch represented.

For various purposes the catch database can be summarised to catch by Large Marine Ecosystems or by Exclusive Economic Zone claims by combining the 30-min spatial cells. Note that EEZ claims are often overlapping (particularly in the Asian Pacific region) therefore summaries of this sort may have to represent the same catch form a single area as part of the total for several countries that claim that same ocean area.

**7. Estimating Unreported Catch**

For further processing, the CatchGear table in the sqlserver database is exported as a series of 5-year blocks in comma-delimited csv files. These are similar to those made public, however, at this stage they only have one field for catch (landings) expressed as a catch rate (tonnes per sq km of ocean in the year) and have only the codes for country, taxon and gear type but their text descriptors have not been added.

See **code section #7**

Estimates are made of unreported catch (whether illegal or not) and discarded catch. See:

Agnew DJ, Pearce J, Pramod G, Peatman T, Watson R, Beddington JR, et al. *Estimating the worldwide extent of illegal fishing*. PLoS One. 2009;4(2):e4570. doi: 10.1371/journal.pone.0004570

Kelleher, K. (2005) *Discards in the world’s marine fisheries. An update*. FAO Fisheries Technical Paper, 131.

and for the origin of the factors appearing in the code.

Further, the catch in locations mapped to small scale fisheries in sensu of

Chuenpagdee, R., Liguori, L., Palomares, M.L.D., Pauly, D. (2006) *Bottom up, global estimates of small-scale marine fisheries catches*. Fisheries Centre Research Reports, 105pp.

from shallow and near shore areas but not associated with large-scale industrial fishing gears such a tuna purse sein is examined. If these small-scale landings are under represented in the dataset then they are augmented (each relevant record proportionately) otherwise the expected values in the dataset are hence forth designated as small-scale. The remainder, not classified as small-scale is designated as large-scale. That is, a general catch value is separated into large and small-scale portions based on where the fishing occurred and what gears were used. If small-scale were under represented then the catch rates for this sector was augmented accordingly.

**8. Addition of Description Fields**

After each record has an estimate of small-scale, large-scale, IUU (unreported) catch and discards added, it is further modified to add the text descriptors useful to users (ie to eliminate the need to translate the codes with associated tables). Though it makes the data ‘stand-alone’ it unfortunately makes the 5-year block csv files much larger. They are then placed in an achive at the University of Tasmania (IMAS) and a permalink / DOI assigned. The download links are included in the achives metarecord. Watson, R.A. *A database of global marine commercial, small-scale, illegal and unreported fisheries catch 1950-2014.*  <http://dx.doi.org/10.4226/77/58293083b0515>

**CODE USED FOR PROCESSING GLOBAL CATCH DATASET**

**Microsoft Visual Basic 2010**

**1. Sourcing**

Function Catch_Import() As Boolean

Dim MaxRecords As Long = 100000

Dim MaxCodes As Long = 10000

Dim ColOffset As Long = 0

Dim SkipLines As Long = 0

Dim CommodityCol As Long = 0

Dim CountryCol As Long = 0

Dim Delim As String = ""

Dim FileIn As String = ""

Dim FileOut As String = ""

Dim RCodeCol As Long = 0

Dim RCodeStr As String = ""

Dim ICodeCol As Long = 0

Dim ICodeStr As String = ""

Dim ECodeCol As Long = 0

Dim ECodeStr As String = ""

Dim TypeCol As Long = 0

Dim TypeStr As String = ""

Dim FirstYear As Long = 0

Dim LastYear As Long = 0

Dim Instr As String = ""

Dim strBits() As String

Dim NumCol As Long

Dim StrNum As Long = 0

Dim CatchStr As String = ""

Dim Year As Long = 0

Dim Quote As String = """"

Dim Countlines As Long = 0

Dim NumRecords As Long = 0

Dim IYear As Long

Dim CurCol As Long

Dim YearStr As String = ""

Dim CountryStr As String = ""

Dim CountryFile As String

Dim CName(500) As String

Dim CNumbers(500) As Long

Dim CountryName As String

Dim LastCountryName As String = "XXXX"

Dim TaxonName As String = ""

Dim EnvironName As String = ""

Dim TaxonStr As String = ""

Dim TName(MaxCodes) As String

Dim TCode(MaxCodes) As Long

Dim LastTaxonName As String = "XXXX"

Dim AreaName As String

Dim LastAreaName As String = "XXXX"

Dim AName(MaxCodes) As String

Dim ACode(MaxCodes) As Long

Dim FAOCode(MaxCodes) As Long

Dim QuotePos As Long

Dim TaxonMissing As Boolean = False

Dim AreaMissing As Boolean = False

Dim CountryMissing As Boolean = False

Dim strLine As String

Dim NumCountries As Long = 0

Dim TaxonFile As String

Dim AreaFile As String

Dim NumTaxon As Long = 0

Dim NumArea As Long = 0

Dim MissingCountry As String

Dim MissingArea As String

Dim MissingTaxon As String

'Receiving fields

Dim InCountry(MaxRecords) As String

Dim InTaxon(MaxRecords) As String

Dim Environ(MaxRecords) As String

Dim InArea(MaxRecords) As String

Dim InYear(MaxRecords) As String

Dim InTonnes(MaxRecords) As String

Dim CurRecord As Long

Dim CNumber As Long

Dim FAO As Long

Dim TaxonNum As Long

Dim AreaNum As Long

Dim I As Long

Dim Source As Long

Dim InValues(,) As String

Dim RequiredFAO As Long

Dim TableIn As Boolean = False

Dim Tonnes As Double

Dim CFishingField As Long

Dim AFishingField As Long

Dim TFishingField As Long

Dim YFishingField As Long

Dim EnvironField As Long

Dim ExtrapolatedYear As Long

Dim DoSegment(100, 2050, 100) As Boolean

Dim OtherSource As Long

Dim SourceLabel(100) As String

Dim ShortLabel(100) As String

Dim DoSource(100) As Boolean

Dim ProcessedAlready As Boolean

Dim OutputFile As String

Dim MinimumCatch As Double = 0.1

Dim CatchID As Long = 0

Dim CATCHID As Long = 0

Catch_Import = False

Windows.Forms.Cursor.Current = Windows.Forms.Cursors.WaitCursor

'Which Sources to include in run ===================

ShortLabel(1) = "FAO"

SourceLabel(1) = "FAO Capture"

DoSource(1) = True 'FAO Capture

ShortLabel(3) = "ICES1"

SourceLabel(3) = "ICES Historical"

DoSource(3) = True 'ICES Historical

ShortLabel(4) = "ICES2"

SourceLabel(4) = "ICES Recent"

DoSource(4) = True 'ICES Recent

ShortLabel(13) = "NAFO"

SourceLabel(13) = "NAFO"

DoSource(13) = True 'NAFO

ShortLabel(23) = "SACP"

SourceLabel(23) = "SACP formerly SEAFO"

DoSource(23) = True 'SACP formerly SEAFO

ShortLabel(24) = "GFCM"

SourceLabel(24) = "GFCM Mediterranean and Black Sea"

DoSource(24) = True 'GFCM Mediterranean and Black Sea

ShortLabel(25) = "CECAF"

SourceLabel(25) = "CECAF Eastern Central Atlantic"

DoSource(25) = True 'CECAF Eastern Central Atlantic

ShortLabel(30) = "CCAMLR"

SourceLabel(30) = "CCAMLR"

DoSource(30) = True 'CCAMLR

ShortLabel(31) = "RECOFI"

SourceLabel(31) = "RECOFI"

DoSource(31) = False 'RECOFI DO NOT USE !!!!!!!!

ShortLabel(44) = "MAC"

SourceLabel(44) = "Macquarie"

DoSource(44) = True 'Macquarie

ShortLabel(50) = "ARCTIC"

SourceLabel(50) = "CATCHWEB Arctic"

DoSource(50) = True 'CATCHWEB Arctic

ShortLabel(98) = "TRADE"

SourceLabel(98) = "FAO Trade"

DoSource(98) = False 'Aquaculture

ShortLabel(99) = "AQUACULTURE"

SourceLabel(99) = "FAO Aquaculture"

DoSource(99) = False 'Aquaculture

'===================================================

Dim Folder As String = UserSource + "\Landings\"

MissingCountry = Folder + "MissingCountry.csv"

MissingTaxon = Folder + "MissingTaxon.csv"

MissingArea = Folder + "MissingArea.csv"

CountryFile = Folder + "CountryNames.txt" 'semicolon

TaxonFile = Folder + "Taxalist.txt" 'tab

AreaFile = Folder + "Arealist.txt" 'semicolon

'harmonised output file

OutputFile = Folder + "CatchOut.csv"

Dim swo As IO.StreamWriter = New IO.StreamWriter(OutputFile)

swo.WriteLine("CATCHID, CatchID, Year,CNumber,Taxonkey,AreaCode,FAOArea,Catch,Source")

Dim swMissingTaxon As IO.StreamWriter = New IO.StreamWriter(MissingTaxon)

swMissingTaxon.WriteLine("Taxon,Source,Record")

swMissingTaxon.Flush()

Dim swMissingCountry As IO.StreamWriter = New IO.StreamWriter(MissingCountry)

swMissingCountry.WriteLine("Country,Source,Record")

swMissingCountry.Flush()

Dim swMissingArea As IO.StreamWriter = New IO.StreamWriter(MissingArea)

swMissingArea.WriteLine("Area,Source,Record")

swMissingArea.Flush()

'READ IN COUNTRY NAMES LIST SO THEY CAN BE MATCHED AND A CNUMBER OBTAINED

Dim srcountry As IO.StreamReader = New IO.StreamReader(CountryFile)

Instr = srcountry.ReadLine 'labels'ID CName CNumber

NumCountries = 0

Do While srcountry.Peek <> -1

Instr = srcountry.ReadLine

NumCountries = NumCountries + 1

strBits = Instr.Split(";")

CountryName = strBits(1)

'remove any quotes

Do Until CountryName.Contains(Quote) = False

QuotePos = CountryName.IndexOf(Quote)

If QuotePos = 0 Then

CountryName = CountryName.Substring(1)

Else

CountryName = CountryName.Substring(0, QuotePos)

End If

Loop

CName(NumCountries) = CountryName

CNumbers(NumCountries) = CLng(strBits(2))

Loop

'READ IN TAXON NAMES

Dim srtaxon As IO.StreamReader = New IO.StreamReader(TaxonFile)

Instr = srtaxon.ReadLine 'labels'ID CName CNumber

NumTaxon = 0

Do While srtaxon.Peek <> -1

Instr = srtaxon.ReadLine

NumTaxon = NumTaxon + 1

strBits = Instr.Split(";")

TaxonName = strBits(0)

'remove any quotes

Do Until TaxonName.Contains(Quote) = False

QuotePos = TaxonName.IndexOf(Quote)

If QuotePos = 0 Then

TaxonName = TaxonName.Substring(1)

Else

TaxonName = TaxonName.Substring(0, QuotePos)

End If

Loop

TName(NumTaxon) = TaxonName

TCode(NumTaxon) = CLng(strBits(1))

Loop

'READ IN AREAS NAMES

Dim srarea As IO.StreamReader = New IO.StreamReader(AreaFile)

Instr = srarea.ReadLine 'labels'ID CName CNumber

NumArea = 0

Do While srarea.Peek <> -1

Instr = srarea.ReadLine

NumArea = NumArea + 1

strBits = Instr.Split(";")

AreaName = strBits(0)

'remove any quotes

Do Until AreaName.Contains(Quote) = False

QuotePos = AreaName.IndexOf(Quote)

If QuotePos = 0 Then

AreaName = AreaName.Substring(1)

Else

AreaName = AreaName.Substring(0, QuotePos)

End If

Loop

AName(NumArea) = AreaName

ACode(NumArea) = CLng(strBits(1))

If strBits(2) <> "" Then

FAOCode(NumArea) = CLng(strBits(2))

Else

FAOCode(NumArea) = 0

End If

Loop

'=====================================================================================

'set defaults for writing to harmonised file

For Source = 1 To 100

For IYear = 1950 To 2050

For AreaNum = 1 To 100

DoSegment(Source, IYear, AreaNum) = False

Next AreaNum

Next IYear

Next Source

For Source = 100 To 1 Step -1

Select Case Source

Case 1 'FAO Capture

If DoSource(Source) = False Then GoTo NextSource

ColOffset = 4

FirstYear = 1950

LastYear = 2014

FileIn = Folder + "FAO\Processed\Global FAO Capture 2016.txt" 'tab delimited

FileOut = Folder + "FAO\Processed\Capture flattened New.csv"

RequiredFAO = 0 'if just one FAO area needed in composite

TableIn = True

CFishingField = 0

TFishingField = 1

AFishingField = 2

YFishingField = -1

ExtrapolatedYear = -1

ProcessedAlready = False

'As all other sources have been done first - FAO will do what it left to do

'Except for Macquarie Is data source (44) do not do FAO if other sources used

For IYear = FirstYear To LastYear

For AreaNum = 1 To 100

DoSegment(1, IYear, AreaNum) = True

Next AreaNum

Next IYear

''GoTo TakeitAll 'do not turn off any FAO data for alternatives.

For OtherSource = 2 To 100

For IYear = FirstYear To LastYear

For AreaNum = 1 To 100

If OtherSource <> 44 Then

If DoSegment(OtherSource, IYear, AreaNum) = True Then

'do not include overlaps (except Macquarie) (all turned on above then some turned off)

DoSegment(1, IYear, AreaNum) = False

End If

End If

Next AreaNum

Next IYear

Next OtherSource

TakeitAll:

Case 3 'ICES Historical

If DoSource(Source) = False Then GoTo NextSource

ColOffset = 3

FirstYear = 1950

LastYear = 2010

FileIn = Folder + "ICES\Processed\ICES1.txt" 'tab delimited

FileOut = Folder + "ICES\Processed\ICES1 flattened.csv"

RequiredFAO = 27

TableIn = True

CFishingField = 0

TFishingField = 1

AFishingField = 2

YFishingField = -1

ExtrapolatedYear = -1

ProcessedAlready = False

For IYear = FirstYear To 2005

DoSegment(Source, IYear, 27) = True

Next

Case 4 'ICES Recent

If DoSource(Source) = False Then GoTo NextSource

ColOffset = 4

FirstYear = 2006

LastYear = 2014

FileIn = Folder + "ICES\Processed\ICES2.txt" 'tab delimited

FileOut = Folder + "ICES\Processed\ICES2 flattened.csv"

RequiredFAO = 27

TableIn = True

CFishingField = 3

TFishingField = 0

AFishingField = 1

YFishingField = -1

ExtrapolatedYear = -1

ProcessedAlready = False

For IYear = FirstYear To LastYear

DoSegment(Source, IYear, 27) = True

Next

Case 13 'NAFO

If DoSource(Source) = False Then GoTo NextSource

ColOffset = 5

FirstYear = 1960

LastYear = 2014

FileIn = Folder + "NAFO\Processed\STATLANT21A_Extraction.txt" 'tab delimited

FileOut = Folder + "NAFO\Processed\STATLANT21A_Extraction flattened.csv"

RequiredFAO = 21

TableIn = False

CFishingField = 1

TFishingField = 4

AFishingField = 2

YFishingField = 0

ExtrapolatedYear = -1

ProcessedAlready = False

For IYear = FirstYear To LastYear

DoSegment(Source, IYear, 21) = True

Next

Case 23 'SACP formerly SEAFO Southeast Atlantic capture production

If DoSource(Source) = False Then GoTo NextSource

ColOffset = 4

FirstYear = 1975

LastYear = 2014

FileIn = Folder + "FAO\Processed\SEACP 2016.txt" 'tab delimited

FileOut = Folder + "FAO\Processed\SEACP flattened.csv"

RequiredFAO = 47

TableIn = True

CFishingField = 0

TFishingField = 1

AFishingField = 2

YFishingField = -1

ExtrapolatedYear = -1

ProcessedAlready = False

For IYear = FirstYear To LastYear

DoSegment(Source, IYear, 47) = True

Next

Case 24 'GFCM Mediterranean and Black Sea capture production

If DoSource(Source) = False Then GoTo NextSource

ColOffset = 4

FirstYear = 1970

LastYear = 2014

FileIn = Folder + "FAO\Processed\GFCM 2016.txt" 'tab delimited

FileOut = Folder + "FAO\Processed\GFCM flattened.csv"

RequiredFAO = 37

TableIn = True

CFishingField = 0

TFishingField = 1

AFishingField = 2

YFishingField = -1

ExtrapolatedYear = -1

ProcessedAlready = False

For IYear = FirstYear To LastYear

DoSegment(Source, IYear, 37) = True

Next

Case 25 'CECAF Eastern Central Atlantic capture production

If DoSource(Source) = False Then GoTo NextSource

ColOffset = 4

FirstYear = 1970

LastYear = 2014

FileIn = Folder + "FAO\Processed\CECAF 2016.txt" 'tab delimited

FileOut = Folder + "FAO\Processed\CECAF flattened.csv"

RequiredFAO = 34

TableIn = True

CFishingField = 0

TFishingField = 1

AFishingField = 2

YFishingField = -1

ExtrapolatedYear = -1

ProcessedAlready = False

For IYear = FirstYear To LastYear

DoSegment(Source, IYear, 34) = True

Next

Case 30 'CCAMLR

If DoSource(Source) = False Then GoTo NextSource

ColOffset = 4

FirstYear = 1970 'was 1969

LastYear = 2014

FileIn = Folder + "CCAMLR\processed\CCAMLR.txt" 'tab delimited

FileOut = Folder + "CCAMLR\processed\CCAMLR flattened.csv"

RequiredFAO = 0 'several FAO areas involved

TableIn = False

CFishingField = 2

TFishingField = 1

AFishingField = 0

YFishingField = 3

ExtrapolatedYear = -1

ProcessedAlready = False

For IYear = FirstYear To LastYear

DoSegment(Source, IYear, 48) = True

DoSegment(Source, IYear, 58) = True

DoSegment(Source, IYear, 88) = True

Next

Case 31 'RECOFI ==========DO NOT USE AS ONLY A SMALL AREA COVERED

If DoSource(Source) = False Then GoTo NextSource

ColOffset = 4

FirstYear = 1986

LastYear = 2014

FileIn = Folder + "FAO\Processed\RECOFI 2016.txt" 'tab delimited

FileOut = Folder + "FAO\Processed\RECOFI flattened.csv"

RequiredFAO = 51

TableIn = True

CFishingField = 0

TFishingField = 1

AFishingField = 2

YFishingField = -1

ExtrapolatedYear = -1

ProcessedAlready = False

For IYear = FirstYear To LastYear

DoSegment(Source, IYear, 51) = True

Next

Case 44 'Macquarie

If DoSource(Source) = False Then GoTo NextSource

ColOffset = 5

FirstYear = 1994

LastYear = 2014

CFishingField = 1

TFishingField = 2

AFishingField = 3

YFishingField = 0

'already in final format - extrapolated from ABARES status report to 2013-2014 season catches

FileIn = Folder + "Macquarie\Processed\Macquarie flattened.txt" 'tab delimited

FileOut = Folder + "Macquarie\Processed\Dummy.txt" 'tab delimited

RequiredFAO = 81

TableIn = False

ProcessedAlready = True

For IYear = FirstYear To LastYear

DoSegment(Source, IYear, 81) = True

Next

Case 50 'CATCHWEB - ARCTIC

If DoSource(Source) = False Then GoTo NextSource

ColOffset = 11

FirstYear = 1950

LastYear = 2010

FileIn = Folder + "CATCH Arctic\Processed\SAU_Arctic.txt" 'tab delimited

FileOut = Folder + "CATCH Arctic\Processed\SAU_Arctic flattened.csv"

RequiredFAO = 18

TableIn = False

CFishingField = 7

TFishingField = 3

AFishingField = 0

YFishingField = 2

ExtrapolatedYear = 2014

ProcessedAlready = False

For IYear = FirstYear To LastYear

DoSegment(Source, IYear, 18) = True

Next

Case 98 'FAO Trade

If DoSource(Source) = False Then GoTo NextSource

ColOffset = 4

FirstYear = 1976

LastYear = 2013 'NOTE

FileIn = Folder + "FAO\Processed\Global FAO Trade 2016.txt" 'tab delimited

FileOut = Folder + "FAO\Processed\Trade flattened New.csv"

RequiredFAO = 0 'if just one FAO area needed in composite

TableIn = True

CFishingField = 0

TFishingField = 1

AFishingField = 3

YFishingField = -1

EnvironField = 2

ExtrapolatedYear = -1

ProcessedAlready = False

For IYear = FirstYear To LastYear

For AreaNum = 1 To 100

DoSegment(1, IYear, AreaNum) = True

Next AreaNum

Next IYear

Case 99 'FAO Aquaculture

If DoSource(Source) = False Then GoTo NextSource

ColOffset = 5

FirstYear = 1950

LastYear = 2014

FileIn = Folder + "FAO\Processed\Global FAO Aquaculture 2016.txt" 'tab delimited

FileOut = Folder + "FAO\Processed\Aquaculture flattened New.csv"

RequiredFAO = 0 'if just one FAO area needed in composite

TableIn = True

CFishingField = 0

TFishingField = 1

AFishingField = 2

YFishingField = -1

EnvironField = 3

ExtrapolatedYear = -1

ProcessedAlready = False

For IYear = FirstYear To LastYear

For AreaNum = 1 To 100

DoSegment(1, IYear, AreaNum) = True

Next AreaNum

Next IYear

Case Else

GoTo Nextsource

End Select

NumCol = (LastYear - FirstYear) + ColOffset + 1

ReDim InValues(MaxRecords, NumCol - 1)

Countlines = 0

'READ IN FAO SO DATA CAN BE MATCHED AND GET CODES

Dim src As IO.StreamReader = New IO.StreamReader(FileIn)

Instr = src.ReadLine 'labels

'Country (Country) Species (ASFIS species) Fishing area (FAO major fishing area) Measure (Measure) 1950 1951 1952 1953 1954 1955 1956 1957 1958 1959 1960 1961 1962 1963 1964 1965 1966 1967 1968 1969 1970 1971 1972 1973 1974 1975 1976 1977 1978 1979 1980 1981 1982 1983 1984 1985 1986 1987 1988 1989 1990 1991 1992 1993 1994 1995 1996 1997 1998 1999 2000 2001 2002 2003 2004 2005 2006 2007 2008 2009 2010 2011 2012 2013"

NumRecords = 0

Do While src.Peek <> -1

Instr = src.ReadLine

If Instr.Contains("Totals") Then Exit Do 'summaries at bottom

NumRecords = NumRecords + 1

strBits = Instr.Split(vbTab)

InCountry(NumRecords) = strBits(CFishingField)

InTaxon(NumRecords) = strBits(TFishingField)

InArea(NumRecords) = strBits(AFishingField)

If Source = 98 Or Source = 99 Then

Environ(NumRecords) = strBits(EnvironField)

End If

If TableIn = False Then

InYear(NumRecords) = strBits(YFishingField)

InTonnes(NumRecords) = strBits(ColOffset)

Else

'field 3 is just the units

For IYear = FirstYear To LastYear

CurCol = (IYear - FirstYear) + ColOffset

InValues(NumRecords, IYear - FirstYear) = strBits(CurCol)

Next

End If

Loop

Dim sw As IO.StreamWriter = New IO.StreamWriter(FileOut)

'changed Oct 19 2016 to allow an extra field for aquaculture data

If DoSource(99) = False And DoSource(98) = False Then

sw.WriteLine("CatchID,Year,CNumber,Taxonkey,AreaCode,FAOArea,Catch,Source")

Else

sw.WriteLine("CatchID,Year,CNumber,Environ,Taxonkey,AreaCode,FAOArea,Catch,Source")

End If

'Process indata read - match codes etc.

'Test Countries

For CurRecord = 1 To NumRecords

'country =============================

CountryStr = InCountry(CurRecord)

If ProcessedAlready = False Then

'strip off quotes

CountryName = CountryStr

Do Until CountryName.Contains(Quote) = False

QuotePos = CountryName.IndexOf(Quote)

If QuotePos = 0 Then

CountryName = CountryName.Substring(1)

Else

CountryName = CountryName.Substring(0, QuotePos)

End If

Loop

CNumber = 0

For I = 1 To NumCountries

If CName(I) = CountryName Then

CNumber = CNumbers(I)

Exit For

End If

Next

If CNumber = 0 Then

CountryMissing = True

If LastCountryName <> CountryName Then

swMissingCountry.WriteLine(Quote + CountryName + Quote + "," + SourceLabel(Source) + "," + CStr(CurRecord))

swMissingCountry.Flush()

LastCountryName = CountryName

End If

End If

Else

CNumber = CLng(CountryStr)

End If

'taxon =============================

TaxonStr = InTaxon(CurRecord)

If ProcessedAlready = False Then

'strip off quotes

TaxonName = TaxonStr

Do Until TaxonName.Contains(Quote) = False

QuotePos = TaxonName.IndexOf(Quote)

If QuotePos = 0 Then

TaxonName = TaxonName.Substring(1)

Else

TaxonName = TaxonName.Substring(0, QuotePos)

End If

Loop

TaxonNum = 0

For I = 1 To NumTaxon

If TName(I).ToUpper = TaxonName.ToUpper Then

TaxonNum = TCode(I)

Exit For

End If

Next

If Source <> 99 And Source <> 98 Then

If TaxonNum = 0 Then

TaxonMissing = True

If LastTaxonName <> TaxonName Then

swMissingTaxon.WriteLine(Quote + TaxonName + Quote + "," + SourceLabel(Source) + "," + CStr(CurRecord))

swMissingTaxon.Flush()

LastTaxonName = TaxonName

End If

ElseIf TaxonNum > 999998 Then

GoTo nextrec 'taxon not included

End If

Else

'Aquaculture and Trade

'don't have taxoncodes yet

TaxonNum = 999999

End If

Else

TaxonNum = CLng(TaxonStr)

End If

'Area =============================

AreaName = InArea(CurRecord)

If ProcessedAlready = False Then

'strip off quotes

Do Until AreaName.Contains(Quote) = False

QuotePos = AreaName.IndexOf(Quote)

If QuotePos = 0 Then

AreaName = AreaName.Substring(1)

Else

AreaName = AreaName.Substring(0, QuotePos)

End If

Loop

AreaNum = 0

For I = 1 To NumArea

If AName(I).ToUpper = AreaName.ToUpper Then

If RequiredFAO <> 0 Then

If FAOCode(I) <> RequiredFAO Then

GoTo NextArea

End If

End If

AreaNum = ACode(I)

FAO = FAOCode(I)

Exit For

End If

NextArea:

Next

If Source = 99 Or Source = 98 Then 'trade or aquaculture - added for Julia NEE Oct 2016

'Environment =============================

EnvironName = Environ(CurRecord)

If ProcessedAlready = False Then

'strip off quotes

Do Until EnvironName.Contains(Quote) = False

QuotePos = EnvironName.IndexOf(Quote)

If QuotePos = 0 Then

EnvironName = EnvironName.Substring(1)

Else

EnvironName = EnvironName.Substring(0, QuotePos)

End If

Loop

End If

GoTo TableProcess

End If

'****

If AreaNum = 0 Then

AreaMissing = True

If LastAreaName <> AreaName Then

swMissingArea.WriteLine(Quote + AreaName + Quote + "," + SourceLabel(Source) + "," + CStr(CurRecord))

swMissingArea.Flush()

LastAreaName = AreaName

End If

ElseIf AreaNum = -1 Then

GoTo nextrec 'area not included

End If

Else

AreaNum = CLng(AreaName)

FAO = RequiredFAO

End If

'Don't bother with the rest if any id fields cannot be matched

If CountryMissing Or TaxonMissing Or AreaMissing Then

GoTo NextRec

End If

TableProcess:

If TableIn Then

'get catch for years from columns

For Year = FirstYear To LastYear

CatchStr = InValues(CurRecord, Year - FirstYear)

'Contents of Catch string

If CatchStr = " " Then

GoTo NextBit2

End If

If CatchStr = "." Then

GoTo NextBit2

End If

If CatchStr = "" Then

GoTo NextBit2

End If

If CatchStr = "-" Then

GoTo NextBit2

End If

If CatchStr.Contains("...") Then

GoTo NextBit2

End If

If CatchStr = "0 0" Then

CatchStr = "0.2"

End If

If CatchStr = "<0.5" Then

CatchStr = "0.2"

End If

If CatchStr.Contains("F") Then

CatchStr = LSet(CatchStr, CatchStr.Length - 2)

End If

Tonnes = CDbl(CatchStr)

If Tonnes > 0 Then 'if the data is presented in table and 0 tonnes is provided then this is assumed to be zero and record not written

CatchID = CatchID + 1

'modified for an extra aquaculture field Oct 19 2016

If DoSource(99) = False And DoSource(98) = False Then

strLine = CStr(CatchID) + "," + CStr(Year) + "," + CStr(CNumber) + "," + CStr(TaxonNum) + "," + CStr(AreaNum) + "," + CStr(FAO) + "," + CStr(Tonnes) + "," + CStr(Source)

Else

strLine = CStr(CatchID) + "," + CStr(Year) + "," + CStr(CNumber) + "," + CStr(EnvironName) + "," + CStr(TaxonNum) + "," + CStr(AreaNum) + "," + CStr(FAO) + "," + CStr(Tonnes) + "," + CStr(Source)

End If

'strip off quotes

Do Until strLine.Contains(Quote) = False

QuotePos = strLine.IndexOf(Quote)

If QuotePos = 0 Then

strLine = strLine.Substring(1)

Else

strLine = strLine.Substring(0, QuotePos) + strLine.Substring(QuotePos + 1)

End If

Loop

sw.WriteLine(strLine)

'write grand output

If DoSegment(Source, Year, FAO) Then

CATCHID = CATCHID + 1

swo.WriteLine(CStr(CATCHID) + "," + strLine)

End If

End If

NextBit2:

Next Year

Else

Tonnes = CDbl(InTonnes(CurRecord))

If Tonnes = 0 Then Tonnes = MinimumCatch

'data already flattened - not in table format

CatchID = CatchID + 1

strLine = CStr(CatchID) + "," + InYear(CurRecord) + "," + CStr(CNumber) + "," + CStr(TaxonNum) + "," + CStr(AreaNum) + "," + CStr(FAO) + "," + CStr(Tonnes) + "," + CStr(Source)

'strip off quotes0

Do Until strLine.Contains(Quote) = False

QuotePos = strLine.IndexOf(Quote)

If QuotePos = 0 Then

strLine = strLine.Substring(1)

Else

strLine = strLine.Substring(0, QuotePos) + strLine.Substring(QuotePos + 1)

End If

Loop

sw.WriteLine(strLine)

'write grand output

IYear = CLng(InYear(CurRecord))

If DoSegment(Source, IYear, FAO) Then

CATCHID = CATCHID + 1

swo.WriteLine(CStr(CATCHID) + "," + strLine)

End If

'extrapolation only....

'If data is to be extended in time by simple identical values

If IYear = LastYear And ExtrapolatedYear > LastYear Then

For Year = IYear + 1 To ExtrapolatedYear

CatchID = CatchID + 1

strLine = CStr(CatchID) + "," + CStr(Year) + "," + CStr(CNumber) + "," + CStr(TaxonNum) + "," + CStr(AreaNum) + "," + CStr(FAO) + "," + InTonnes(CurRecord) + "," + CStr(Source)

sw.WriteLine(strLine)

'write grand output

If DoSegment(Source, IYear, FAO) Then

CATCHID = CATCHID + 1

swo.WriteLine(CStr(CATCHID) + "," + strLine)

End If

Next

End If

End If

NextRec:

Next CurRecord

sw.Flush()

sw.Close()

NextSource:

Next Source

swo.Flush()

swo.Close()

swMissingCountry.Flush()

swMissingCountry.Close()

swMissingTaxon.Flush()

swMissingTaxon.Close()

swMissingArea.Flush()

swMissingArea.Close()

If CountryMissing Then

MsgBox("Country or Countries Missing - check file")

End If

If TaxonMissing Then

MsgBox("Taxon or Taxa Missing - check file")

End If

If AreaMissing Then

MsgBox("Area or Areas Missing - check file")

End If

Windows.Forms.Cursor.Current = Windows.Forms.Cursors.Default

Catch_Import = True

End Function

**2. Compilation**

Public Function buildCATCH2(ByRef formallo As frmAllo, ByRef CATCHparas As CATCHvariables.Globals) As Boolean

Dim ans As Windows.Forms.DialogResult

Dim strt As DateTime

Dim tsp As TimeSpan

'define min max years

frm = formallo

'CATCHparas = New CATCHvariables.Globals

Try

'########################################

' If frm.ParaMDB.Checked = True Or frm.paraCATCH3local.Checked = True Then

CATCHparas.pathA = CATCHparas.pMDB_CATCH_main

CATCHparas.pathB = "F:\Catch\Catch 2015\CATCH CATCH\CATCH2_Documentation.txt"

CATCHparas.pathC = "F:\Catch\Catch 2015\CATCH CATCH\CATCH2_Integrity_Report.txt" 'location of file for integrity report

'Else

'ans = MsgBox("Do you want to use SimMap3 databases?", MsgBoxStyle.YesNo, "Data Source Check")

'If ans = Windows.Forms.DialogResult.No Then GoTo abort

'CATCHparas.setGlobalDefaults()

'CATCHparas.pathA = "C:\My Documents\Projects\SimMap3\DataBases\CATCH.mdb"

'CATCHparas.pathB = "C:\My Documents\Projects\SimMap3\Documentation\CATCH2_Documentation.txt"

'CATCHparas.pathC = "C:\My Documents\Projects\SimMap3\Documentation\CATCH2_Integrity_Report.txt" 'location of file for integrity report

'End If

'CATCHparas.maxYear = maxyr '#############

sqlExc = New sqlCommandExecuter

sqlExc.connect(CATCHparas.pathA)

blankLine = vbCrLf & vbCrLf

frm.ProgressBarSml.Minimum = 0

frm.ProgressBarSml.Maximum = 7

frm.ProgressBarSml.Value = 0

frm.ProgressBarSml.Step = 1

frm.ProgressBarSml.Visible = True

strt = Now

frm.ProgressBarSml.PerformStep() '1

'check for nulls and or anomalies in catch table

docStr = String.Format(Now, "long date") & " CATCH DATA FOR " & CATCHparas.minYear & " TO " & CATCHparas.maxYear _

& blankLine & "Extraction/Adjustment Rules by Reg Watson [RW]" & blankLine _

& "--------------------------------------------------------------------------------------"

write2file(CATCHparas.pathB, docStr, True)

frm.StatusBarPanel1.Text = "Inserting Data sources ...."

frm.ProgressBarSml.PerformStep() '2

'CATCH2 data table already assembled

'If insertPreFormattedCatchData() = False Then Throw New System.Exception("Error with Data Sources")

'If insertDataSources() = False Then Throw New System.Exception("Error with Data Sources")

'Remove Unwanted Taxa

frm.StatusBarPanel1.Text = "Removing Unwanted Groups ...."

frm.ProgressBarSml.PerformStep() '3

If removeUnwantedGroups(CATCHparas) = False Then Throw New System.Exception("Error with Unwanted groups removal")

'============= CORRECTIONS / REASSIGNMENTS ======================================"

''CORRECT: Country

frm.StatusBarPanel1.Text = "Correcting Country ...."

frm.ProgressBarSml.PerformStep() '4

If correctCountry(CATCHparas) = False Then Throw New System.Exception("Error with Country Correction")

''MISIDENTIFICATIONS

frm.StatusBarPanel1.Text = "Correcting Misidentifications .... "

frm.ProgressBarSml.PerformStep() '5

If correctTaxonomy(CATCHparas) = False Then Throw New System.Exception("Error with Taxonomy Correction")

''WRONG AREA

frm.StatusBarPanel1.Text = "Correcting Wrong Areas .... "

frm.ProgressBarSml.PerformStep() '6

If correctArea(CATCHparas) = False Then Throw New System.Exception("Error with Area Correction")

'============= Check integrity of CATCH2 ==============================================

frm.StatusBarPanel1.Text = "Checking integrity of CATCH2 ...."

frm.ProgressBarSml.PerformStep() '8

ans = MsgBox("Check Integrity?", MsgBoxStyle.YesNo, "Warning")

If ans = vbYes Then

CheckCATCH2(CATCHparas)

End If

abort:

tsp = Now.Subtract(strt)

Return True

Catch ex As System.Exception

MsgBox("buildCATCH2 Function: " & ex.Message, MsgBoxStyle.Critical, "Fatal Error in Building CATCH2 table")

Return False

Finally

frm.StatusBarPanel1.Text = ""

frm.StatusBarPanel2.Text = ""

frm.ProgressBarSml.Visible = False

If sqlExc Is Nothing = False Then

sqlExc.close()

sqlExc = Nothing

End If

If CATCHparas Is Nothing = False Then

'CATCHparas.__dtor()

CATCHparas = Nothing

End If

MsgBox("Finished " & tsp.Minutes & " minute/s")

End Try

End Function

**3. Disaggregation**

Public Sub Disagg2(ByVal frm As frmDisagg)

Dim strSQL As String

Dim TaxStr As String

Dim rst As New DataTable

Dim rstAreasp As New DataTable

Dim rstGlobeAcc As New DataTable

Dim rstNeighbours As New DataTable

Dim CatchCurrent As Decimal

Dim CatchAssigned As Decimal

Dim i, j, L As Long

Dim CATCH2data As udtCATCH2data

Dim flgDocument As Boolean

Dim AggNum As Long = 0

Dim numRec As Long

Dim SpeciesList() As Long

Dim WHERE As String

Dim MDBerrorFlg As Boolean = False

Dim MDBerrorCount As Integer

Dim prevCnumber As Long = 0

Dim prevArea As Long = 0

Dim prevTaxa As Long = 0

Dim prevYear As Long = 0

Dim strTaxSource As String

Dim start As DateTime = Now

Dim tsp As TimeSpan

Dim flgPrintCandidates As Boolean = False 'just print candidates to a file for testing :::::::::::::::::::::::::::::

Dim flgRecordCandidates As Boolean = False 'print numbers of candidates

'weighting factors in Priority score used in sql ::::::::::::::::::::::::::::::::::::::::::::::::::::::::::::::::::::

Dim PriceFactor As Long = 1

Dim LengthFactor As Long = 1

Dim NeighbourFactor As Long = 1

Dim YearFactor As Long = 1

Dim CatchFactor As Decimal = 0.5

Dim MinFraction As Decimal = 0.001

Dim MinCatch As Decimal = 0.1

Dim NumCandidates As Long = 0

Dim MaxCandidates As Long = 50

Dim PreCandidates As Long = 0

Dim SumPriority As Decimal

Dim SecondSum As Decimal

Dim PriorityFraction() As Decimal

Dim CatchShare() As Decimal

Dim Prop As Decimal

Dim NumPossible As Long

Dim Useable() As Boolean

Dim NumUsed As Long

Dim CandLeft As Long

Dim ReadTotal As Decimal = 0

Dim UsedTotal As Decimal = 0

Dim ListValid As Boolean = False

Dim FishListSQL As String

Dim flgRecordList As Boolean = False 'Just records viable candidates into a list for future runs - can take 90hr to run!!

Dim flgUseRecodedList As Boolean = True 'use viablelist database produced by this routine earlier

Dim Quote As Char = ControlChars.Quote

Dim flgCheckRecord As Boolean = False

'modified version using neighbour's catch

'r.watson May 2009 - not sure if all catch conserved.. changed double to decimal types

'r.watson March 2009 'problem debugged with catch lost

'r.watson October 2006

Try

comProc = New Common

CATCHparas = New CATCHvariables.Globals

ReadCATCHParas(CATCHparas)

If frm.chk_datasource.Checked = True Then

'CATCHParas.setGlobalDefaults2()

CATCHParas.pathA = CATCHParas.pListFiles & "\NonDisag.csv"

CATCHParas.pathB = CATCHParas.pListFiles & "\AggDoc.csv"

If frm.chkDoAgain.Checked = True Then

CATCHParas.pathC = frm.txtDisag.Text & "\CATCH3extras.csv"

Else

CATCHParas.pathC = frm.txtDisag.Text & "\CATCH3.csv"

End If

Else

CATCHParas.setGlobalDefaults()

CATCHParas.pathA = CATCHParas.pSimMap3 & "\NonDisag.csv"

CATCHParas.pathB = CATCHParas.pSimMap3 & "\AggDoc.csv"

If frm.chkDoAgain.Checked = True Then

CATCHParas.pathC = frm.txtDisag.Text & "\CATCH3extras.csv"

Else

CATCHParas.pathC = frm.txtDisag.Text & "\CATCH3.csv"

End If

End If

If flgRecordList Then

CATCHParas.pathC = CATCHParas.pListFiles & "\ViableList.csv"

Else

If flgPrintCandidates Then CATCHParas.pathC = CATCHParas.pListFiles & "\Candidates.csv" '::::::::::::::::::::::::::::::::::::::::::::

End If

CATCHParas.maxPos = 259200

' tester = True

' If tester = True Then

' ans = MsgBox("Tester is On!", vbOKCancel)

' If ans = 2 Then Exit Sub

' End If

frm.Panel1.Text = "Preparing output files and input arrays...."

frm.Refresh()

'=========================================================================================================

'=========================== IO ==========================================================================

'Document

flgDocument = False

If flgDocument Then

If write2file(CATCHParas.pathB, "", True) = False Then GoTo abort '#77

End If

'#52 'NonDisag

If write2file(CATCHParas.pathA, "CATCHID,CatchID,TaxonKey,AreaCode,CountCode,Year", True) = False Then GoTo abort

':::::::::::::::::::::::::::::::::::::::::::::::::::::::::::::::::::::::::::::::::::::::::::::::::::::::::::::::::::::::::::

'write out what would be selected for testing purposes

If flgRecordList Then

swCATCH3 = New IO.StreamWriter(CATCHParas.pathC)

swCATCH3.WriteLine("CNumber, Area, Taxonkey, IYear, FishList")

Else

If flgPrintCandidates Then

swCATCH3 = New IO.StreamWriter(CATCHParas.pathC)

swCATCH3.WriteLine("CATCHID, CatchID, Year, Source, CATCHgrp, TaxonKey, TaxLevel, TaxonName, CommonName, SuperTargetGrp, " _

& "TargetGrpNum, ISSCAAP,ClaCode,OrdCode,FAOArea, AreaCode, CNumber, HighSeas, OwnEEZ, DWFEEZ, FAO Name, Catch, OrigTaxon, NewTaxon, PriorityFraction, Priority, NewTaxonName, NewCommonName")

Else

'#88 'CATCH3

swCATCH3 = New IO.StreamWriter(CATCHParas.pathC)

swCATCH3.WriteLine("CATCHID, CatchID, Year, Source, CATCHgrp, TaxonKey, TaxLevel, TaxonName, CommonName, SuperTargetGrp, " _

& "TargetGrpNum, ISSCAAP,ClaCode,OrdCode,FAOArea, AreaCode, CNumber, HighSeas, OwnEEZ, DWFEEZ, FAO Name, Catch, Agg, OrigTaxon")

End If

End If

':::::::::::::::::::::::::::::::::::::::::::::::::::::::::::::::::::::::::::::::::::::::::::::::::::::::::::::::::::::::::::::::::::::::::

If flgRecordCandidates Then

swCATCH4 = New IO.StreamWriter(CATCHParas.pListFiles & "\CandidateFilter.csv")

swCATCH4.WriteLine("Year, TaxonKey, TaxLevel, TaxonName, CommonName, " _

& "ISSCAAP,ClaCode,OrdCode,FAOArea, AreaCode, CNumber, HighSeas, OwnEEZ, DWFEEZ, FAO Name, Catch, PreCandidates, NumAccessible, NumFinal")

End If

'=========================================================================================================

'NOTE THAT THIS IS OPERATING ON THE ACCESS DATABASE BUT THE PREVIOUS STEP OPERATES ON THE SQLSERVER

'~~~~~~~~~~~~~~~~~~~~~~~~~~~~~~~~~~~~~~~~~~~~~~~~~~~~~~~~~~~~~~~~~~~~~~~~~~~~~~~~~~~~~~~~~~~~~~~~~~~~~~~~~~~~~

'Fill a public array of Highseas and OwnEEZ values for flags in SpDisagg procedure

rstGlobeAcc.Clear()

strSQL = "SELECT Country2.CNumber, Country2.HighSeas, Country2.OwnEEZ FROM Country2 ORDER BY Country2.CNumber;"

rstGlobeAcc = comProc.getDataTable(CATCHParas.pMDB_Disaggregation, strSQL)

ReDim GlobeAcc(999)

With rstGlobeAcc

For i = 0 To .Rows.Count - 1

GlobeAcc(.Rows(i).Item("CNumber")).CNumber = .Rows(i).Item("CNumber")

GlobeAcc(.Rows(i).Item("CNumber")).HighSeas = .Rows(i).Item("HighSeas")

GlobeAcc(.Rows(i).Item("CNumber")).OwnEEZ = .Rows(i).Item("OwnEEZ")

Next i

End With

'accRecs = New accessRecords

'If accRecs.loadAccessRecords(CATCHParas.pMDB_AccessAgreements) = False Then GoTo abort

CATCH2dt = New DataTable

CATCH2dt = comProc.getDataTable(CATCHParas.pMDB_Disaggregation, "SELECT Sum(CATCH2.Catch) AS Catch, CATCH2.year, CATCH2.TaxonKey from CATCH2 Group By CATCH2.year, CATCH2.TaxonKey")

Windows.Forms.Application.DoEvents()

'~~~~~~~~~~~~~~~~~~~~~~~~~~~~~~~~~~~~~~~~~~~~~~~~~~~~~~~~~~~~~~~~~~~~~~~~~~~~~~~~~~~~~~~~~~~~~~~~~~~~~~~~~~~~~

frm.Panel1.Text = "Getting CATCH2 data......"

frm.Refresh()

'############# READ SPECIES EEZ DATA INTO ARRAY ############################################

'First find species that occurs in AreaCode

'Find if FishEEZ has permission to fish in AreaCode

If frm.chkDoAgain.CheckState = Windows.Forms.CheckState.Checked Then 'Only do records that are matched with the DoAgain table in Disaggregation.mdb

strSQL = getCATCH2sql(True)

Else

strSQL = getCATCH2sql()

End If

'testing only - limits records used !!!!!!!!!!!!!!!!!!!!!!!!!!!!!!!!!!!!!!!!!!!!!!!!!!!!

'Where CATCH2.CNumber = 156 and CATCH2.TaxonKey <200000

'strSQL = "SELECT CATCH2.CATCHID, CATCH2.CatchID, CATCH2.Year, CATCH2.Source, TaxonNom.CATCH, CATCH2.TaxonKey, TaxonNom.TaxLevel, TaxonNom.TaxonName, TaxonNom.CommonName, TaxonNom.SuperTarget, TaxonNom.TargetGrpNum, TaxonNom.ISSCAAP, TaxonNom.ClaCode, TaxonNom.OrdCode, AreaCode.FAO AS FAOarea, CATCH2.AreaCode, CATCH2.CNumber, Country2.HighSeas, Country2.OwnEEZ, Country2.DWFEEZ, Country2.[FAO name] AS FAOname, CATCH2.Catch FROM ((CATCH2 INNER JOIN TaxonNom ON CATCH2.TaxonKey = TaxonNom.TaxonKey) INNER JOIN Country2 ON CATCH2.CNumber = Country2.CNumber) INNER JOIN AreaCode ON CATCH2.AreaCode = AreaCode.AreaCode Where CATCH2.CNumber = 156 and CATCH2.TaxonKey <200000 ORDER BY CATCH2.CNumber, CATCH2.AreaCode, CATCH2.TaxonKey, CATCH2.Year;"

'Where CATCH2.CNumber = 300

'strSQL = "SELECT CATCH2.CATCHID, CATCH2.CatchID, CATCH2.Year, CATCH2.Source, TaxonNom.CATCH, CATCH2.TaxonKey, TaxonNom.TaxLevel, TaxonNom.TaxonName, TaxonNom.CommonName, TaxonNom.SuperTarget, TaxonNom.TargetGrpNum, TaxonNom.ISSCAAP, TaxonNom.ClaCode, TaxonNom.OrdCode, AreaCode.FAO AS FAOarea, CATCH2.AreaCode, CATCH2.CNumber, Country2.HighSeas, Country2.OwnEEZ, Country2.DWFEEZ, Country2.[FAO name] AS FAOname, CATCH2.Catch FROM ((CATCH2 INNER JOIN TaxonNom ON CATCH2.TaxonKey = TaxonNom.TaxonKey) INNER JOIN Country2 ON CATCH2.CNumber = Country2.CNumber) INNER JOIN AreaCode ON CATCH2.AreaCode = AreaCode.AreaCode Where CATCH2.CNumber = 300 ORDER BY CATCH2.CNumber, CATCH2.AreaCode, CATCH2.TaxonKey, CATCH2.Year;"

'strSQL = "SELECT CATCH2.CATCHID, CATCH2.CatchID, CATCH2.Year, CATCH2.Source, TaxonNom.CATCH, CATCH2.TaxonKey, TaxonNom.TaxLevel, TaxonNom.TaxonName, TaxonNom.CommonName, TaxonNom.SuperTarget, TaxonNom.TargetGrpNum, TaxonNom.ISSCAAP, TaxonNom.ClaCode, TaxonNom.OrdCode, AreaCode.FAO AS FAOarea, CATCH2.AreaCode, CATCH2.CNumber, Country2.HighSeas, Country2.OwnEEZ, Country2.DWFEEZ, Country2.[FAO name] AS FAOname, CATCH2.Catch FROM ((CATCH2 INNER JOIN TaxonNom ON CATCH2.TaxonKey = TaxonNom.TaxonKey) INNER JOIN Country2 ON CATCH2.CNumber = Country2.CNumber) INNER JOIN AreaCode ON CATCH2.AreaCode = AreaCode.AreaCode Where CATCH2.Year = 1950 ORDER BY CATCH2.CNumber, CATCH2.AreaCode, CATCH2.TaxonKey, CATCH2.Year;"

'@@@@@@@@@@@@@@@@@@@@@@@@@@@@@@@@@@@@@@@@@@@@@@@@@@@@@@@@@@@@@@@@@@@@@@@@@@@@@@@@@@@@

rst = comProc.getDataTable(CATCHParas.pMDB_Disaggregation, strSQL) '.Open(strSQL, curconn, ADODB.CursorTypeEnum.adOpenStatic, ADODB.LockTypeEnum.adLockReadOnly)

Windows.Forms.Application.DoEvents()

frm.Panel1.Text = "Starting disaggregation......"

frm.ProgressBar1.Minimum = 0

frm.ProgressBar1.Maximum = rst.Rows.Count

frm.ProgressBar1.Value = 0

frm.ProgressBar1.Step = 1

frm.ProgressBar1.Visible = True

frm.Refresh()

numRec = rst.Rows.Count

flgCheckRecord = True

'(((((((((((((((((((((((((((((((((((((((((((((((((((((((((((((((((((((((((((((((((((((((((((((((((((((((((((((((((((((((((((((((((((((

For i = 0 To numRec - 1

If flgCheckRecord = False Then Stop

flgCheckRecord = False

CATCH2data = fillRecord(rst.Rows(i))

'tests **********************************************

'If i < 455 Then GoTo MoveNextDiss

'If i = 2072 Then Stop

'If CATCH2data.CNumber > 12 Then Stop

'#### TESTER ###########################################

' If tester = True Then

' If ICountCode <> 999 Then

' GoTo MoveNextDiss

' Else

' tester = False

' End If

' End If

CatchCurrent = CATCH2data.Tonnes 'none should be lost !!!!!!!!!!!!!!!!!!!!!!!!!!!!!!!!!!!!!!!!!!!!!!!!!

ReadTotal = ReadTotal + CatchCurrent

'rw March 09 added a limit of .1 tonnes otherwise do not try to disagg

If CATCH2data.TaxLevel < 4 And CATCH2data.CNumber > 0 And CATCH2data.Tonnes >= MinCatch Then

'aggregated record found - get records from AreaSp Table to find likely candidates

'each as Prob code from 1=low likelihood to 5 very likely, and a relative abundance value assigned based on

'prop of distribution of taxon in the defined area as well as a factor (5^TL) relating abundance across TL levels

tsp = Now.Subtract(start)

frm.Panel1.Text = "Doing " & i & " of " & numRec & " : " & Int(tsp.TotalHours) & " hrs " & tsp.Minutes & " min"

frm.Panel2.Text = CATCH2data.FAOName & " (" & Trim(Str(CATCH2data.CNumber)) & ")"

'TotalCatch = 0

AggNum = AggNum + 1

TaxStr = getTaxalevelstr(CATCH2data) 'get appropriate sql taxon clause

If (prevCnumber = CATCH2data.CNumber) And (prevArea = CATCH2data.NewArea) And (prevTaxa = CATCH2data.TaxonKey) And (prevYear = CATCH2data.IYear) Then

'use the same list of viable candidates

Else

If flgUseRecodedList = False Then

'############ GET DISAGG SPECIES LIST ####################################################

If (prevCnumber = CATCH2data.CNumber) And (prevArea = CATCH2data.NewArea) And (prevTaxa = CATCH2data.TaxonKey) Then

'possibly use the previous specieslist

prevCnumber = CATCH2data.CNumber

prevArea = CATCH2data.NewArea

prevYear = CATCH2data.IYear

prevTaxa = CATCH2data.TaxonKey

SpeciesList = GetDisagAccess(CATCH2data.IYear, CATCH2data.NewArea, CATCH2data.CNumber, TaxStr, True)

prevSplist = SpeciesList

Else

prevCnumber = CATCH2data.CNumber

prevArea = CATCH2data.NewArea

prevTaxa = CATCH2data.TaxonKey

prevYear = CATCH2data.IYear

SpeciesList = GetDisagAccess(CATCH2data.IYear, CATCH2data.NewArea, CATCH2data.CNumber, TaxStr)

prevSplist = SpeciesList

End If

Else

'get the list of viable taxa in the prepared table Viablelist

strSQL = "SELECT ViableList.FishList FROM(ViableList) "

strSQL = strSQL + "where (((ViableList.CNumber)=" + CStr(CATCH2data.CNumber) + ") AND ((ViableList.Area)=" + CStr(CATCH2data.NewArea) + ") "

strSQL = strSQL + "AND ((ViableList.Taxonkey)=" + CStr(CATCH2data.TaxonKey) + ") AND ((ViableList.IYear)=" + CStr(CATCH2data.IYear) + "));"

rstAreasp.Clear()

MDBerrorCount = 0

'get the list of dissag species from the database

rstAreasp = comProc.getDataTable(CATCHParas.pMDB_Disaggregation, strSQL)

'PreCandidates = rstAreasp.Rows.Count - 1

'If PreCandidates < 0 Then GoTo LeaveAggreg 'no viable (no distributions accessable by country in year that fit

'ReDim SpeciesList(PreCandidates - 1)

PreCandidates = rstAreasp.Rows.Count

If PreCandidates < 1 Then GoTo LeaveAggreg 'no viable (no distributions accessable by country in year that fit

ReDim SpeciesList(PreCandidates - 1)

For j = 0 To PreCandidates - 1

SpeciesList(j) = rstAreasp.Rows(j).Item("FishList")

Next j

End If

'Check if no species returned and if so write to file then jump to end of loop to write original record

If UBound(SpeciesList) = 0 Then

'52

If write2file(CATCHParas.pathA, CATCH2data.CATCHID & "," & CATCH2data.CatchID & "," & CATCH2data.TaxonKey & "," & CATCH2data.NewArea & "," & CATCH2data.CNumber & "," & CATCH2data.IYear) = False Then GoTo abort

GoTo LeaveAggreg

End If

FishListSQL = ""

For j = 0 To UBound(SpeciesList) - 1

FishListSQL = FishListSQL + CStr(SpeciesList(j)) + ","

Next j

FishListSQL = FishListSQL + CStr(SpeciesList(UBound(SpeciesList)))

If flgRecordList Then

For j = 0 To UBound(SpeciesList)

swCATCH3.WriteLine(CATCH2data.CNumber & "," & CATCH2data.NewArea & "," & CATCH2data.TaxonKey & "," & CATCH2data.IYear & "," & CStr(SpeciesList(j)))

Next j

'swCATCH3.WriteLine(CATCH2data.CNumber & "," & CATCH2data.NewArea & "," & CATCH2data.TaxonKey & "," & CATCH2data.IYear & "," & Quote & FishListSQL & Quote)

End If

End If

Windows.Forms.Application.DoEvents()

If flgRecordList Then GoTo MoveNextDiss

'GoTo MoveNextDiss 'Tester to stop writing

'===============================================================================================================

'CHANGE THIS TO READ IN THE CANDIDATE SPECIES FROM NEIGHBOURS ::::::::::::::::::::::::::::::::::::::::::::::::::::::::

If CATCH2data.TaxLevel = 1 Then

Select Case CATCH2data.ISSCAAP

Case 39 ' Marine fishes not identified (39) = 31-38 incl

strTaxSource = " DisAggCand.ISSCAAP) IN(31,32,33,34,35,36,37,38) "

Case 25 ' Misc Diadromous fishes (25) = 23 and 24

strTaxSource = " DisAggCand.ISSCAAP) IN(23,24) "

Case 47 ' Misc Marine Crustacean (47) = 42-46 incl

strTaxSource = " DisAggCand.ISSCAAP) IN(42,43,44,45,46) "

Case 58 ' Misc Marine Mollusc (58) = 52-57 incl

strTaxSource = " DisAggCand.ISSCAAP) IN(52,53,54,55,56,57) "

Case Else 'ONLY USE THE ISSCAAP

strTaxSource = "DisAggCand.ISSCAAP)=" + CStr(CATCH2data.ISSCAAP) + " "

End Select

ElseIf CATCH2data.TaxLevel = 2 Then

strTaxSource = "DisAggCand.ClaCode)=" + CStr(CATCH2data.ClaCode)

Else

strTaxSource = "DisAggCand.OrdCode)=" + CStr(CATCH2data.OrdCode)

End If

'PRETEST - get the number of candidates before Adrians test to see if they are accessible - then you will have to do it again with Adrian's filter

'***************************************************************************************************

If flgRecordCandidates And flgPrintCandidates = False Then

strSQL = "SELECT DisAggCand.TaxonKey, TaxonNom.TaxonName, TaxonNom.CommonName, TaxonNom.TaxLevel, TaxonNom.CATCH, TaxonNom.SuperTarget, TaxonNom.TargetGrpNum, TaxonNom.ISSCAAP, TaxonNom.ClaCode, TaxonNom.OrdCode "

strSQL = strSQL + "FROM (Neighbours INNER JOIN DisAggCand ON (Neighbours.InfNumber = DisAggCand.CNumber) AND "

strSQL = strSQL + "(Neighbours.FAO = DisAggCand.FAOarea)) INNER JOIN TaxonNom ON DisAggCand.TaxonKey = TaxonNom.TaxonKey "

strSQL = strSQL + "WHERE (((" + strTaxSource + ") And ((Neighbours.CNUMBER) =" + CStr(CATCH2data.CNumber) + ")) "

strSQL = strSQL + "GROUP BY DisAggCand.TaxonKey, TaxonNom.TaxonName, TaxonNom.CommonName, TaxonNom.TaxLevel, TaxonNom.CATCH, TaxonNom.SuperTarget, TaxonNom.TargetGrpNum, TaxonNom.ISSCAAP, TaxonNom.ClaCode, TaxonNom.OrdCode "

rstAreasp.Clear()

MDBerrorCount = 0

'get the list of dissag species from the database

rstAreasp = comProc.getDataTable(CATCHParas.pMDB_Disaggregation, strSQL)

If rstAreasp Is Nothing = True Then

'MDBerror:

Throw New System.Exception("AreaSp database error")

End If

PreCandidates = rstAreasp.Rows.Count - 1

End If

'*********************************************************************************************************

strSQL = "SELECT DisAggCand.TaxonKey, TaxonNom.TaxonName, TaxonNom.CommonName, TaxonNom.TaxLevel, TaxonNom.CATCH, TaxonNom.SuperTarget, TaxonNom.TargetGrpNum, TaxonNom.ISSCAAP, TaxonNom.ClaCode, TaxonNom.OrdCode, "

strSQL = strSQL + "Sum((" + CStr(PriceFactor) + "/[disaggcand].[realprice])*(" + CStr(LengthFactor)

strSQL = strSQL + "/Log([disaggcand].[slmax]))*(" + CStr(NeighbourFactor) + "* [neighbours].[influence]) * "

strSQL = strSQL + "(" + CStr(CatchFactor) + " * Log([disaggcand].[catch]))*(" + CStr(YearFactor) + "/(Abs([disaggcand].[year]-" + CStr(CATCH2data.IYear) + ")+0.5))) AS Priority "

strSQL = strSQL + "FROM (Neighbours INNER JOIN DisAggCand ON (Neighbours.InfNumber = DisAggCand.CNumber) AND "

strSQL = strSQL + "(Neighbours.FAO = DisAggCand.FAOarea)) INNER JOIN TaxonNom ON DisAggCand.TaxonKey = TaxonNom.TaxonKey "

strSQL = strSQL + "WHERE (((" + strTaxSource + ") And ((Neighbours.CNUMBER) =" + CStr(CATCH2data.CNumber) + ")) "

If flgPrintCandidates = False Then

strSQL = strSQL + " AND DisAggCand.Taxonkey in(" + FishListSQL + ") "

End If

strSQL = strSQL + "GROUP BY DisAggCand.TaxonKey, TaxonNom.TaxonName, TaxonNom.CommonName, TaxonNom.TaxLevel, TaxonNom.CATCH, TaxonNom.SuperTarget, TaxonNom.TargetGrpNum, TaxonNom.ISSCAAP, TaxonNom.ClaCode, TaxonNom.OrdCode "

strSQL = strSQL + "ORDER BY Sum((" + CStr(PriceFactor) + "/[disaggcand].[realprice])*(" + CStr(LengthFactor)

strSQL = strSQL + "/Log([disaggcand].[slmax]))*(" + CStr(NeighbourFactor) + "*[neighbours].[influence]) * "

strSQL = strSQL + "(" + CStr(CatchFactor) + "*Log([disaggcand].[catch]))*(" + CStr(YearFactor) + "/(Abs([disaggcand].[year]-" + CStr(CATCH2data.IYear) + ")+0.5))) DESC;"

':::::::::::::::::::::::::::::::::::::::::::::::::::::::::::::::::::::::::::::::::::::::::::::::::::::::::::::::::::::::::::::

rstAreasp.Clear()

MDBerrorCount = 0

'get the list of dissag species from the database

rstAreasp = comProc.getDataTable(CATCHParas.pMDB_Disaggregation, strSQL)

If rstAreasp Is Nothing = True Then

'MDBerror:

Throw New System.Exception("AreaSp database error")

End If

NumCandidates = rstAreasp.Rows.Count - 1

If flgRecordCandidates Then

swCATCH4.WriteLine(CATCH2data.IYear & "," & CATCH2data.TaxonKey & "," & CATCH2data.TaxLevel & "," _

& Chr(34) & CATCH2data.TaxonName & Chr(34) & "," & Chr(34) & CATCH2data.CommonName & Chr(34) & "," _

& CATCH2data.ISSCAAP & "," & CATCH2data.ClaCode & "," & CATCH2data.OrdCode & "," & CATCH2data.FAOArea & "," & CATCH2data.NewArea & "," _

& CATCH2data.CNumber & "," & CATCH2data.HighSeas & "," & CATCH2data.OwnEEZ & "," & CATCH2data.DWFEEZ & "," & CATCH2data.FAOName & "," & CATCH2data.Tonnes & "," _

& PreCandidates & "," & UBound(SpeciesList) + 1 & "," & NumCandidates) 'position zero has a candidate

End If

If NumCandidates < 1 Then

GoTo LeaveAggreg 'no intersecting results... must leave *********************************************

End If

If NumCandidates > MaxCandidates Then NumCandidates = MaxCandidates

'Get provisional total of priority values for taxon candidates

SumPriority = 0

ReDim PriorityFraction(NumCandidates - 1)

ReDim CatchShare(NumCandidates - 1)

For j = 0 To NumCandidates - 1

SumPriority = SumPriority + rstAreasp.Rows(j).Item("Priority")

PriorityFraction(j) = rstAreasp.Rows(j).Item("Priority")

Next j

If SumPriority = 0 Then 'When all candidates have a 0 priority then give them equal weight

SumPriority = NumCandidates

ReDim Useable(NumCandidates - 1)

For j = 0 To NumCandidates - 1

PriorityFraction(j) = 1 / NumCandidates

CatchShare(j) = CatchCurrent * PriorityFraction(j)

Useable(j) = True

Next j

Else

'Do we need to truncate series of candidates because there are too many small fractions?

SecondSum = 0

CandLeft = NumCandidates

ReDim Useable(NumCandidates - 1)

For j = 0 To NumCandidates - 1

Prop = PriorityFraction(j) / SumPriority

If ((Prop > MinFraction And Prop * CatchCurrent > MinCatch)) Or (CandLeft < 10) Then

SecondSum = SecondSum + PriorityFraction(j)

Useable(j) = True

Else

Useable(j) = False

CandLeft = CandLeft - 1

End If

Next j

If CandLeft > 0 Then

For j = 0 To NumCandidates - 1

If Useable(j) Then CatchShare(j) = CatchCurrent * PriorityFraction(j) / SecondSum

Next j

Else

GoTo LeaveAggreg

End If

End If

':::::::::::::::::::::::::::::::::::::::::::::::::::::::::::::::::::::::::::::::::::::::::::::::::::::::::::::::::::::::::::

'write out what would be selected for testing purposes

If flgRecordCandidates Then

Else

If flgPrintCandidates Then

For j = 0 To NumCandidates - 1

If Useable(j) Then

swCATCH3.WriteLine(CATCH2data.CATCHID & "," & CATCH2data.CatchID & "," & CATCH2data.IYear & "," & CATCH2data.ISource & "," & CATCH2data.ICATCHGrp & "," _

& CATCH2data.TaxonKey & "," & CATCH2data.TaxLevel & "," & Chr(34) & CATCH2data.TaxonName & Chr(34) & "," & Chr(34) & CATCH2data.CommonName & Chr(34) & "," & CATCH2data.ISuperTargetgrp & "," _

& CATCH2data.ITargetGrpNum & "," & CATCH2data.ISSCAAP & "," & CATCH2data.ClaCode & "," & CATCH2data.OrdCode & "," & CATCH2data.FAOArea & "," & CATCH2data.NewArea & "," _

& CATCH2data.CNumber & "," & CATCH2data.HighSeas & "," & CATCH2data.OwnEEZ & "," & CATCH2data.DWFEEZ & "," & CATCH2data.FAOName & "," & CATCH2data.Tonnes & "," _

& CATCH2data.TaxonKey & "," & rstAreasp.Rows(j).Item("Taxonkey") & "," & CatchShare(j) & "," & rstAreasp.Rows(j).Item("Priority") & "," & rstAreasp.Rows(j).Item("TaxonName") & "," & rstAreasp.Rows(j).Item("CommonName"))

End If

Next j

GoTo MoveNextDiss

Else

CatchAssigned = 0

For j = 0 To NumCandidates - 1

'Write out the disagg record to CATCH3.csv """"""""""""""""""""""""""""""""""""""""""""""""""""""""""""""""""""""""""

If Useable(j) Then

CatchAssigned = CatchAssigned + CatchShare(j)

UsedTotal = UsedTotal + CatchShare(j)

swCATCH3.WriteLine(CATCH2data.CATCHID & "," & CATCH2data.CatchID & "," & CATCH2data.IYear & "," & CATCH2data.ISource & "," & rstAreasp.Rows(j).Item("CATCH") & "," _

& rstAreasp.Rows(j).Item("Taxonkey") & "," & rstAreasp.Rows(j).Item("Taxlevel") & "," & Chr(34) & rstAreasp.Rows(j).Item("TaxonName") & Chr(34) & "," & Chr(34) & rstAreasp.Rows(j).Item("CommonName") & Chr(34) & "," & rstAreasp.Rows(j).Item("SuperTarget") & "," _

& rstAreasp.Rows(j).Item("TargetGrpNum") & "," & rstAreasp.Rows(j).Item("ISSCAAP") & "," & rstAreasp.Rows(j).Item("ClaCode") & "," & rstAreasp.Rows(j).Item("OrdCode") & "," & CATCH2data.FAOArea & "," & CATCH2data.NewArea & "," _

& CATCH2data.CNumber & "," & CATCH2data.HighSeas & "," & CATCH2data.OwnEEZ & "," & CATCH2data.DWFEEZ & "," & CATCH2data.FAOName & "," & CatchShare(j) & "," & 1 & "," _

& CATCH2data.TaxonKey)

End If

Next j

If Math.Abs(CatchCurrent - CatchAssigned) > 0.0001 Then Stop

flgCheckRecord = True

'If Math.Abs((CatchCurrent - CatchAssigned) / CatchCurrent) > 0.000001 Then Stop

End If

End If

':::::::::::::::::::::::::::::::::::::::::::::::::::::::::::::::::::::::::::::::::::::::::::::::::::::::::::::::::::::::::::::::::::::::::

Else

If flgPrintCandidates Or flgRecordList Then GoTo MoveNextDiss

LeaveAggreg:

'NOT AN AGGREGATED RECORD WRITE IT UNALTERED

'PRINT TO CATCH3

'88

swCATCH3.WriteLine(CATCH2data.CATCHID & "," & CATCH2data.CatchID & "," & CATCH2data.IYear & "," & CATCH2data.ISource & "," & CATCH2data.ICATCHGrp & "," _

& CATCH2data.TaxonKey & "," & CATCH2data.TaxLevel & "," & Chr(34) & CATCH2data.TaxonName & Chr(34) & "," & Chr(34) & CATCH2data.CommonName & Chr(34) & "," & CATCH2data.ISuperTargetgrp & "," _

& CATCH2data.ITargetGrpNum & "," & CATCH2data.ISSCAAP & "," & CATCH2data.ClaCode & "," & CATCH2data.OrdCode & "," & CATCH2data.FAOArea & "," & CATCH2data.NewArea & "," _

& CATCH2data.CNumber & "," & CATCH2data.HighSeas & "," & CATCH2data.OwnEEZ & "," & CATCH2data.DWFEEZ & "," & CATCH2data.FAOName & "," & CatchCurrent & "," & 0 & "," _

& CATCH2data.TaxonKey)

UsedTotal = UsedTotal + CatchCurrent

flgCheckRecord = True

End If

Windows.Forms.Application.DoEvents()

MoveNextDiss:

frm.ProgressBar1.PerformStep()

If i Mod 1000 = 0 Then swCATCH3.Flush() : GC.Collect()

If flgPrintCandidates = False And flgRecordList = False Then

If Math.Abs(ReadTotal - UsedTotal) > 0.0001 Then Stop

End If

If flgCheckRecord = False Then Stop

Next i

'Stop

abort:

Catch ex As System.Exception

MsgBox(ex.Message, MsgBoxStyle.Critical, "Error")

Finally

If swCATCH3 Is Nothing = False Then swCATCH3.Close()

ReDim GlobeAcc(0)

CATCH2dt.Clear()

CATCH2dt = Nothing

comProc = Nothing

rstGlobeAcc.Clear()

rstGlobeAcc = Nothing

rstAreasp.Clear()

rstAreasp = Nothing

If rst Is Nothing = False Then rst.Clear()

If rst Is Nothing = False Then rst = Nothing

frm.ProgressBar1.Visible = False

End Try

End Sub

**4. Finalizing Pre-Mapped Compilation and Auxiliary Data Preparation**

Public Function markDistant(ByVal frm As frmAllo, ByRef CATCHparas As CATCHvariables.Globals)

Dim curconn As ADODB.Connection

Dim strsql As String

Dim rstsql As ADODB.Recordset

Dim flgCATCH3 As Boolean 'just in case we start updating this on another table as well

'Mark which CATCH3 records are from countries fishing outside their FAO areas - distant

'When these are allocated the annual harvest files will not be used for non tuna ones

'Field Distant will be set to Yes

'ini variables

'Dim iniInfo As New iniFile(System.AppDomain.CurrentDomain.BaseDirectory() & "\ini\CATCH.ini")

'Dim iniSection As String = "CATCHDATA"

flgCATCH3 = True ' determines whether CATCH2 used or disaggregated CATCH3 (preferred)

'CATCHparas = New CATCHvariables.Globals

'If frm.paraCATCH3local.Checked = True Or frm.ParaMDB.Checked = True Then ''

'CATCHparas.pathA = iniInfo.GetString(iniSection, "pListFiles", CATCHparas.pListFiles) & "\CellData.csv" 'not used anymore, data straight from world table

'CATCHparas.pathB = CATCHparas.pListFiles & "\ToDoTaxa.txt" 'taxa list

'Else

'CATCHparas.setGlobalDefaults()

'End If

Windows.Forms.Cursor.Current = System.Windows.Forms.Cursors.WaitCursor

curconn = New ADODB.Connection

With curconn

.CommandTimeout = 0

.Provider = "Microsoft.Jet.OLEDB.4.0"

.CommandTimeout = 0

.ConnectionString = "data source= " & CATCHparas.pMDB_CATCH_main

.Open()

End With

frm.StatusBarPanel1.Text = "Updating Distant Field..."

'Set all Distant field to No to begin with

'Update Distant field ========================================================================

If flgCATCH3 Then

strsql = "UPDATE CATCH3 SET CATCH3.Distant = No;"

End If

rstsql = New ADODB.Recordset

With rstsql

.LockType = ADODB.LockTypeEnum.adLockOptimistic

.CursorType = ADODB.CursorTypeEnum.adOpenStatic

.CursorLocation = ADODB.CursorLocationEnum.adUseClient

.Open(strsql, curconn, , , ADODB.CommandTypeEnum.adCmdText)

End With

'Update Distant field ========================================================================

If flgCATCH3 Then

strsql = "UPDATE CATCH3 INNER JOIN Country2 ON "

strsql = strsql + "CATCH3.CNumber = Country2.CNumber SET CATCH3.Distant = Yes "

strsql = strsql + "WHERE ((CStr([CATCH3].[faoarea])<>[country2].[fao] And "

strsql = strsql + "InStr([country2].[fao],CStr([CATCH3].[faoarea]))=" & Chr(34) & +"0" & Chr(34) & "));"

End If

rstsql = New ADODB.Recordset

With rstsql

.LockType = ADODB.LockTypeEnum.adLockOptimistic

.CursorType = ADODB.CursorTypeEnum.adOpenStatic

.CursorLocation = ADODB.CursorLocationEnum.adUseClient

.Open(strsql, curconn, , , ADODB.CommandTypeEnum.adCmdText)

End With

frm.StatusBarPanel1.Text = "Finished"

Windows.Forms.Cursor.Current = Windows.Forms.Cursors.Default

End Function

Public Function FillFAOHoles(ByVal formAllo As frmAllo, ByRef CATCHparas As CATCHvariables.Globals) As Boolean

'function reallocates or prorates catch across FAO boundaries to fill in 'holes'

'in years where EEZ spanning more than one FAO area had no reported catch for some of the parts

'to constrain the change of catch to FAO areas were the species has a distn you need

'to read in TaxonFAOProp (prepared by another function)

Dim strsql As String = ""

Dim AreaPath As String

Dim RootName As String

Dim LogID As String

Dim CurConn As ADODB.Connection

Dim rstsql As ADODB.Recordset

Dim TabName As String = ""

Dim CNum As Long = 0

Dim CNumCount As Long = 0

Dim EEZ As Long = 0

Dim IYear As Long = 0

Dim rstsql2 As ADODB.Recordset

Dim rstsqlA As ADODB.Recordset

Dim rstsql3 As ADODB.Recordset

Dim rstsqlTax As ADODB.Recordset

Dim rstsqlTax2 As ADODB.Recordset

Dim rstsqlAppend As ADODB.Recordset

Dim FAOCount As Long = 0

Dim Watertotal As Decimal = 0

Dim FAO() As Long

Dim FAOUsed() As Boolean

Dim FAOCountUseful() As Long

Dim FAOArea() As Decimal

Dim FAOI As Long = 0

Dim CatchCount As Long = 0

Dim flgProblemEEZ As Boolean = False

Dim NoCatch() As Boolean

Dim BadFAOCount As Long = 0

Dim GoodFAOCount As Long = 0

Dim BadArea As Decimal = 0

Dim GoodArea As Decimal = 0

Dim TaxCount As Long = 0

Dim Taxon() As Long

Dim TaxonCatch() As Decimal

Dim Tax As Long = 0

Dim CatchRecCount As Long = 0

Dim Rec As Long = 0

Dim OrigCatch As Decimal = 0

Dim SharedCatch As Decimal = 0

Dim LeftCatch As Decimal = 0

Dim CatchtoAdd As Decimal = 0

Dim CatchtoKeep As Decimal = 0

Dim TotalAdded As Decimal = 0

Dim TotalLeft As Decimal = 0

Dim OutputFile As String

Dim OldArea As Long = 0

Dim NewArea As Long = 0

Dim CurrentFAO As Long = 0

Dim PropofUnfilled As Decimal = 0

Dim flgTest As Boolean

Dim Line As String = ""

Dim flgFirst As Boolean = True

Dim Prop As Decimal = 0

Dim FAOProp As Decimal = 0

Dim GoodFAOProp As Decimal = 0

Dim BadFAOProp As Decimal = 0

Dim TotalFAOProp As Decimal = 0

Dim CatchNow As Decimal = 0

Dim AmountLost As Decimal = 0

Dim TotalLost As Decimal = 0

Dim Agg As Long = 0

Dim UsefulRecords As Boolean = False

Dim CountAreas As Long = 0

Dim TotalofBad As Decimal = 0

Dim ExcludedCountries As String = ""

Dim SFileName As String = ""

Dim TotalProp As Double = 0

Dim I As Long = 0

Dim FAOTotal() As Decimal

Dim IFAO As Long = 0

Dim EEZCells() As Boolean

Dim ExcludedFAO As String = ""

Dim CompName As String

flgTest = False

'EXCLUDED COUNTRIES... THOUGH THEY HAVE AN EEZ FAO AREA WITH NO CATCH DO NOTHING

'FOR THE US 840 DO NOT INCLUDE ADJUSTMENTS TO 61 AS THIS IS AREADY COVERED IN ALASKAN CATCH 841

'AS THIS IS THE ONLY 840 FAO THAT HAS A PROBLEM THEN DO NOT ADJUST AMERICAN 840 CATCH

'AUSTRALAN CATCH IN THE SUBPOLAR (WHERE THE PROGRAM WOULD MOVE CATCH TO) IS CAREFULLY REPORTED SO EXCLUDE AUSTRALIA

ExcludedCountries = "840,36"

' ****** LEAVE MED AND ARCTIC OUT OF FILLING PROCESS AS THESE TOO ISOLATED

'also leave out sub-antarctic areas as catch here is carefully designated already

ExcludedFAO = "18,37,58,88,48"

'ini variables

' Dim iniInfo As New iniFile(System.AppDomain.CurrentDomain.BaseDirectory() & "\ini\CATCH.ini")

' Dim iniSection As String = "CATCHDATA"

frm = formAllo

frm.Noticefrm(1, "Preparing to Fill Holes...")

Windows.Forms.Cursor.Current = Windows.Forms.Cursors.WaitCursor

ComProc = New Common

CompName = ComProc.GetComputerName.ToUpper

'If frm.paraCATCH3local.Checked = True Or frm.ParaMDB.Checked = True Then

'CATCHparas = New CATCHvariables.Globals

'CATCHparas.setGlobalDefaults2()

AreaPath = CATCHparas.pGridFiles_AreaAccessC & "\"

SpeciesPath = CATCHparas.pGridFiles_SpDistribution & "\"

'CATCHparas.pathA = iniInfo.GetString(iniSection, "pListFiles", CATCHparas.pListFiles) & "\CellData.csv" 'not used anymore, data straight from world table

'CATCHparas.pathB = iniInfo.GetString(iniSection, "pListFiles", CATCHparas.pListFiles) & "\" & "Problems_" & LogID & "_" & ComputerName & ".csv"

'CATCHparas.pathC = frm.txtProgressPath.Text & "\Progress_" & ComputerName & ".csv"

'CATCHparas.pathD = iniInfo.GetString(iniSection, "pListFiles", CATCHparas.pListFiles) & "\" & "TaxaChangeLog_" & LogID & "_" & ComputerName & ".csv"

'RootName = iniInfo.GetString(iniSection, "pResults", "E:\RWatson\RESULTS\CatchImport")

If flgAllowStatic Then

ProbAnnualFile = CATCHparas.pListFiles & "\" & "Problems_" & LogID & "_" & CompName & "_Annuals.csv"

writeOutputData(ProbAnnualFile, "Year,Source,CATCHID,AID,TargetGrpNum,TaxonName, CommonName, TaxonKey,CountryName,CountryCode,Area, SpCount,CoCount,ACount,Catch,FirstFlag,FirstName,Comment,Agg,OrigTaxon,AreaSp,AreaAccess,AccSpec,UseYear", False)

End If

'Else

'Exit Function

'End If

'================================================== SETUP =======================================

If Read_CellData(CATCHparas.pMDB_OceanSq) = False Then Throw New SystemException("Error getting Cell Data")

'SET UP CONNECTION TO LOCAL ACCESS CATCH DATABASE

'make the query from the catch database

CurConn = New ADODB.Connection

With CurConn

.CommandTimeout = 0

.Provider = "Microsoft.Jet.OLEDB.4.0"

.CommandTimeout = 0

.ConnectionString = "data source= " & CATCHparas.pMDB_CATCH_main

.Open()

End With

'get an index for each species taxon that will have an annual distribution (to reduce memory needs)

SpList.Clear()

strsql = "SELECT CATCH3.TaxonKey FROM CATCH3 GROUP BY CATCH3.TaxonKey ORDER BY CATCH3.TaxonKey;"

rstsql = New ADODB.Recordset

rstsql.Open(strsql, CurConn)

SpIndex = 0

Do Until rstsql.EOF

SpIndex = SpIndex + 1

Tax = rstsql.Fields!Taxonkey.Value

SpList(Tax) = SpIndex

rstsql.MoveNext()

Loop

'this is if you do not want to read it below for each country for each taxa ==================

'get the proportion of each taxonkey in each FAO area

' flgFirst = True

'Dim sr2 As New IO.StreamReader("C:\TaxonFAOProp.csv")

'Dim TaxFAO(2500, 99) As Double

'Do

' Line = sr2.ReadLine()

' If Line Is Nothing Then

' Exit Do

' Else

' If flgFirst = False Then

'Tax = Line.Substring(0, 6)

' FAOI = Line.Substring(7, 2)

' Prop = Line.Substring(10)

' TaxFAO(SpList(Tax), FAOI) = Prop

' Else

' flgFirst = False

'End If

'End If

'Loop Until Line Is Nothing

'sr2.Close()

'sr2 = Nothing

'PREPARE OUTPUT FILE

OutputFile = CATCHparas.pListFiles + "\HoleFill.csv"

sw = New IO.StreamWriter(OutputFile)

sw.WriteLine("State,ID,CatchID,CATCHID,Year,CNumber,Taxonkey,Agg,FAOArea,AreaCode,Catch,Shared,Now,Transaction")

'PREPARE TABLE OF EEZ CLAIMS AND AREAS FROM CURRENT WORLDEEZ TABLE

'try and delete previous temp table

TabName = "TempCountEEZ"

'drop FleetEndurance if it exists before remaking

Try

strsql = "drop TABLE " + TabName

rstsql = New ADODB.Recordset

rstsql.Open(strsql, CurConn)

Catch ex As System.Exception

End Try

'get areas and FAOs for EEZ

strsql = "SELECT WorldEEZ.CNumber, world.FAO, Sum(WorldEEZ.Area) AS ClaimArea INTO TempCountEEZ "

strsql = strsql + "FROM WorldEEZ INNER JOIN world ON WorldEEZ.Seq = world.Seq "

strsql = strsql + "GROUP BY WorldEEZ.CNumber, world.FAO "

strsql = strsql + "HAVING (WorldEEZ.CNumber>0) and (WorldEEZ.CNumber not in(" + ExcludedCountries + ")) "

strsql = strsql + "and (World.FAO not in(" + ExcludedFAO + ")); "

'>>>>>>>>>>>>>>>>>>>>>>>>>>>>>>>>>>>>>>>>>>>>>>>>>>>>>>>>>>>>>>>>>>>>>>>>>>>>>>>>>>>>>>>>>>>>>>>>>>>>>>>>>>>>>>>>>>>>>>>>>>>>>>>>>>>>>>>>>>>>>>>>>>>>>>>>>>>>>>>>>>>>>>>>

'CHECK FOR ANUM OF OTHERS TO SEE IF SIMILAR THINGS HAPPEN WITH OTHER COUNTRIES

'PRORATE WITH THE AREA OF COUNTRY'S CLAIM IN EACH FAO AREA

'SEE HOLEFILL SQL

rstsql = New ADODB.Recordset

With rstsql

.LockType = ADODB.LockTypeEnum.adLockOptimistic

.CursorType = ADODB.CursorTypeEnum.adOpenStatic

.CursorLocation = ADODB.CursorLocationEnum.adUseClient

.Open(strsql, CurConn, , , ADODB.CommandTypeEnum.adCmdText)

End With

'FROM EXISTING EEZ COUNTRIES SELECT THOSE WITH MORE THAN ONE FAO AREA

'find those with more than one FAO

strsql = "SELECT TempCountEEZ.CNumber, Count(TempCountEEZ.FAO) AS CountOfFAO "

strsql = strsql + "FROM TempCountEEZ "

strsql = strsql + "GROUP BY TempCountEEZ.CNumber "

strsql = strsql + "HAVING (((Count(TempCountEEZ.FAO))>1));"

rstsql = New ADODB.Recordset

With rstsql

.LockType = ADODB.LockTypeEnum.adLockOptimistic

.CursorType = ADODB.CursorTypeEnum.adOpenStatic

.CursorLocation = ADODB.CursorLocationEnum.adUseClient

.Open(strsql, CurConn, , , ADODB.CommandTypeEnum.adCmdText)

End With

'OPERATE ON THOSE MULTI-FAO EEZ COUNTRIES

'use this list to operate on - EEZs with more than one FAO area

CNumCount = rstsql.RecordCount

For CNum = 1 To CNumCount

EEZ = rstsql.Fields("CNumber").Value 'CNUMBER OF THE CURRENT EEZ COUNTRY

'GET LIST OF FAO AREAS FOR CURRENT EEZ COUNTRY

'find out how many FAO areas and areas for this country's EEZ

strsql = "SELECT TempCountEEZ.FAO, TempCountEEZ.ClaimArea "

strsql = strsql + "FROM TempCountEEZ "

strsql = strsql + "WHERE(((TempCountEEZ.CNumber) = " + CStr(EEZ) + ")) "

strsql = strsql + "ORDER BY TempCountEEZ.FAO;"

rstsqlA = New ADODB.Recordset

With rstsqlA

.LockType = ADODB.LockTypeEnum.adLockOptimistic

.CursorType = ADODB.CursorTypeEnum.adOpenStatic

.CursorLocation = ADODB.CursorLocationEnum.adUseClient

.Open(strsql, CurConn, , , ADODB.CommandTypeEnum.adCmdText)

End With

FAOCount = rstsqlA.RecordCount

frm.Noticefrm(1, "Checking EEZ " + CStr(EEZ) + " with " + CStr(FAOCount) + " FAO Areas...")

'For this EEZ mark the cells included ==============================================

ReDim EEZCells(MaxPos)

strsql = "Select [Seq] FROM [Translation] WHERE CNumber = " + CStr(EEZ) + " GROUP BY [Seq]"

rstsql2 = New ADODB.Recordset

With rstsql2

.LockType = ADODB.LockTypeEnum.adLockOptimistic

.CursorType = ADODB.CursorTypeEnum.adOpenStatic

.CursorLocation = ADODB.CursorLocationEnum.adUseClient

.Open(strsql, CurConn, , , ADODB.CommandTypeEnum.adCmdText)

End With

Do Until rstsql2.EOF

EEZCells(rstsql2.Fields!Seq.Value) = True

rstsql2.MoveNext()

Loop

'================================================================================================================

'make a list of FAO areas, their water area and prop of total water area

Watertotal = 0

ReDim FAO(FAOCount)

ReDim FAOUsed(FAOCount)

ReDim FAOArea(FAOCount)

ReDim NoCatch(FAOCount)

ReDim FAOCountUseful(FAOCount)

'FOR EACH FAO AREA FOR CURRENT EEZ COUNTRY

For FAOI = 1 To FAOCount

NoCatch(FAOI) = False

FAO(FAOI) = rstsqlA.Fields("FAO").Value

FAOArea(FAOI) = rstsqlA.Fields("ClaimArea").Value

Watertotal = Watertotal + FAOArea(FAOI)

rstsqlA.MoveNext()

Next FAOI

For Agg = 0 To 1 'disagg records or not

'FOR EACH YEAR (for the country)

For IYear = CATCHparas.minYear To CATCHparas.maxYear '########################################################################

'is there any catch reported by the country with the EEZ in that year (regardless of FAO areas)?

'if no catch in at all then you must ignore (cannot be fixed here - nothing to prorate)

flgProblemEEZ = False

GoodFAOCount = 0

BadFAOCount = 0

GoodArea = 0

BadArea = 0

'GET THE CATCH FOR THIS YEAR FOR WHOLE EEZ COUNTRY

strsql = "SELECT CATCH3.Year, CATCH3.CNumber, CATCH3.Catch "

strsql = strsql + "FROM CATCH3 "

strsql = strsql + "WHERE (((CATCH3.Year)=" + CStr(IYear) + ") AND ((CATCH3.CNumber)=" + CStr(EEZ) + ")) and CATCH3.Agg = " + CStr(Agg)

'strsql = strsql + "WHERE (((CATCH3.Year)=" + CStr(IYear) + ") AND ((CATCH3.CNumber)=" + CStr(EEZ) + ") AND (CATCH3.TAXONKEY = 600147) );"

rstsql2 = New ADODB.Recordset

With rstsql2

.LockType = ADODB.LockTypeEnum.adLockOptimistic

.CursorType = ADODB.CursorTypeEnum.adOpenStatic

.CursorLocation = ADODB.CursorLocationEnum.adUseClient

.Open(strsql, CurConn, , , ADODB.CommandTypeEnum.adCmdText)

End With

'IF EEZ COUNTRY HAS ANY CATCH REPORTED FOR THIS YEAR THEN PROCEED

If rstsql2.RecordCount > 0 Then

'so there is some catch reported

'is there some in all FAO areas that this country's EEZ includes?

'FOR EACH FAO AREA FOR THE CURRENT EEZ COUNTRY IN THE CURRENT YEAR GET THE CATCH

For FAOI = 1 To FAOCount

strsql = "SELECT CATCH3.Year, CATCH3.CNumber, CATCH3.Catch "

strsql = strsql + "FROM CATCH3 "

strsql = strsql + "WHERE (((CATCH3.Year)=" + CStr(IYear) + ") AND ((CATCH3.CNumber)=" + CStr(EEZ) + ") AND ((CATCH3.FAOArea)=" + CStr(FAO(FAOI)) + ")) and CATCH3.Agg = " + CStr(Agg)

rstsql3 = New ADODB.Recordset

With rstsql3

.LockType = ADODB.LockTypeEnum.adLockOptimistic

.CursorType = ADODB.CursorTypeEnum.adOpenStatic

.CursorLocation = ADODB.CursorLocationEnum.adUseClient

.Open(strsql, CurConn, , , ADODB.CommandTypeEnum.adCmdText)

End With

'IF FAO AREA IN YEAR (FOR EEZ COUNTRY) HAS NO CATCH REPORTED THAN MARK IT AS NOCATCH

'is there catch?

CatchCount = rstsql3.RecordCount

If CatchCount < 1 Then

'mark this FAO area

NoCatch(FAOI) = True

'mark that there is a problem with this EEZ in this year

flgProblemEEZ = True

BadFAOCount = BadFAOCount + 1

BadArea = BadArea + FAOArea(FAOI)

Else

NoCatch(FAOI) = False

GoodFAOCount = GoodFAOCount + 1

GoodArea = GoodArea + FAOArea(FAOI)

End If

Next FAOI

'this is dealing with the ratio of the area of the fao statistical areas that report (some catch) comparted with

'those that do not

'it is NOT about the abundance of taxa in the various areas

If Math.Abs(Watertotal - (GoodArea + BadArea)) > 0.001 Then Stop

'IF ANY FAO AREA IN THE CURRENT YEAR (FOR EEZ COUNTRY) HAD NO CATCH REPORTED (THERE IS A PROBLEM)

If flgProblemEEZ And BadArea > 0 Then

'Fix the problem in one or more FAO parts of EEZ

frm.Noticefrm(1, "Fixing EEZ " + CStr(EEZ) + " - " + CStr(IYear))

'If BadFAOCount > 2 Then Stop

'If GoodFAOCount > 2 Then Stop

'get the list of species caught in the good areas in the year by the EEZ country in total

'get the records for each species in turn and

'reduce the landings in the good areas and add records in the bad areas

'the amount of the reduction is proportional to the relative water areas

'GET THE LIST OF TAXA AND CATCHES FOR TOTAL EEZ COUNTRY REPORTING FOR THIS YEAR

'get species list and totals for EEZ Country

strsql = "SELECT CATCH3.TaxonKey, Sum(CATCH3.Catch) AS Catch "

strsql = strsql + "FROM CATCH3 "

strsql = strsql + "GROUP BY CATCH3.Year, CATCH3.CNumber, CATCH3.TaxonKey, CATCH3.AGG "

strsql = strsql + "HAVING (((CATCH3.Year)=" + CStr(IYear) + ") AND ((CATCH3.CNumber)=" + CStr(EEZ) + ")) and CATCH3.Agg = " + CStr(Agg)

rstsqlTax = New ADODB.Recordset

With rstsqlTax

.LockType = ADODB.LockTypeEnum.adLockOptimistic

.CursorType = ADODB.CursorTypeEnum.adOpenStatic

.CursorLocation = ADODB.CursorLocationEnum.adUseClient

.Open(strsql, CurConn, , , ADODB.CommandTypeEnum.adCmdText)

End With

'MAKE A LIST OF THESE TAXA

TaxCount = rstsqlTax.RecordCount

ReDim Taxon(TaxCount)

ReDim TaxonCatch(TaxCount)

For Tax = 1 To TaxCount

Taxon(Tax) = rstsqlTax.Fields("Taxonkey").Value

TaxonCatch(Tax) = rstsqlTax.Fields("Catch").Value

rstsqlTax.MoveNext()

Next Tax

'FOR EACH TAXA REPORTED BY THE EEZ COUNTRY IN THIS YEAR

For Tax = 1 To TaxCount

'what is the amount that existing catches have to be prorated

'prorate = bad area / total area

'get records for the taxon for the country and year

strsql = "SELECT CATCH3.ID, CATCH3.CatchID, CATCH3.CATCHID, CATCH3.Source, CATCH3.Year, CATCH3.CATCHgrp, "

strsql = strsql + "CATCH3.TaxLevel, CATCH3.TaxonKey, CATCH3.CommonName, CATCH3.TaxonName, CATCH3.SuperTargetGrp, CATCH3.ISSCAAP, "

strsql = strsql + "CATCH3.TargetGrpNum, CATCH3.ClaCode, CATCH3.FAOArea, CATCH3.OrdCode, CATCH3.AreaCode, CATCH3.CNumber, CATCH3.HighSeas, "

strsql = strsql + "CATCH3.DWFEEZ, CATCH3.OwnEEZ, CATCH3.Catch, CATCH3.[FAO Name], CATCH3.Agg, CATCH3.OrigTaxon, CATCH3.Distant, CATCH3.Prob, CATCH3.Added "

strsql = strsql + "FROM CATCH3 "

strsql = strsql + "WHERE (((CATCH3.Year)=" + CStr(IYear) + ") AND ((CATCH3.TaxonKey)=" + CStr(Taxon(Tax)) + ") AND ((CATCH3.CNumber)=" + CStr(EEZ) + ")) and CATCH3.Agg = " + CStr(Agg)

strsql = strsql + " AND CATCH3.FAOAREA <> 37 AND CATCH3.FAOAREA <>18 " 'NO MED OR ARCTIC ONLY SPECIES

'THERE CAN BE BOTH AN REGULAR AND A DISAGG RECORD WITH THE SAME YEAR/TAXONKEY/AREA/COUNTRY ..

'If IYear = 1950 And EEZ = 36 And Taxon(Tax) = 100038 Then Stop

rstsqlTax2 = New ADODB.Recordset

With rstsqlTax2

.LockType = ADODB.LockTypeEnum.adLockOptimistic

.CursorType = ADODB.CursorTypeEnum.adOpenStatic

.CursorLocation = ADODB.CursorLocationEnum.adUseClient

.Open(strsql, CurConn, , , ADODB.CommandTypeEnum.adCmdText)

End With

'SET UP ANOTHER RECORD SET FOR APPENDING NEW RECORDS TO - FOR THE FORGOTTEN FAO AREAS

rstsqlAppend = New ADODB.Recordset

With rstsqlAppend

.LockType = ADODB.LockTypeEnum.adLockOptimistic

.CursorType = ADODB.CursorTypeEnum.adOpenStatic

.CursorLocation = ADODB.CursorLocationEnum.adUseClient

.Open(strsql, CurConn, , , ADODB.CommandTypeEnum.adCmdText)

End With

'Go through the records - prorate them and add new records

CatchRecCount = rstsqlTax2.RecordCount

For FAOI = 1 To FAOCount

FAOUsed(FAOI) = False

FAOCountUseful(FAOI) = 0

Next FAOI

UsefulRecords = False

'FOR EACH CATCH RECORD FOR THE TAXA TAKEN BY THE EEZ COUNTRY THIS YEAR

For Rec = 1 To CatchRecCount

'Get record fields

OrigCatch = rstsqlTax2.Fields("Catch").Value

If OrigCatch < 0.000001 Or OrigCatch > 10000000 Then

'Stop 'catch weird

GoTo CheckNext

End If

'make a list of which faoareas this actually occurs in ... no point in using it in the GoodFAOProp when

'there is no catch as you have to take some away from the ones with catch

'maybe put a minimum catch

'For this year/cnumber/taxon the FAO areas have to quality to be used

CurrentFAO = rstsqlTax2.Fields("FAOArea").Value

For FAOI = 1 To FAOCount

If FAO(FAOI) = CurrentFAO And OrigCatch > 10 Then

FAOUsed(FAOI) = True

FAOCountUseful(FAOI) = FAOCountUseful(FAOI) + 1

UsefulRecords = True

End If

Next FAOI

Checknext:

rstsqlTax2.MoveNext()

Next Rec

For FAOI = 1 To FAOCount

CurrentFAO = FAO(FAOI)

If FAOCountUseful(FAOI) > 1 Then

UsefulRecords = False ' there are multiple areas within an FAO - currently too hard to handle

Exit For

End If

Next FAOI

If UsefulRecords = False Then GoTo nextTax

'FOR EACH CATCH RECORD FOR THE TAXA TAKEN BY THE EEZ COUNTRY THIS YEAR

For Rec = 1 To CatchRecCount

If Rec = 1 Then rstsqlTax2.MoveFirst()

'Get record fields

OrigCatch = rstsqlTax2.Fields("Catch").Value

If OrigCatch < 0.000001 Or OrigCatch > 10000000 Then

'Stop 'catch weird

GoTo NextRec

End If

'&&&&&&&&&&&&&&&&&&&&&&&&&&&&&&&&&&&&&&&&&&&&&&&&&&&&&&&&&&&&&&&&&&&&&&&&&&&&&&&&&&&&&&&&&&&&&&&&&&&&&&&&&&&&&&&&&&&&&&&&&&&&&&&&&&&&&&&&&&&&&&&

'get the actual prop of the distribution of the taxa in each FAO part of the country's EEZ claim

'need to read the static distn file (try annual?) and have cells of EEZ in a grid

SFileName = SpeciesPath & strAltPath & "S" & CStr(Taxon(Tax)) & ".csv"

ReDim FAOTotal(99)

If IO.File.Exists(SFileName) Then

Dim sr As New IO.StreamReader(SFileName)

I = 0

TotalProp = 0

Do

Line = sr.ReadLine()

If Line Is Nothing Then

Exit Do

Else

I = I + 1

Prop = CLng(Line)

If Prop > 0 Then

If EEZCells(I) = True Then

CurrentFAO = CellData(I).FAO

'Prop = Prop / 10 ^ 9

FAOTotal(CurrentFAO) = FAOTotal(CurrentFAO) + Prop

TotalProp = TotalProp + Prop

End If

End If

End If

Loop

Else

GoTo NextRec

End If

If TotalProp = 0 Then GoTo NextRec 'no distribution of the taxa in the EEZ claim

'########################################################################################################################

'What is the proportion of the distribution of THIS TAXON in the badareas (comparted with all area the EEZ country has)

GoodFAOProp = 0

BadFAOProp = 0

TotalFAOProp = 0

For FAOI = 1 To FAOCount

CurrentFAO = FAO(FAOI)

If FAO(FAOI) = 37 Or FAO(FAOI) = 18 Then

'Mediterranean

'Leave out

GoTo NextFAOI

End If

'FAOProp = TaxFAO(SpList(Taxon(Tax)), FAO(FAOI))

FAOProp = FAOTotal(CurrentFAO) / TotalProp

If NoCatch(FAOI) = False And FAOUsed(FAOI) = True Then 'must be a useful record to take catch away from to give to bad areas

GoodFAOProp = GoodFAOProp + FAOProp

TotalFAOProp = TotalFAOProp + FAOProp

ElseIf NoCatch(FAOI) = True Then

BadFAOProp = BadFAOProp + FAOProp

TotalFAOProp = TotalFAOProp + FAOProp

End If

nextFAOI:

Next FAOI

If TotalFAOProp < 0.0001 Or BadFAOProp < 0.0001 Then GoTo NextRec

If BadFAOProp / TotalFAOProp > 1 Then Stop

If GoodFAOProp < 0.0001 Then

'there is no good place that this country had to put this taxa so leave it alone

'possibly report it later for examination

GoTo NextRec

End If

'SharedCatch = OrigCatch * (BadArea / (BadArea + GoodArea)) 'was used based solely on ratio of whole FAO areas

SharedCatch = OrigCatch * (BadFAOProp / TotalFAOProp) 'uses ratio of the prop of global distribtion in FAO area Sept 2010

If SharedCatch < 0.000001 Or SharedCatch > 10000000 Then

Stop 'catch weird

End If

LeftCatch = OrigCatch - SharedCatch

If LeftCatch < 0 Or LeftCatch > 10000000 Then

Stop 'catch weird

End If

'ANY REASON TO ABORT (NEXTREC) BEFORE ALL FAO TRANSACTIONS WILL MEAN UNBALANCED RESULTS SO PRETEST BEFORE DOING ANY

'FIRST TIME TO MAKE SURE ALL BITS WILL MAKE IT THROUGH ############################################################################################################

'for each FAO area in the countries EEZ

For FAOI = 1 To FAOCount

CurrentFAO = FAO(FAOI)

If CurrentFAO = 37 Or CurrentFAO = 18 Then GoTo NextRec 'don't use Med OR Arctic in these calculations

OldArea = rstsqlTax2.Fields!AreaCode.Value

NewArea = 0

FAOProp = FAOTotal(CurrentFAO) / TotalProp

'if some of the distribution of this taxon is in the fao area of country eez with no catch reported in year/country

If NoCatch(FAOI) = True And FAOProp > 0 Then 'needs fixing (append records)

'add new records with the catch prorated

OldArea = rstsqlTax2.Fields!AreaCode.Value

'blocks not accepted as too far from FAO area

Select Case CurrentFAO

Case 18

If ((OldArea > 401 And OldArea < 431) Or (OldArea > 506 And OldArea < 520) Or (OldArea > 549 And OldArea < 558) Or OldArea = 483 Or (OldArea > 400000 And OldArea < 400038)) Then 'NAFO and ICES areas too far from 18

GoTo NextRec

End If

Case 34

If (OldArea > 400 And OldArea <= 425) Or (OldArea > 426 And OldArea <= 529) Or (OldArea > 530 And OldArea <= 541) Then

GoTo NextRec

End If

If (OldArea > 400000 And OldArea < 400020) Or (OldArea > 400020 And OldArea <= 400054) Or (OldArea >= 4801000 And OldArea <= 9000000) Then

GoTo NextRec

End If

If (OldArea > 4701100 And OldArea < 4705000) Or (OldArea > 4705000 And OldArea < 4799999) Then

GoTo NextRec

End If

Case 37

If (OldArea >= 3701000 And OldArea < 4101000) Then 'nothing close to Med really

GoTo NextRec

End If

Case 47

If ((OldArea >= 400 And OldArea < 700) Or (OldArea >= 7101000 And OldArea <= 8803000) Or OldArea = 4803000 Or OldArea = 4802000 Or OldArea = 4805000) Then

GoTo NextRec

End If

Case 48

If ((OldArea >= 400 And OldArea < 615) Or (OldArea >= 400000 And OldArea < 3404200) Or OldArea = 4705000 Or OldArea = 8801000 Or OldArea = 8802000) Then

GoTo NextRec

End If

Case 51

If ((OldArea >= 400 And OldArea < 726) Or (OldArea > 400000 And OldArea < 600000) Or (OldArea >= 3401000 And OldArea <= 4806000) Or (OldArea >= 584000 And OldArea <= 5804402) Or OldArea = 4701500 Or OldArea = 4701600 Or OldArea = 4702100) Then 'TOO FAR

GoTo NextRec

End If

Case 57

If ((OldArea >= 400 And OldArea < 726) Or (OldArea > 400000 And OldArea < 600000) Or (OldArea > 3101000 And OldArea < 4806000)) Then 'TOO FAR

GoTo NextRec

End If

Case 58

If (OldArea = 6031 Or OldArea = 10037 Or OldArea = 4701600 Or OldArea = 4702100 Or OldArea = 4701400 Or OldArea = 4701500 Or OldArea = 4702200 Or OldArea = 4701000 Or OldArea = 4803000 Or OldArea = 4704000) Then 'TOO FAR

GoTo NextRec

End If

Case 61

If ((OldArea >= 400 And OldArea <= 1000) Or (OldArea >= 3101000 And OldArea <= 5807000) Or (OldArea >= 8101000 And OldArea <= 8803000)) Then

GoTo NextRec

End If

Case 67

If ((OldArea > 400 And OldArea < 725) Or (OldArea > 3000000 And OldArea < 9000000) Or (OldArea > 400000 And OldArea < 600000)) Then 'not close

GoTo NextRec

End If

Case 71

If OldArea < 615 Then

GoTo NextRec

End If

Case 77

If ((OldArea >= 400 And OldArea < 726) Or (OldArea > 400000 And OldArea < 600000) Or (OldArea > 3101000 And OldArea <= 5807000)) Then 'TOO FAR

GoTo NextRec

End If

Case 81

If ((OldArea > 3400000 And OldArea < 3500000) Or (OldArea > 4800000 And OldArea < 4900000)) Then 'too far

GoTo NextRec

End If

Case 87

If ((OldArea >= 3000000 And OldArea <= 7108000) Or OldArea = 8801000) And CurrentFAO = 87 Then

GoTo NextRec

End If

End Select

'conditional use of the area record

If OldArea < 100 Then

NewArea = CurrentFAO

ElseIf (OldArea = 4701100 Or OldArea = 4701000) And CurrentFAO = 34 Then ' 47.1.1 or 47.1 -> 34.3.6

NewArea = 3403600

ElseIf OldArea = 331060 And CurrentFAO = 21 Then 'FAO 31 Bermuda EEZ -> FAO 21 Bermuda EEZ

NewArea = 321060

ElseIf (OldArea = 4801000 Or OldArea = 4802000) And CurrentFAO = 41 Then '48.1 or 48.2 -> 41.3.2

NewArea = 4103200

ElseIf OldArea = 4803000 And CurrentFAO = 41 Then '48.3 -> 41.3

NewArea = 4103000

ElseIf OldArea = 3403600 And CurrentFAO = 47 And (EEZ = 178 Or EEZ = 266) Then '34.3.6 gabon or congo republic -> 47.5

NewArea = 4705000

ElseIf OldArea = 3403600 And CurrentFAO = 47 And EEZ = 180 Then '34.3.6 dem congo republic -> 47.1.1

NewArea = 4701100

ElseIf (OldArea = 549 Or OldArea = 502 Or OldArea = 401 Or OldArea = 400038) And CurrentFAO = 18 Then 'NAFO 0 or 0A or ICES 1

NewArea = 18 ' just possible as it does touch - but much ice

ElseIf OldArea = 3703200 And CurrentFAO = 51 Then '37.3.2 Med sea ->51 (may not work)

NewArea = 51

ElseIf OldArea = 10580 Then 'N Marian 580 EEZ across both areas

NewArea = OldArea

ElseIf OldArea = 4701200 Or OldArea = 4701300 Then

GoTo NextRec 'this area is away from the boundary and specific - leave unaltered

ElseIf (OldArea = 3403500) And CurrentFAO = 47 And EEZ = 266 Then '34.3.5 gabon too far from 47

GoTo NextRec ' not adjacent

ElseIf OldArea = 5254 Or OldArea = 5256 Then

If CurrentFAO <> 61 And CurrentFAO <> 67 Then 'LME area Chukchi Sea - only 18; LME area East Bering Sea, 61 and 67

GoTo NextRec

Else

NewArea = CurrentFAO

End If

ElseIf OldArea = 5805000 And CurrentFAO = 51 Then '58.5 -> 51.7

NewArea = 5107000

ElseIf (OldArea = 5805000 Or OldArea = 5805100 Or OldArea = 5805200) Or CurrentFAO = 57 Then

NewArea = 5704000

ElseIf OldArea = 5257 And CurrentFAO <> 18 Then 'LME area Laptev sea

GoTo NextRec

ElseIf OldArea = 5258 And CurrentFAO <> 18 Then 'LME area Kara sea

GoTo NextRec

ElseIf OldArea = 318840 Then

If CurrentFAO = 61 Or CurrentFAO = 67 Then

NewArea = CurrentFAO

Else

GoTo NextRec

End If

ElseIf OldArea = 426 And CurrentFAO = 34 Then

NewArea = CurrentFAO

ElseIf OldArea = 4804000 And CurrentFAO = 47 Then

NewArea = 4704000

ElseIf OldArea = 4705000 And CurrentFAO = 34 And EEZ = 654 Then

NewArea = 3404100

ElseIf (OldArea = 8802000 Or OldArea = 8803000) And CurrentFAO = 87 Then

NewArea = 8703300

Else

'Stop

GoTo NextRec

End If

End If

Next FAOI

'============================================================================================================================================================================

'NOW FOR REAL ##########################################################################

TotalAdded = 0

TotalLeft = 0

TotalLost = 0

'FIRST PASS.. REMOVING ONLY ===================================================================================================

'for each FAO area in the countries EEZ

For FAOI = 1 To FAOCount

CurrentFAO = FAO(FAOI)

If CurrentFAO = 37 Or CurrentFAO = 18 Then GoTo NextRec 'don't use Med or Arctic in these calculations

OldArea = rstsqlTax2.Fields!AreaCode.Value

NewArea = 0

FAOProp = FAOTotal(CurrentFAO) / TotalProp

If NoCatch(FAOI) = False And FAOUsed(FAOI) = True And FAOProp > 0.00001 Then 'needs fixing (append records) >>>>>>> GOOD AREAS - REDUCE CATCH

'this is where catch must be prorated - there may be multiple "good areas"

'prorate by the amount in each of the good areas

PropofUnfilled = FAOProp / GoodFAOProp 'now based on distn proportion Sept 2010 = TaxFAO(SpList(Taxon(Tax)), FAO(FAOI)) / BadFAOProp 'now based on distn proportion Sept 2010

'If PropofUnfilled <> 1 Then Stop

AmountLost = PropofUnfilled * SharedCatch

'AmountLost = Math.Round(AmountLost, 8)

CatchNow = rstsqlTax2.Fields("Catch").Value

If NewArea = 0 Then NewArea = CurrentFAO

If CatchNow < AmountLost Then

AmountLost = CatchNow

End If

'ACCOUNTING FOR REDUCTIONS ----------------------------------------------------

If flgTest = False Then

sw.WriteLine("Reduced," + CStr(rstsqlTax2.Fields!ID.Value) + "," + CStr(rstsqlTax2.Fields!CatchID.Value) + "," + CStr(rstsqlTax2.Fields!CATCHID.Value) + "," + CStr(IYear) + "," + CStr(rstsqlTax2.Fields!CNumber.Value) + "," + CStr(rstsqlTax2.Fields!Taxonkey.Value) + "," + CStr(rstsqlTax2.Fields!Agg.Value) + "," + CStr(CurrentFAO) + "," + CStr(NewArea) + "," + CStr(Math.Round(OrigCatch, 8)) + "," + CStr(Math.Round(SharedCatch, 8)) + "," + CStr(Math.Round(CatchNow, 8)) + "," + CStr(-AmountLost))

'===================================================================================

If CatchNow - AmountLost > 0 Then

rstsqlTax2.Fields("Catch").Value = CatchNow - AmountLost '3############################################### CATCH REMOVED HERE

End If

rstsqlTax2.Fields("Added").Value = -AmountLost

'=====================================================================================

Else

'Stop

End If

TotalLost = TotalLost + AmountLost

End If

Next FAOI

'THIS 2ND PASS IS FOR ADDING ONLY

'NOW FOR REAL ##########################################################################

TotalAdded = 0

TotalofBad = 0

'for each FAO area in the countries EEZ

For FAOI = 1 To FAOCount

CurrentFAO = FAO(FAOI)

If CurrentFAO = 37 Or CurrentFAO = 18 Then GoTo NextRec 'don't use Med or Arctic in these calculations

OldArea = rstsqlTax2.Fields!AreaCode.Value

NewArea = 0

FAOProp = FAOTotal(CurrentFAO) / TotalProp

'if some of the distribution of this taxon is in the fao area of country eez with no catch reported in year/country

If NoCatch(FAOI) = True And FAOProp > 0 Then 'needs fixing (append records) >>>>>>>>>>>>>>>>>>>>>>>>> BAD AREAS - HAVE TO ADD CATCH

'add new records with the catch prorated

OldArea = rstsqlTax2.Fields!AreaCode.Value

'blocks not accepted as too far from FAO area

Select Case CurrentFAO

Case 18

If ((OldArea > 401 And OldArea < 431) Or (OldArea > 506 And OldArea < 520) Or (OldArea > 549 And OldArea < 558) Or OldArea = 483 Or (OldArea > 400000 And OldArea < 400038)) Then 'NAFO and ICES areas too far from 18

GoTo NextRec

End If

Case 34

If (OldArea > 400 And OldArea <= 425) Or (OldArea > 426 And OldArea <= 529) Or (OldArea > 530 And OldArea <= 541) Then

GoTo NextRec

End If

If (OldArea > 400000 And OldArea < 400020) Or (OldArea > 400020 And OldArea <= 400054) Or (OldArea >= 4801000 And OldArea <= 9000000) Then

GoTo NextRec

End If

If (OldArea > 4701100 And OldArea < 4705000) Or (OldArea > 4705000 And OldArea < 4799999) Then

GoTo NextRec

End If

Case 37

If (OldArea >= 3701000 And OldArea < 4101000) Then 'nothing close to Med really

GoTo NextRec

End If

Case 47

If ((OldArea >= 400 And OldArea < 700) Or (OldArea >= 7101000 And OldArea <= 8803000) Or OldArea = 4803000 Or OldArea = 4802000 Or OldArea = 4805000) Then

GoTo NextRec

End If

Case 48

If ((OldArea >= 400 And OldArea < 615) Or (OldArea >= 400000 And OldArea < 3404200) Or OldArea = 4705000 Or OldArea = 8801000 Or OldArea = 8802000) Then

GoTo NextRec

End If

Case 51

If ((OldArea >= 400 And OldArea < 726) Or (OldArea > 400000 And OldArea < 600000) Or (OldArea >= 3401000 And OldArea <= 4806000) Or (OldArea >= 584000 And OldArea <= 5804402) Or OldArea = 4701500 Or OldArea = 4701600 Or OldArea = 4702100) Then 'TOO FAR

GoTo NextRec

End If

Case 57

If ((OldArea >= 400 And OldArea < 726) Or (OldArea > 400000 And OldArea < 600000) Or (OldArea > 3101000 And OldArea < 4806000)) Then 'TOO FAR

GoTo NextRec

End If

Case 58

If (OldArea = 6031 Or OldArea = 10037 Or OldArea = 4701600 Or OldArea = 4702100 Or OldArea = 4701400 Or OldArea = 4701500 Or OldArea = 4702200 Or OldArea = 4701000 Or OldArea = 4803000 Or OldArea = 4704000) Then 'TOO FAR

GoTo NextRec

End If

Case 61

If ((OldArea >= 400 And OldArea <= 1000) Or (OldArea >= 3101000 And OldArea <= 5807000) Or (OldArea >= 8101000 And OldArea <= 8803000)) Then

GoTo NextRec

End If

Case 67

If ((OldArea > 400 And OldArea < 725) Or (OldArea > 3000000 And OldArea < 9000000) Or (OldArea > 400000 And OldArea < 600000)) Then 'not close

GoTo NextRec

End If

Case 71

If OldArea < 615 Then

GoTo NextRec

End If

Case 77

If ((OldArea >= 400 And OldArea < 726) Or (OldArea > 400000 And OldArea < 600000) Or (OldArea > 3101000 And OldArea <= 5807000)) Then 'TOO FAR

GoTo NextRec

End If

Case 81

If ((OldArea > 3400000 And OldArea < 3500000) Or (OldArea > 4800000 And OldArea < 4900000)) Then 'too far

GoTo NextRec

End If

Case 87

If ((OldArea >= 3000000 And OldArea <= 7108000) Or OldArea = 8801000) And CurrentFAO = 87 Then

GoTo NextRec

End If

End Select

'conditional use of the area record

If OldArea < 100 Then

NewArea = CurrentFAO

ElseIf (OldArea = 4701100 Or OldArea = 4701000) And CurrentFAO = 34 Then ' 47.1.1 or 47.1 -> 34.3.6

NewArea = 3403600

ElseIf OldArea = 331060 And CurrentFAO = 21 Then 'FAO 31 Bermuda EEZ -> FAO 21 Bermuda EEZ

NewArea = 321060

ElseIf (OldArea = 4801000 Or OldArea = 4802000) And CurrentFAO = 41 Then '48.1 or 48.2 -> 41.3.2

NewArea = 4103200

ElseIf OldArea = 4803000 And CurrentFAO = 41 Then '48.3 -> 41.3

NewArea = 4103000

ElseIf OldArea = 3403600 And CurrentFAO = 47 And (EEZ = 178 Or EEZ = 266) Then '34.3.6 gabon or congo republic -> 47.5

NewArea = 4705000

ElseIf OldArea = 3403600 And CurrentFAO = 47 And EEZ = 180 Then '34.3.6 dem congo republic -> 47.1.1

NewArea = 4701100

ElseIf (OldArea = 549 Or OldArea = 502 Or OldArea = 401 Or OldArea = 400038) And CurrentFAO = 18 Then 'NAFO 0 or 0A or ICES 1

NewArea = 18 ' just possible as it does touch - but much ice

ElseIf OldArea = 3703200 And CurrentFAO = 51 Then '37.3.2 Med sea ->51 (may not work)

NewArea = 51

ElseIf OldArea = 10580 Then 'N Marian 580 EEZ across both areas

NewArea = OldArea

ElseIf OldArea = 4701200 Or OldArea = 4701300 Then

GoTo NextRec 'this area is away from the boundary and specific - leave unaltered

ElseIf (OldArea = 3403500) And CurrentFAO = 47 And EEZ = 266 Then '34.3.5 gabon too far from 47

GoTo NextRec ' not adjacent

ElseIf OldArea = 5254 Or OldArea = 5256 Then

If CurrentFAO <> 61 And CurrentFAO <> 67 Then 'LME area Chukchi Sea - only 18; LME area East Bering Sea, 61 and 67

GoTo NextRec

Else

NewArea = CurrentFAO

End If

ElseIf OldArea = 5805000 And CurrentFAO = 51 Then '58.5 -> 51.7

NewArea = 5107000

ElseIf (OldArea = 5805000 Or OldArea = 5805100 Or OldArea = 5805200) Or CurrentFAO = 57 Then

NewArea = 5704000

ElseIf OldArea = 5257 And CurrentFAO <> 18 Then 'LME area Laptev sea

GoTo NextRec

ElseIf OldArea = 5258 And CurrentFAO <> 18 Then 'LME area Kara sea

GoTo NextRec

ElseIf OldArea = 318840 Then

If CurrentFAO = 61 Or CurrentFAO = 67 Then

NewArea = CurrentFAO

Else

GoTo NextRec

End If

ElseIf OldArea = 426 And CurrentFAO = 34 Then

NewArea = CurrentFAO

ElseIf OldArea = 4804000 And CurrentFAO = 47 Then

NewArea = 4704000

ElseIf OldArea = 4705000 And CurrentFAO = 34 And EEZ = 654 Then

NewArea = 3404100

ElseIf (OldArea = 8802000 Or OldArea = 8803000) And CurrentFAO = 87 Then

NewArea = 8703300

Else

Stop

End If

'do not want NewArea = 0

If NewArea = 0 Then NewArea = FAO(FAOI)

'PropofUnfilled = FAOArea(FAOI) / BadArea 'was based only on areas of FAOs

PropofUnfilled = FAOProp / BadFAOProp 'now based on distn proportion Sept 2010

If PropofUnfilled <= 0 Or PropofUnfilled > 1 Then

Stop

End If

TotalofBad = TotalofBad + PropofUnfilled

CatchtoAdd = TotalLost * PropofUnfilled '*******<<<<<<<<<<<<<<<<<<<<< MAKES SURE NO MORE IS ADDED THAN WHAT WAS REMOVED IN THE STEP BEFORE

If CatchtoAdd < 0.00001 Or CatchtoAdd > 2000000 Then

Stop

End If

'CatchtoAdd = Math.Round(CatchtoAdd, 8)

TotalAdded = TotalAdded + CatchtoAdd

If flgTest = False Then

'===============================================================================================

'APPEND NEW RECORD AS ABOVE BUT THIS FAOAREA AND CATCHTOADD AS CATCH

rstsqlAppend.AddNew()

rstsqlAppend.Fields!Catch.Value = CatchtoAdd '################################# NEW CATCH ADDED HERE

'ACCOUNTING FOR ADDITIONS ---------------------------------------------------------------------

sw.WriteLine("Added," + CStr(rstsqlTax2.Fields!ID.Value) + "," + CStr(rstsqlTax2.Fields!CatchID.Value) + "," + CStr(rstsqlTax2.Fields!CATCHID.Value) + "," + CStr(IYear) + "," + CStr(rstsqlTax2.Fields!CNumber.Value) + "," + CStr(rstsqlTax2.Fields!Taxonkey.Value) + "," + CStr(rstsqlTax2.Fields!Agg.Value) + "," + CStr(CurrentFAO) + "," + CStr(NewArea) + "," + CStr(Math.Round(OrigCatch, 8)) + "," + CStr(Math.Round(SharedCatch, 8)) + ",0," + CStr(CatchtoAdd))

rstsqlAppend.Fields!AreaCode.Value = NewArea

rstsqlAppend.Fields!CatchID.Value = rstsqlTax2.Fields!CatchID.Value

rstsqlAppend.Fields!CATCHID.Value = rstsqlTax2.Fields!CATCHID.Value

rstsqlAppend.Fields!Source.Value = rstsqlTax2.Fields!Source.Value

rstsqlAppend.Fields!Year.Value = rstsqlTax2.Fields!Year.Value

rstsqlAppend.Fields!CATCHgrp.Value = rstsqlTax2.Fields!CATCHgrp.Value

rstsqlAppend.Fields!TaxLevel.Value = rstsqlTax2.Fields!TaxLevel.Value

rstsqlAppend.Fields!TaxonKey.Value = rstsqlTax2.Fields!TaxonKey.Value

rstsqlAppend.Fields!CommonName.Value = rstsqlTax2.Fields!CommonName.Value

rstsqlAppend.Fields!TaxonName.Value = rstsqlTax2.Fields!TaxonName.Value

rstsqlAppend.Fields!SuperTargetGrp.Value = rstsqlTax2.Fields!SuperTargetGrp.Value

rstsqlAppend.Fields!ISSCAAP.Value = rstsqlTax2.Fields!ISSCAAP.Value

rstsqlAppend.Fields!TargetGrpNum.Value = rstsqlTax2.Fields!TargetGrpNum.Value

rstsqlAppend.Fields!ClaCode.Value = rstsqlTax2.Fields!ClaCode.Value

rstsqlAppend.Fields!FAOArea.Value = CurrentFAO

rstsqlAppend.Fields!OrdCode.Value = rstsqlTax2.Fields!OrdCode.Value

rstsqlAppend.Fields!CNumber.Value = rstsqlTax2.Fields!CNumber.Value

rstsqlAppend.Fields!HighSeas.Value = rstsqlTax2.Fields!HighSeas.Value

rstsqlAppend.Fields!DWFEEZ.Value = rstsqlTax2.Fields!DWFEEZ.Value

rstsqlAppend.Fields!OwnEEZ.Value = rstsqlTax2.Fields!OwnEEZ.Value

rstsqlAppend.Fields("FAO Name").Value = rstsqlTax2.Fields("FAO Name").Value

rstsqlAppend.Fields!Agg.Value = rstsqlTax2.Fields!Agg.Value

rstsqlAppend.Fields!OrigTaxon.Value = rstsqlTax2.Fields!OrigTaxon.Value

rstsqlAppend.Fields!Distant.Value = rstsqlTax2.Fields!Distant.Value

rstsqlAppend.Fields!Prob.Value = rstsqlTax2.Fields!Prob.Value

rstsqlAppend.Fields!Added.Value = CatchtoAdd

rstsqlAppend.Update()

'sw.WriteLine("Add" + "," + CStr(IYear) + ",0," + CStr(rstsqlAppend.Fields!CatchID.Value) + "," + CStr(rstsqlAppend.Fields!CATCHID.Value) + "," + CStr(rstsqlAppend.Fields!FAOArea.Value) + "," + CStr(rstsqlAppend.Fields!AreaCode.Value) + "," + CStr(Math.Round(CatchtoAdd, 5)))

'Else

'reduce catch in existing record

' CatchtoKeep = LeftCatch * FAOArea(FAOI) / GoodArea

' TotalLeft = TotalLeft + CatchtoKeep

'====================================================================================================================

Else

'Stop

End If

End If

Next FAOI

NextRec:

rstsqlTax2.MoveNext()

Next Rec 'each record of each taxa/year/EEZ Country -NEXT CATCH RECORD

'check totals

If Math.Abs(TotalAdded - TotalLost) > 0.001 Then

Stop

End If

If Math.Abs(TotalofBad - 1) > 0.001 Then

'Stop marked out because it keeping stopping here??? NOv 2016

End If

rstsqlTax2 = Nothing

rstsqlAppend = Nothing

NextTax:

Next Tax 'each taxa within the year and EEZ Country

End If 'if any problem FAO areas within EEZ country

End If 'if there is any catch reported by EEZ country

Next IYear 'END OF FOR EACH YEAR

Next Agg

rstsql.MoveNext()

Next CNum 'NEXT MULTI-FAO EEZ COUNTRY

'CLOSE ALL OBJECTS

sw.Flush()

sw.Close()

Windows.Forms.Cursor.Current = Windows.Forms.Cursors.Default

frm.Noticefrm(1, "Holes Filled")

End Function

**5. Mapping**

Public Function allocationNET2(ByVal formAllo As frmAllo, ByVal pickSQL As String, ByRef CATCHparas As CATCHvariables.Globals) As Boolean

Dim currentRec As New CATCHvariables.CatchRecord ' udtCATCH3data

Dim cntry As countryCodes

Dim eezTable As New DataTable

Dim k As Long = 0

Dim CNumber As Long = 0

Dim RootName As String 'path for output

'Dim AppPath As String

Dim SpeciesPath As String

Dim AreaPath As String

'Dim RichNotice As String

Dim recCatch As New DataTable

Dim NewCountry, NumRecords, i As Integer

Dim OutFile As String 'the file and path for the current output file

Dim percentWeightError As String = "0.000%"

Dim computerName As String

Dim logID As String = Trim(Now.Year.ToString) & String.Format(Now.Month, "00") & String.Format(Now.Day, "00")

Dim SPECx() As Long

Dim StatAreax() As Long

'Dim tg_Access As CATCHcells.Cell_TG_Access_Container

Dim flgHarvestDistExists As Boolean = False

Dim flgDefaultSpUsed As Boolean = False

Dim OldCountry As Integer = 0

Dim OldYear As Integer = 0

Dim OldArea As Integer = 0

Dim LastTaxaFile As String = ""

Dim reAllo As Boolean = False

Dim Taxonkey As Long

Dim IYear As Long

Dim UseYear As Long

Dim flgFirst As Boolean = True

Dim line As String

Dim SFileName As String

Dim SpIndex As Long

Dim flgNoAnnual As Boolean = False

Dim flgDistant As Boolean

Dim OldflgDistant As Boolean

Dim Source As Long

Dim Tonnes As Double

Dim CATCHID As Long

Dim Problem As Boolean

Dim KeepUseYear As Long

Dim CatchID As Long = 0

Dim Strike As Long = 0

Dim MaxStrike As Long = 3

Dim flgMother As Boolean = False

Dim KeepOldCountry As Integer = 0

Dim KeepNewCountry As Integer = 0

Dim ret As Boolean = False

Dim BumpedTaxon As Long = 0

Dim Direction As String = ""

Dim LYear As Long = 0

Dim FoundYear As Long = 0

Dim flgIndexProblem As Boolean = False

Dim flgTaxonChanged As Boolean = False

Dim flgCountryChanged As Boolean = False

Dim keepCountryAlpha As String = ""

Dim keepCNumber As Long = 0

Dim keepCountryName As String = ""

Dim keepTaxonName As String = ""

Dim keepCommonName As String = ""

Dim keepTaxa As Long = 0

Dim Method As Long = 0

Dim SuccessFile As String = ""

Dim MethodFile As String = ""

Dim flgWriteSuccess As Boolean = False

Dim MissedCatch As Double = 0

Dim TotalProcessed As Double = 0

Dim strbits() As String

Dim StickyFactor As Single

'ini variables

Dim iniSection As String = "CATCHDATA"

Dim flgfileOverride As Boolean = False 'true 'if it all goes in one output file or not

Dim swReadFidelity As IO.StreamReader

Dim swFidelity As IO.StreamWriter

Dim FidelityFile As String = "FidelityUsed.csv"

Dim FidelityFile2 As String = "FidelityUsed2.csv"

CJobStart:

CJobText = CStr(CJobList(CurrentCJob))

Dim iniInfo As New iniFile(System.AppDomain.CurrentDomain.BaseDirectory() & "\ini\CATCH.ini")

logID = Trim(Now.Year.ToString) & String.Format(Now.Month, "00") & String.Format(Now.Day, "00")

If flgUseCGroups Then

FidelityFile = BaseDirectory + "FidelityUsed" + CJobText + ".csv"

FidelityFile2 = BaseDirectory + "FidelityUsed2" + CJobText + ".csv"

End If

'Jan 25 2011

If flgEnableFidelity Then

'March 2011.. give up on post processing (TOO SLOW) and try active approach

ReDim ExitYear(999)

'all countries set to default value

For i = 1 To 999

ExitYear(i) = -1

Next

'all cells set to no claim by countries

'ReDim Claims(MaxPos, 999)

'For k = 1 To MaxPos

'For i = 1 To 999

'Claims(k, i) = False

'Next

'Next

'set claims by countries

'For i = 0 To eezTable.Rows.Count - 1

'k = eezTable.Rows(i).Item("Seq")

'CNumber = eezTable.Rows(i).Item("CNumber")

'If CNumber > 0 Then

' Claims(k, CNumber) = True

'End If

'Next i

'is this starting from 1950 and a new FidelityUsed created or do we read the present and proceed??

'if FirstYear > 1950 then read the FidelityUsed

If flgStart1950 = False Then

Try

If IO.File.Exists(FidelityFile) Then

swReadFidelity = New IO.StreamReader(FidelityFile)

line = swReadFidelity.ReadLine() 'labels

Do

line = swReadFidelity.ReadLine()

If line Is Nothing Then

Exit Do

Else

'parse line

strbits = line.Split(",")

CNumber = strbits(0) 'CNumber

IYear = strbits(1) 'Year

StickyFactor = strbits(2) 'Mid Distance

'use contents to update the ExitYear(CNumber) array

ExitYear(CNumber) = strbits(3) 'Max Distance

End If

Loop

swReadFidelity.Close()

swReadFidelity = Nothing

Else

Stop 'Fidelity file does not exist

End If

'rename the FidelityUsed

If IO.File.Exists(FidelityFile2) = False Then

My.Computer.FileSystem.RenameFile(FidelityFile, FidelityFile2)

Else

Stop 'New Fidelity File already exists

End If

Catch

'Stop 'error reading Fidelity file

End Try

End If

OldFive = 0

'schedule of exit allowed by year in proportion '======================================================================

Allowance(0) = 0.05

Allowance(1) = 0.1

Allowance(2) = 0.2

Allowance(3) = 0.4

Allowance(4) = 0.6

'===============================================================================================================================

swFidelity = New IO.StreamWriter(FidelityFile)

swFidelity.WriteLine("CNumber,Year,Sticky,ExitYear")

swFidelity.Flush()

End If

Try

Windows.Forms.Cursor.Current = Windows.Forms.Cursors.WaitCursor

'set up paths and defaults

areaGrid.set_maxCellNumber(MaxPos)

frm = formAllo

If frm.chkYrsRev.Checked = True Then Direction = "DEC" Else Direction = "INC"

If Direction = "DEC" And flgEnableFidelity Then Stop

'ARMING METHODS BASED ON FORM CHOICES

With frm

If .chkAllowBump.Checked Then

FLGALLOWBUMPUPTAXA = True

Else

FLGALLOWBUMPUPTAXA = False

End If

If .chkTaxaChng.Checked Then

FLGALLOWREVERSEDISAG = True

Else

FLGALLOWREVERSEDISAG = False

End If

If .chkAllowAltDist.Checked Then

FLGALLOWALTTAXA = True

Else

FLGALLOWALTTAXA = False

End If

If .chkAllowStatic.Checked Then

FLGALLOWSTATICOVERRIDE = True

Else

FLGALLOWSTATICOVERRIDE = False

End If

If .chkMarineAnimals.Checked Then

FLGALLOWANIMAL = True

Else

FLGALLOWANIMAL = False

End If

If .ChkProblemsOnly.Checked Then

flgWriteSuccess = True

Else

flgWriteSuccess = False

End If

End With

FLGALLOWDEFLAG = True 'ALWAYS ALLOW DEFLAGGING

ComProc = New Common

computerName = ComProc.GetComputerName.ToUpper

'If frm.paraCATCH3local.Checked = True Or frm.ParaMDB.Checked = True Then

'CATCHparas = New CATCHvariables.Globals

'CATCHparas.setGlobalDefaults2()

AreaPath = CATCHparas.pGridFiles_AreaAccessC & "\"

SpeciesPath = CATCHparas.pGridFiles_SpDistribution & "\"

CATCHparas.pathA = CATCHparas.pListFiles & "\CellData.csv" 'not used anymore, data straight from world table

CATCHparas.pathB = CATCHparas.pListFiles & "\" & "Problems_" & logID & "_" & CJobText & "_" & computerName & ".csv"

CATCHparas.pathC = frm.txtProgressPath.Text & "\Progress_" & logID & "_" & CJobText & "_" & computerName & ".csv"

CATCHparas.pathD = CATCHparas.pListFiles & "\" & "TaxaChangeLog_" & logID & "_" & CJobText & "_" & computerName & ".csv"

RootName = BaseDirectory + "RESULTS\CatchImport"

'RootName = iniInfo.GetString(iniSection, "pResults", "E:\RWatson\RESULTS\CatchImport")

If flgAllowStatic Then

ProbAnnualFile = CATCHparas.pListFiles & "\" & "Problems_" & logID & "_" & CJobText & "_" & computerName & "_Annuals.csv"

writeOutputData(ProbAnnualFile, "Year,Source,CATCHID,AID,TargetGrpNum,TaxonName, CommonName, TaxonKey,CountryName,CountryCode,Area, SpCount,CoCount,ACount,Catch,FirstFlag,FirstName,Comment,Agg,OrigTaxon,AreaSp,AreaAccess,AccSpec,UseYear", False)

End If

SuccessFile = CATCHparas.pListFiles & "\" & "Success_" & logID & "_" & computerName & ".csv"

MethodFile = BaseDirectory + "RESULTS\Method_" & logID & "_" & CJobText & ".csv"

prog = New progress(formAllo, CATCHparas)

'#IO# read in world cell data world table

frm.Noticefrm(1, "Loading Data ...")

frm.Noticefrm(2, "")

If Read_CellData(CATCHparas.pMDB_OceanSq) = False Then Throw New SystemException("Error getting Cell Data")

'Jan 25 2011

'Remoteness

If flgEnableRemoteness Then

'dimention Accessiblity array

'read data into array

'Public Accessibility() As Double 'a factor from 1 to 0 that is 1-remoteness where remoteness is normalised avg of distance to nearest port + distance to any land

If Read_Accessibility(CATCHparas) = False Then Throw New SystemException("Error getting Accessibility Data")

End If

'Read the year of distribution that should be used for each year for these annual species (all years not there to save room)

If Not flgAnnualRead Then

ret = AnnualRead(SpeciesPath)

If ret = False Then Throw New SystemException("Could not read the Species list")

End If

'#IO# load taxa info

taxaTable = ComProc.getDataTable(CATCHparas.pMDB_Taxon, "SELECT TaxonNom.TaxonKey, TaxonNom.ISSCAAP, TaxonNom.ClaCode, TaxonNom.OrdCode, TaxonNom.FamCode, TaxonNOm.GenCode, TaxonNom.SpeCode, TaxonNom.TaxLevel " _

& "FROM TaxonNom " _

& "ORDER BY TaxonNom.TaxonKey, TaxonNom.ISSCAAP, TaxonNom.ClaCode, TaxonNom.OrdCode, TaxonNom.FamCode, TaxonNom.GenCode, TaxonNom.SpeCode")

If taxaTable Is Nothing Then Throw New SystemException("Error getting taxa data")

'#IO# load re-flag data

reflagTable = ComProc.getDataTable(CATCHparas.pMDB_CATCH_main, "SELECT CNumber, FAOName, Rank, ReflagCNumber, ReflagName FROM REFLAG")

'#IO# loading data for Access Object

'eezTable = ComProc.getDataTable(CATCHparas.pMDB_OceanSq, "SELECT worldEEZ.Seq, worldEEZ.CNumber, worldEEZ.Pwater, worldEEZ.Area, worldEEZ.Disputed FROM worldEEZ") 'disputed

eezTable = ComProc.getDataTable(CATCHparas.pMDB_OceanSq, "SELECT worldEEZ.Seq, worldEEZ.CNumber, worldEEZ.Pwater, worldEEZ.Prop, worldEEZ.Disputed FROM worldEEZ") 'disputed rw 7 June 2009

cntry = New countryCodes(CATCHparas.pMDB_CATCH_main, System.AppDomain.CurrentDomain.BaseDirectory() & "ini\CATCH.ini")

'cntry = New countryCodes(CATCHparas.pMDB_CATCH_main, CATCHparas)

'Private accessDT As DataTable

cAccess = New cellAccess(cntry, CATCHparas, eezTable)

eezTable.Clear()

eezTable.Dispose()

'#IO# write new Problems File

writeOutputData(CATCHparas.pathB, "Year,Source,CATCHID,AID,TargetGrpNum,TaxonName, CommonName, TaxonKey,CountryName,CountryCode,Area, SpCount,CoCount,ACount,Catch,FirstFlag,FirstName,Comment,Agg,OrigTaxon,AreaSp,AreaAccess,AccSpec,UseYear", False)

'#IO# write new Success File

If flgWriteSuccess Then writeOutputData(SuccessFile, "Year,Source,CATCHID,AID,TargetGrpNum,TaxonName, CommonName, TaxonKey,CountryName,CountryCode,Area, SpCount,CoCount,ACount,Catch,FirstFlag,FirstName,Comment,Agg,OrigTaxon,AreaSp,AreaAccess,AccSpec,UseYear", False)

'^^^^^^^^^^^^^^^^^^ PROGRESS REPORT ^^^^^^^^^^^^^^^^^^^^^^^^^^^^^^^^^^^^^^^^^^^^^^^^^^^^^^^

'#IO# write new Progess file File

writeOutputData(CATCHparas.pathC, "Year,Time,Date,HrsYr,HrsElapsed,SecPer,NumLeft,HrsLeft,NumOKYr,NumErrYr,PerErrNYr,PerErrTYr,TonProcessed,TonAlloYr,TonNotAlloYr,RowsWritten", False)

'If frm.chkTaxaChng.Checked = True Then

''#IO# write new AreaChangeLog file File

'writeOutputData(CATCHparas.pathD, "CATCH3ID, Area, CNumber, taxaCode, Year, Catch, OrigTaxa", False)

'End If

writeOutputData(MethodFile, "AID, Method, NewCode, OldCode", False)

'#IO# read in CATCH3 data

recCatch = ComProc.getDataTable(CATCHparas.pMDB_CATCH_main, pickSQL)

If recCatch Is Nothing Then Exit Function

NumRecords = recCatch.Rows.Count

'fill progress variables

prog.RichNotice = "Ver: " & Now.ToString("y", System.Globalization.DateTimeFormatInfo.InvariantInfo)

prog.setStartTime()

prog.recTotal = NumRecords

frm.ProgressBar1.Maximum = NumRecords

frm.ProgressBar1.Visible = True

'loop through CATCH3 records

frm.Noticefrm(1, "Allocating Data..........")

For i = 0 To recCatch.Rows.Count - 1

frm.Noticefrm(2, CStr(i + 1) + " : " & CStr(NumRecords) & " - Weight Error " & String.Format("{0:n3}", percentWeightError) & " (" & CStr(Counterr) & ")")

'flgReflag = False 'reset country reflagging flag - IT IS NOW ARMED

'flaginfo = Nothing

'APRIL 8 2009

'FLAGS TO CONTROL THE METHODS TO MAKE TAXONOMIC PROBLEMS WORK

'FLAGS ARE SET ACCORDING TO CHOICES ON FORM AND IF SET

'ALLOW A METHOD TO BE USED ONCE

'ONLY AFTER ALL METHODS EXHAUSTED SHOULD THE PROBLEM BE ACCEPTED AND WRITTEN TO FILE

'FAILURE TO ALLOCATE IS DETECTED IN THE CALCOUTPUT FUNCTION AND A FLAG REALLO RETURNED TRUE TO THIS ROUTINE FOR FUTHER ATTEMPTS

'UNLESS ALL METHODS ARE EXHAUSTED

'NEED THREE VARIABLES FOR EACH METHOD

'ONE TO ALLOW IT FLGALLOW...

'ONE TO ARM IT FOR A RECORD FLGARM....

'ONE TO SAY IT IS IN PROCESS FLGDOING....

'WHEN A ATTEMPT AT ALLOCATION IS MADE (CALL TO CALCOUTPUT)

'IT SHOULD RETURN FLGFAILED = TRUE IF IT FAILS

'THEN DECISION SHOULD ALL BE MADE IN THIS ROUTINE

'METHODS ARRANGED IN ORDER OF PREFERENCE

'IF ARMED FLGDOING IS FALSE THEN THE FLGDOING IS SET AND IT IS SET BACK UP WITH CHANGES MADE

'IF ARMED AND FLGDOING IS TRUE THEN REVERSE CHANGES AND PASS ON TO THE NEXT METHOD BELOW

'BELOW METHODS IT THE CALL TO WRITE PROBLEMS... MAKE SURE THIS IS NOT CALL FROM ELSEWHERE.

'~~~~~~~ INPUT DATA ~~~~~~~~~~~~~~~~~~~~~~~~~~~~~~~

LoadDataRow:

errorSpot = "Load row data"

Source = 0

NewCountry = 0

Tonnes = 0

OrigArea = 0

OrigTaxa = 0

CurrentTaxon = 0

AID = 0

currentRec = loadRowData(recCatch.Rows(i), Strike, MaxStrike)

'doing this now may stall enough to make the data always available

'ARMING OF ALTERNATIVE METHOD FLAGS (for each record) ===================================================

If FLGALLOWALTTAXA Then FLGARMALTTAXA = True Else FLGARMALTTAXA = False

If FLGALLOWBUMPUPTAXA Then FLGARMBUMPUPTAXA = True Else FLGARMBUMPUPTAXA = False

If FLGALLOWREVERSEDISAG Then FLGARMREVERSEDISAG = True Else FLGARMREVERSEDISAG = False

If FLGALLOWSTATICOVERRIDE Then FLGARMSTATICOVERRIDE = True Else FLGARMSTATICOVERRIDE = False

If FLGALLOWDEFLAG Then FLGARMDEFLAG = True Else FLGARMDEFLAG = False

If FLGALLOWANIMAL Then FLGARMANIMAL = True Else FLGARMANIMAL = False

FLGDOINGALTTAXA = False

FLGDOINGBUMPUPTAXA = False

FLGDOINGREVERSEDISAG = False

FLGDOINGSTATICOVERRIDE = False

FLGDOINGDEFLAG = False

FLGDOINGANIMAL = False

flgTaxonChanged = False

flgCountryChanged = False

'========================================================================================================

'check if data complete and read properly

Source = currentRec.Source

NewCountry = currentRec.CountryCode '.FishCountry

'If NewCountry = 74 Then Stop

OldFlag = currentRec.CountryCode

OldCountryName = currentRec.CountryName

OrigArea = currentRec.AreaCode '.NewArea

OrigTaxa = currentRec.OriginalTaxon

CurrentTaxon = currentRec.TaxonKey

flgDistant = currentRec.Distant

Tonnes = currentRec.Catch

'catch not acceptable

If Tonnes <= 0.0000001 Or Tonnes > 99999999 Then

Tonnes = 0

GoTo nextrec

End If

'CatchID = currentRec.CATCHID

'CATCHID = currentRec.CATCHID

AID = currentRec.AID

FoundYear = 9999

Method = 0

'If NewCountry = 999 Then Stop

'If Tonnes = 0 Then Stop

'If AID = 16680 Then Stop

'If CATCHID = 122181 Then Stop

If AID = 0 Or Source = 0 Or NewCountry = 0 Or OrigArea = 0 Or CurrentTaxon = 0 Or Tonnes = 0 Then

Strike = Strike + 1

If Strike < MaxStrike Then

GoTo LoadDataRow 'try again

Else

'presume it is OK otherwise and pass on

Strike = 0

MissedCatch = MissedCatch + Tonnes

'might write there out as they should be read errors if all these fields have been checked in CATCH3 before allocation run

End If

End If

'~~~~~~~~~~~~~~~~~~~~~~~~~~~~~~~~~~~

'~~~ NewSTabName = "S" + CStr(currentRec.TaxonKey) 'Name for the species file

'

'============================ NEW YEAR ========================================================

'Need a new file for another decade?

If OldYear <> currentRec.Year Then

errorSpot = "New Year"

If flgUseCGroups Then

If OldYear = 0 Then

'name by the cgroup and assume all years done

swAlloc = New IO.StreamWriter(RootName + "_C" + CJobText + ".csv")

swAlloc.AutoFlush = False

swAlloc.WriteLine("ID,IYear,CNumber,TaxonKey,Cell,EEZ,CatchRate")

Else

'keep same file

End If

Else

'Set up first Output file based on Year - required because the files get too large otherwise

If frm.chkProblems.Checked = False Then

If flgfileOverride <> True Then

If i = 0 Then OutFile = writeOutputFile(RootName, currentRec.Year, OutFile, True) Else OutFile = writeOutputFile(RootName, currentRec.Year, OutFile)

Else

'just one file for all the results

If i = 0 Then OutFile = writeOutputFile(RootName, currentRec.Year, OutFile, True)

End If

If OutFile = "NoFile" Then Throw New SystemException("Error writing output file")

End If

End If

'^^^^^^^^^^^^^^^^^^ PROGRESS REPORT ^^^^^^^^^^^^^^^^^^^^^^^^^^^^^^^^^^^^^^^^^^^^^^^^^^^^^^^

If i <> 0 Then prog.progressWrite(CATCHparas.pathC, OldYear, Counterr, CountOK, TotalProcessed, TotalTonnes, TotalTonnesNotAllo, i)

'"Year,Time,Date,MinYr,MinElapsed,SecPer,NumLeft,HrsLeft,NumOKYr,NumErrYr,PerErrNYr,PerErrTYr,Tonnesprocessed,TonAlloYr,TonNotAlloYr,RowWritten"

OldYear = currentRec.Year

TotalProcessed = 0

End If

'=================================================================================================

''########## TESTER ##################################

'If i < 34761 Then GoTo nextrec

'If AID <> 783490 Then

'GoTo nextrec

'Else

'Stop

'End If

'If NewCountry <> 70 And NewCountry <> 999 Then GoTo nextrec

'If currentRec.TaxonKey <> 690009 Then GoTo nextrec

'If NewCountry <> 620 Or OrigArea <> 21 Or currentRec.TaxonKey <> 600069 Then GoTo nextrec

'If NewCountry <> 233 And NewCountry <> 428 And NewCountry <> 440 And NewCountry <> 643 And NewCountry <> 804 Then GoTo nextrec

'If OrigArea = 10016 Then Stop

'If AID = 443008 Then Stop

'all catch accounted for here... must be lost afterward

TotalProcessed = TotalProcessed + Tonnes

StartTaxa:

'============================ NEW SPECIES ========================================================

If OldTaxa <> CurrentTaxon Or OldflgDistant <> flgDistant Or SPECx Is Nothing Then

LastTaxaFile = "" 'do not allow reversion to a previous year for annual harvest files (interpolation and extrapolation) - rw Jan/07

errorSpot = "Annual Species"

'BUMP UP ================================================================

If FLGDOINGBUMPUPTAXA = True Then

OldTaxa = 0

BumpedTaxon = 0

BumpedTaxon = getNextLevelTaxaCode(CurrentTaxon)

Do Until BumpedTaxon <> 0

'wait for return - required as there can a delay in value coming back

System.Windows.Forms.Application.DoEvents()

Loop

CurrentTaxon = BumpedTaxon

If CurrentTaxon < 0 Then

FLGARMBUMPUPTAXA = False

flgAllocationfailed = True

GoTo Alternatives

End If

End If

'=============================================================================

flgHarvestDistExists = checkAnnualDist(SpeciesPath & strAltPath, CurrentTaxon) 'modified by rw Jan/07 to detect annual demersal harvest as well as tuna/billfish

If flgHarvestDistExists Then

'test if we should ignore

If flgDistant = True And flgTuna = False Then

flgNoAnnual = True

Else

flgNoAnnual = False

flgIndexProblem = False

'ensure a new file if the annual distribution/harvest file to be used changes even if same Taxonkey

Try

If AnnualList(SpList(CurrentTaxon), OldYear - CATCHparas.minYear) <> AnnualList(SpList(CurrentTaxon), currentRec.Year - CATCHparas.minYear) Then

'If flgHarvestDistExists = True And OldYear <> currentRec.Year Then

OldTaxa = 9999

End If

Catch

'get date manually

UseYear = 9999

flgIndexProblem = True

For LYear = currentRec.Year To 1950 Step -1

SFileName = SpeciesPath & strAltPath & "S" & CStr(CurrentTaxon) & "_" + CStr(LYear) + ".csv"

If IO.File.Exists(SFileName) Then

UseYear = LYear

OldTaxa = 9999

FoundYear = UseYear

GoTo FoundYearNow

End If

Next LYear

FoundyearNow:

If UseYear = 9999 Then

'no files exist

flgAllocationfailed = True

GoTo Alternatives

End If

Catch ex As Exception

End Try

End If

End If

' get species cell proportions

If OldTaxa <> CurrentTaxon Or SPECx Is Nothing Then 'New Species to load

'SPECIES TABLE EXISTS (OR USE DEFAULT) ****************************************************

flagTaxa:

errorSpot = "Get taxa Array"

'strAltPath = "" ' normally do not use the alt distribution folder

If flgNoAnnual = True Or flgOverrideAnnual = True Then flgUseStatic = True

'get maxpos array of species cell proportion

flgTaxaFile:

If FLGDOINGALTTAXA Then

strAltPath = "ALT\"

Else

strAltPath = ""

End If

If flgHarvestDistExists And Not FLGDOINGSTATICOVERRIDE And Not flgUseStatic Then

'lookup and use the relevant year of distribution

If Not flgIndexProblem Then

If FoundYear > 9999 Then

UseYear = AnnualList(SpList(CLng(CurrentTaxon)), currentRec.Year - CATCHparas.minYear)

Else

'find a year manually

UseYear = 9999

flgIndexProblem = True

For LYear = currentRec.Year To 1950 Step -1

SFileName = SpeciesPath & strAltPath & "S" & CStr(CurrentTaxon) & "_" + CStr(LYear) + ".csv"

If IO.File.Exists(SFileName) Then

UseYear = LYear

OldTaxa = 9999

FoundYear = UseYear

GoTo FoundYearNow2

End If

Next LYear

FoundyearNow2:

If UseYear = 9999 Then

'no files exist

flgAllocationfailed = True

GoTo Alternatives

End If

End If

Else

UseYear = FoundYear

End If

SFileName = SpeciesPath & strAltPath & "S" & CStr(CurrentTaxon) + "_" & CStr(UseYear) & ".csv"

'FileName = spPath & "S" & LastTaxaFile + "_" & Trim$(Str$(newYr)) & ".csv"

ElseIf Not flgHarvestDistExists Or FLGDOINGSTATICOVERRIDE Or flgUseStatic Then

SFileName = SpeciesPath & strAltPath & "S" & CStr(CurrentTaxon) & ".csv"

KeepUseYear = UseYear

UseYear = 9999

End If

SPECx = getTaxaArray(SFileName, SpeciesPath, Trim(Str(CurrentTaxon)), LastTaxaFile, currentRec.Year, flgHarvestDistExists, flgDefaultSpUsed, SCount)

OldflgDistant = flgDistant

If SPECx Is Nothing Then

flgAllocationfailed = True

GoTo Alternatives

End If

OldTaxa = CurrentTaxon

End If

End If 'end of reading in species information

'=================================================================================================

'@@@@@@@

'RW Feb18 ANTS indicated change - get the area first and filter access by this

'USE THIS SET OF CELLS TO FILTER WORK ON ANY OF THE OTHER LAYERS (FISHING ACCESS AND TAXON)

'flagArea:

'============================ AREA ACCESS ========================================================

'Use new Area System

'Multiple look up

'Get the superset of IDH and set up statareax(k) for positions

'If currentRec.AreaCode = 21 Then Stop

If currentRec.AreaCode <> OldArea Or StatAreax Is Nothing Then

errorSpot = "Get Area Array"

' JS: release StatArea

StatAreax = Nothing

' JS: clean up, this hopefully will discharge the memory

GC.Collect()

StatAreax = getAreaArray(AreaPath, currentRec, ACount)

If StatAreax Is Nothing Then

Throw New System.Exception("Area Gridfile " + CStr(currentRec.AreaCode) + " not available")

GoTo nextrec

'########### write to problem file

End If

OldArea = currentRec.AreaCode

End If 'NEW AREA

'@@@@@@@

flagCountry:

'============================ COUNTRY ACCESS =====================================================

If NewCountry = 0 Then NewCountry = 999

If OldCountry <> NewCountry Or OldYear <> currentRec.Year Then

'########### LOAD NEW ACCESS OBJECT ######################################

'#########################################################################

If flgEnableFidelity Then

'deal with change in oldCountry status

If flgCountryExit = False Then

'Stop

If OldCountry <> 9999 Then ExitYear(OldCountry) = -1

End If

'presumed that it did not leave its waters

flgCountryExit = False 'has to leave home waters to change this

'Newcountry

If ExitYear(NewCountry) = -1 Then

StickyRatio = Allowance(0)

Else

TimeGone = currentRec.Year - ExitYear(NewCountry)

'If TimeGone > 0 Then Stop

If TimeGone < 0 Then

StickyRatio = Allowance(0)

'Stop 'is the allocation doing the years in reverse?

ElseIf TimeGone >= 0 And TimeGone < MaxExit Then

StickyRatio = Allowance(TimeGone)

ElseIf TimeGone >= MaxExit Then

StickyRatio = 1

Else

Stop

End If

End If

If flgRecordFidelity Then

swFidelity.WriteLine(CStr(NewCountry) + "," + CStr(currentRec.Year) + "," + CStr(StickyRatio) + "," + CStr(ExitYear(NewCountry)))

swFidelity.Flush()

End If

End If

''Just change the EEZ's allowed for the country reporting

''build a list of EEZ codes allowed put 0 at the first position in the vector

''will accept pos for catch processing where there is match

''build fishing access array

errorSpot = "Get Access Array"

cAccess.buildAccessArray(NewCountry, currentRec.Year)

OldCountry = NewCountry

End If 'NEW COUNTRY

'=================================================================================================

'=================================================================================================

'============================ CALCULATE OUTPUT ===================================================

CalcOutput:

flgExit = False

errorSpot = "Calc Output" 'see if it worked and we can allocate catch

currentRec.TaxonKey = CurrentTaxon

flgAllocationfailed = True

If Not SPECx Is Nothing Then

flgAllocationfailed = calcOutput(currentRec, OutFile, NewCountry, StatAreax, SPECx, UseYear, KeepUseYear, flgDistant, flgCountryChanged, flgTaxonChanged)

End If

If flgAllocationfailed = False And Method <> 0 Then

'write a record of what happened and code change

Select Case Method

Case 1 'reflagging

writeOutputData(MethodFile, CStr(AID) & "," & CStr(Method) & "," & CStr(NewCountry) & "," & CStr(OldFlag), True)

Case 2 'alternative distn (mostly tuna)

writeOutputData(MethodFile, CStr(AID) & "," & CStr(Method) & "," & CStr(currentRec.TaxonKey) & "," & CStr(currentRec.TaxonKey), True)

Case 3 'static override (for those with annual harvest distn)

writeOutputData(MethodFile, CStr(AID) & "," & CStr(Method) & "," & CStr(currentRec.TaxonKey) & "," & CStr(currentRec.TaxonKey), True)

Case 4 'bumpup taxa

writeOutputData(MethodFile, CStr(AID) & "," & CStr(Method) & "," & CStr(currentRec.TaxonKey) & "," & CStr(keepTaxa), True)

Case 5 'reverse disagg

writeOutputData(MethodFile, CStr(AID) & "," & CStr(Method) & "," & CStr(currentRec.TaxonKey) & "," & CStr(keepTaxa), True)

Case 6 'marine animals

writeOutputData(MethodFile, CStr(AID) & "," & CStr(Method) & "," & CStr(currentRec.TaxonKey) & "," & CStr(keepTaxa), True)

Case Else

Stop

End Select

End If

'======================================================================================================================

Alternatives:

If flgAllocationfailed = True Then

FoundYear = 9999 'if you need a manually located year do it again

flgIndexProblem = False 'unflag problems getting a harvest year to use

'method that tries to deflag vessels to their original country flag (and fishing accesss)

If FLGDOINGDEFLAG Then

If curFlagNum >= flaginfo.GetUpperBound(0) Then

'stop attempting to deflag

FLGARMDEFLAG = False

FLGDOINGDEFLAG = False

Else

curFlagNum = curFlagNum + 1

'Try with New Code

currentRec.CountryCodeAlpha = flaginfo(curFlagNum).DeflagCodeA

NewCountry = flaginfo(curFlagNum).DeflagCode

currentRec.CountryName = flaginfo(curFlagNum).DeflagName

OldCountry = 9999

Method = 1

GoTo starttaxa

End If

ElseIf FLGARMDEFLAG And Not FLGDOINGDEFLAG And Not SPECx Is Nothing Then

FLGDOINGDEFLAG = True

OldFlag = NewCountry

flaginfo = Reflag(NewCountry)

If flaginfo Is Nothing = False Then

FLGDOINGDEFLAG = True

curFlagNum = 0

'Try with first New Code

keepCountryAlpha = currentRec.CountryCodeAlpha

keepCountryName = currentRec.CountryName

keepCNumber = NewCountry

currentRec.CountryCodeAlpha = flaginfo(curFlagNum).DeflagCodeA

NewCountry = flaginfo(curFlagNum).DeflagCode

currentRec.CountryName = flaginfo(curFlagNum).DeflagName

OldCountry = 9999

flgCountryChanged = True

Method = 1

GoTo starttaxa

Else

'stop attempting to deflag

FLGDOINGDEFLAG = False

flgCountryChanged = True

If flaginfo Is Nothing = False Then

currentRec.CountryCodeAlpha = keepCountryAlpha

currentRec.CountryName = keepCountryName

NewCountry = keepCNumber

End If

End If

End If

Method = 0

FLGARMDEFLAG = False 'method used up

'method to try alternative taxa distribution (significant for tuna especially)

If FLGDOINGALTTAXA Then

FLGDOINGALTTAXA = False

ElseIf (FLGARMALTTAXA Or flgTuna) And Not FLGDOINGDEFLAG Then

FLGDOINGALTTAXA = True

'set it up to go

OldTaxa = 9999

Method = 2

GoTo flgTaxaFile

End If

Method = 0

FLGARMALTTAXA = False 'method used up

'method that allows a static distribution for annual species

If FLGDOINGSTATICOVERRIDE Then

'trying static failed

FLGDOINGSTATICOVERRIDE = False

'reverse to get the year again

UseYear = KeepUseYear

ElseIf FLGARMSTATICOVERRIDE And Not FLGDOINGSTATICOVERRIDE And flgHarvestDistExists And Not FLGDOINGBUMPUPTAXA Then

FLGDOINGSTATICOVERRIDE = True

'set it up to go

OldTaxa = 9999

Method = 3

GoTo flgTaxaFile

End If

Method = 0

FLGARMSTATICOVERRIDE = False 'method used up

'method that reverses taxa from specific to general until it cannot find more general or has used all levels

If FLGDOINGBUMPUPTAXA And FLGARMBUMPUPTAXA = False Then

'bump up failed

'reverse taxa to the original

CurrentTaxon = keepTaxa

currentRec.TaxonKey = keepTaxa

currentRec.TaxonName = keepTaxonName

currentRec.CommonName = keepCommonName

FLGDOINGBUMPUPTAXA = False

ElseIf FLGDOINGBUMPUPTAXA And FLGARMBUMPUPTAXA Then

'STILL TRYING TAXA

OldTaxa = 9999

Method = 4

GoTo STARTTAXA

ElseIf FLGARMBUMPUPTAXA And Not FLGDOINGBUMPUPTAXA Then

FLGDOINGBUMPUPTAXA = True

keepTaxa = currentRec.TaxonKey

keepTaxonName = currentRec.TaxonName

keepCommonName = currentRec.CommonName

OldTaxa = 9999

Method = 4

GoTo STARTTAXA

End If

Method = 0

FLGARMBUMPUPTAXA = False 'method used up

'method to reverse disaggregated taxa to the original one

If FLGDOINGREVERSEDISAG Then

'reversing taxa failed

'reverse taxa to the original

CurrentTaxon = keepTaxa

currentRec.TaxonKey = keepTaxa

currentRec.TaxonName = keepTaxonName

currentRec.CommonName = keepCommonName

FLGDOINGREVERSEDISAG = False

ElseIf FLGARMREVERSEDISAG And Not FLGDOINGREVERSEDISAG And currentRec.TaxonKey <> currentRec.OriginalTaxon Then

FLGDOINGREVERSEDISAG = True

keepTaxa = currentRec.TaxonKey

keepTaxonName = currentRec.TaxonName

keepCommonName = currentRec.CommonName

CurrentTaxon = currentRec.OriginalTaxon

currentRec.TaxonKey = CurrentTaxon

OldTaxa = 9999

Method = 5

GoTo STARTTAXA

End If

Method = 0

FLGARMREVERSEDISAG = False 'method used up

'method to assign the residual to 'marine animal' with global distribution

If FLGDOINGANIMAL Then

'reversing taxa failed

'reverse taxa to the original

CurrentTaxon = keepTaxa

currentRec.TaxonKey = keepTaxa

currentRec.TaxonName = keepTaxonName

currentRec.CommonName = keepCommonName

FLGDOINGANIMAL = False

ElseIf FLGARMANIMAL And Not FLGDOINGANIMAL And currentRec.TaxonKey <> 100000 Then

FLGDOINGANIMAL = True

keepTaxa = currentRec.TaxonKey

keepTaxonName = currentRec.TaxonName

keepCommonName = currentRec.CommonName

CurrentTaxon = 100000

currentRec.TaxonKey = 100000

OldTaxa = 9999

Method = 6

GoTo STARTTAXA

End If

Method = 0

FLGARMANIMAL = False 'method used up

'FINALLY GIVE UP AND WRITE PROBLEM FILE !!!!!!!!!!!!!!!!!!!!!!!!!!!!!!!!!!!!!!!!!!!!!!!!!!!!!!!!!!!!!

'DOCUMENT METHODS TRIED?

''No catch allocated

writeProblemfile(currentRec, NewCountry, UseYear, SecondPath, CATCHparas)

Else

'SUCCESS

TotalTonnes = TotalTonnes + currentRec.Catch

YrTonOK = YrTonOK + currentRec.Catch

CountOK = CountOK + 1

YrCountOK = YrCountOK + 1

If flgWriteSuccess Then

writeSuccessfile(currentRec, NewCountry, UseYear, SuccessFile)

End If

End If

'=============================================================================================================

'=================================================================================================

nextrec:

'********************** WRITE THE PROGRESS AND STATUS DISPLAYS INCLUDING CALCULATION OF TIME LEFT?? *********************************************

'''REPORT PROGRESS **************************************************************************

errorSpot = "Write Progess"

prog.progressDialog(frm, CJobText, currentRec.Year, Direction)

If (TotalTonnesNotAllo + TotalTonnes) > 0 Then 'some allocated successfully

percentWeightError = String.Format("{0:n3}", (TotalTonnesNotAllo / (TotalTonnesNotAllo + TotalTonnes)) * 100) & "%"

Else

percentWeightError = String.Format(0, "-999") & "%"

End If

If i Mod 1000 = 0 Then

If frm.chkProblems.Checked = False Then swAlloc.Flush()

GC.Collect()

End If

frm.ProgressBar1.Value = i

System.Windows.Forms.Application.DoEvents()

skiprec:

Next i 'next CATCH3 record '@@@@@@@@@@@@@@@@@@@@@@@@@@@@@@@@@@@@@@@@@@@@@@@@@@@@@@@@@@@@@@@@@@@@@@@@@@@@@@@@@@@@@@@@@@@@@@@@@

If swAlloc Is Nothing = False Then If frm.chkProblems.Checked = False Then swAlloc.Close()

prog.progressWrite(CATCHparas.pathC, OldYear, Counterr, CountOK, TotalProcessed, TotalTonnes, TotalTonnesNotAllo, i)

frm.ProgressBar1.Visible = False

'Catch ex As System.Exception

' MsgBox(ex.Message & " : in " & errorSpot, MsgBoxStyle.Critical, "Error")

' frm.placeholder.Text = "Error"

Finally

End Try

iniInfo = Nothing

If flgUseCGroups Then

CurrentCJob = CurrentCJob + 1

If CurrentCJob <= CJob Then

CJobText = CStr(CJobList(CurrentCJob))

OldYear = 999

Counterr = 0

CountOK = 0

TotalProcessed = 0

TotalTonnes = 0

TotalTonnesNotAllo = 0

pickSQL = frmAllo.GetAlloSQL()

''goto GetALLOCsql and rebuild sql that gets data records (with next CGroup)

OldYear = 0

swAlloc = Nothing

GoTo CJobStart

Else

frm.placeholder.Text = "Finished " & Now

End If

Else

frm.placeholder.Text = "Finished " & Now

End If

frm.Noticefrm(1, "")

cntry.Dispose()

swAlloc = Nothing

'tg_Access = Nothing

ComProc = Nothing

CellData = Nothing

If taxaTable Is Nothing = False Then taxaTable.Clear() : taxaTable = Nothing

If reflagTable Is Nothing = False Then reflagTable.Clear() : reflagTable = Nothing

If recCatch Is Nothing = False Then recCatch.Clear() : recCatch = Nothing

'if result files are to be checked afterward for their contents - see report in txt folder

If frm.chkCheckResults.Checked Then

ret = CheckResults(frm, CATCHparas.minYear, CATCHparas.maxYear, CATCHparas)

End If

frm = Nothing

prog = Nothing

'iniInfo = Nothing

GC.Collect()

Windows.Forms.Cursor.Current = Windows.Forms.Cursors.Default

End Function

**6. Post-Processing**

Public Function quotasAdjustment(ByRef frm As frmQuotas) As Boolean

Dim cCodes As countryCodes

Dim quotas As New DataTable

Dim i, j, yr As Long

Dim fisherCode As Integer

Dim EEZcodes() As Integer

Dim taxaStr As String

Dim errorCount As Long = 0

Dim checkedYears As String

Dim startTime As DateTime

Dim tmSpn As TimeSpan

Dim catchTotals As New DataTable

Dim eezTotals As New DataTable

Try

qFrm = frm

'connect to SQL server

curconn = comProc.SQL_ServerConnection_SQLclient(frm.txtUser.Text, frm.txtPwd.Text, frm.cmboServers.Text, frm.cmboDBase.Text)

If curconn Is Nothing = True Then Throw New SystemException("Connection Error: Procedure Aborted")

'build new command object

If startNewCommand() = False Then Exit Function

'fill country codes class

cCodes = New countryCodes(curconn, False)

'fill quota table

quotas = comProc.getDataTable_SQLserver(Me.accessSQL, curconn, False)

'get a delimited string of years that are check in the check box

checkedYears = Me.getCheckedYears

'set progres bar

set_progressBar(quotas.Rows.Count - 1)

'set up outputfile

pathLog = BaseDirectory & "\QuotaAdjustments_" & Trim(Now.Year.ToString) & String.Format(Now.Month, "00") & String.Format(Now.Day, "00") & ".csv"

If qFrm.chkLog.Checked Then If write2file(pathLog, "Source, SourceID, Year, Fisher, EEZ, TargetGrpNum, TaxonKey, TaxonKeyIndiv, Quota, AdjIN, numAdjIN, AdjOUT, numAdjOUT", True) = False Then Throw New System.Exception

'start timing

startTime = Now

'loop through quota records

For i = 0 To quotas.Rows.Count - 1

'If i = 257 Then Stop

' find out if using admin or partition country in quotas

If Me.setRecordValues(quotas.Rows(i), cCodes) Then

'loop through years

For yr = Me.startYr To Me.endYr

'make sure year is one of the checked ones checkedYears = "allYears" Or

If checkedYears.IndexOf(Trim(Str(yr))) >= 0 Then

'write to form telling of progress

qFrm.statusQuotaPan1.Text = "Record " & (i + 1) & " (" & Me.startYr & " to " & Me.endYr & " : doing - " & yr & ") :: " & errorCount & " Errors overall"

'get totals for year in EEZ for country fishing

eezTotals = comProc.getDataTable_SQLserver(Me.buildCatchTotalSQL(yr, True), curconn, False)

'check to make sure catch tonnage is not already below quota

If checkTotals(eezTotals) = True Then

'get the taxonkeys in a delimited string

taxaStr = Me.getDelimitedValues_DataTable(eezTotals, "Taxonkey")

'get totals for year for country fishing only for taxa returned in eezTotals

catchTotals = comProc.getDataTable_SQLserver(Me.buildCatchTotalSQL(yr, False, taxaStr), curconn, False)

'calc adjustIN and adjustOUT and adjust relevant records

If Me.makeAdjustments(catchTotals, eezTotals, yr) = False Then

comProc.ErrorLog(LogFile, Str(i) & "," & Str(yr) & "," & Str(Me.fisherCode) & "," & Me.getDelimitedArray(Me.EEZcode, ";") & "," & Str(Me.tgtGrpSum) & "," & Str(Me.taxonkey) & "," & Str(Me.quotaTonnes) & "," & Me.source & "," & Str(Me.ID), LogFields) '"RecID, Year, Fisher, EEZ, TargetGrpNum, TaxonKey, Quota, Source, SourceID"

errorCount += 1

End If

catchTotals.Clear()

System.Windows.Forms.Application.DoEvents()

End If '-- check EEZ totals

eezTotals.Clear()

End If '-- checked years

Next yr

End If '-- set record values

qFrm.prgBar.PerformStep()

System.Windows.Forms.Application.DoEvents()

Next i '-- next record row

tmSpn = Now.Subtract(startTime)

qFrm.statusQuotaPan1.Text = "Fishished in " & String.Format(tmSpn.TotalHours, "0") & " hours and " & String.Format(tmSpn.Minutes, "0") & " minutes : " & errorCount & " Errors"

Return True

Catch ex As System.Exception

MsgBox("quotasAdjustment Function: " & ex.Message, MsgBoxStyle.Critical)

comProc.ErrorLog(LogFile, Str(i) & "," & Str(yr) & "," & Str(Me.fisherCode) & "," & Me.getDelimitedArray(Me.EEZcode, ";") & "," & Str(Me.tgtGrpSum) & "," & Str(Me.taxonkey) & "," & Str(Me.quotaTonnes) & "," & Me.source & "," & Str(Me.ID), LogFields)

qFrm.statusQuotaPan1.Text = "Quota adjustment aborted"

Return False

Finally

qFrm.lblAdj.Text = ""

If catchTotals Is Nothing = False Then catchTotals.Clear()

If eezTotals Is Nothing = False Then eezTotals.Clear()

If quotas Is Nothing = False Then quotas.Dispose()

If cCodes Is Nothing = False Then cCodes.Dispose()

If cmd Is Nothing = False Then cmd.Dispose()

If curconn Is Nothing = False Then curconn.Dispose()

'If CATCHParas Is Nothing = False Then CATCHParas.__dtor()

qFrm.prgBar.Visible = False

GC.Collect()

End Try

End Function

Public Function Gear(ByVal formAllo As frmAllo, ByRef CATCHparas As CATCHvariables.Globals) As Boolean

'Tranfer of functionality from VB6 to .net

'Program to use Table of Gear associations with taxa/country/year/FAO

'and produce a gear association with each catch record of allocated catch (in Catch table)

'Requires:

' Catch allocated cell catch

' CellData data on cell areas etc - much match that used for allocation

' TaxonNom Taxonomic association table

'

'CATCH + CELLDATA Table --> CATCHSUM Table

'CATCHSUM + TaxonNom --> GEARCATCHSUM

'CATCHSUM + TaxonNom + GEARTABLE --> Populated GEARCATCHSUM

'Populated GEARCATCHSUM --> GEARTOSQL (Used in Summaries etc)

'Produces:

'CatchSum - Summary of catch taken from cell allocated data to guide gear association

'GearCatchSum - Summary of catch using CatchSum to guide gear association - working table

'CatchGear - breakdown of catch by gear

'GeartoSQL - Main product used in subsequent summaries

'RW Oct 2007 NOTE: this whole routine takes about 6-8 hours to run and displays little until finished

Dim NumRec As Long

frm = formAllo

frm.TabControl1.SelectedIndex = 3

'Dim logID As String = Trim(Now.Year.ToString) & String.Format(Now.Month, "00") & String.Format(Now.Day, "00")

'ini variables

'Dim iniInfo As New iniFile(System.AppDomain.CurrentDomain.BaseDirectory() & "\ini\CATCH.ini")

'Dim iniSection As String = "CATCHDATA"

'CATCHparas = New CATCHvariables.Globals

'CATCHparas.setGlobalDefaults()

Dim strSQL As String

Dim curconn As ADODB.Connection

Dim rst As ADODB.Recordset

Dim rst2 As ADODB.Recordset

Dim i As Long

Dim MGear(20) As String

Dim MQualPro(20) As Double

Dim MQual(20) As Double

Dim ans As Long

Dim MaxGears As Long

Dim GearM() As Long

Dim ProM() As Double

Dim flgMakeCatchSum As Boolean

Dim flgMakeGearCatchSum As Boolean

Dim flgFillGearCatchSum As Boolean

Dim flgMakeGeartoSQL As Boolean

Dim flgMakeCatchGear As Boolean

Dim flgExists As Boolean

Dim Ret As Boolean = False

Dim DatabaseName As String

Dim IYear As Long = 0

Dim StartYear As Long = 0

Dim EndYear As Long = 0

'FUNCTION CONTROL - SOME STAGES MIGHT NOT HAVE TO BE DONE

flgMakeCatchSum = True 'if True make CatchSum - a general catch summary from the allocated cell Catch Table - slow

flgMakeGearCatchSum = True 'if True make GearCatchSum from CatchSum -

flgFillGearCatchSum = True 'if True fill in Gear Associations in GearCatchSum - a very long process

flgMakeGeartoSQL = True 'if True create GeartoSQL a flattened version of populated GearSum - quick

flgMakeCatchGear = True 'if True make CatchGear - cell table of gear catch - very slow

Windows.Forms.Cursor.Current = System.Windows.Forms.Cursors.WaitCursor

comProc = New Common

'CATCHparas = New CATCHvariables.Globals

ComputerName = comProc.GetComputerName.ToUpper

DatabaseName = CATCHparas.CatchDatabase

StartYear = CATCHparas.minYear

EndYear = CATCHparas.maxYear

MaxGears = 5 'maximum number of gears that can be assigned in one record to a year/country/place and taxon

ReDim GearM(MaxGears)

ReDim ProM(MaxGears)

'set up data connection ===========================================================================================================

curconn = New ADODB.Connection

With curconn

.ConnectionTimeout = 30

.Provider = "SQLNCLI10"

.Properties("Data Source").Value = "(local)"

.Properties("Initial Catalog").Value = SQLDatabase

.Properties("User ID").Value = "rawatson"

.Properties("Password").Value = "CATCH"

.CommandTimeout = 0

.Open()

End With

'GoTo TableMakeCatchGear

'GoTo IndexCatchGear

Ret = ShrinkDatabase(frmAllo, "CATCH")

frm.Noticefrm(2, "Make CatchGear...")

'CHECK TO SEE IF Geartable

strSQL = "SELECT name FROM sysobjects WHERE xtype = 'U' AND name = 'GearTable'"

rst = New ADODB.Recordset

rst.Open(strSQL, curconn)

flgExists = False

Do Until rst.EOF

flgExists = True

rst.MoveNext()

Loop

If Not flgExists Then

'warn and stop

frm.Noticefrm(1, "GearTable missing")

GoTo endgear

End If

flgExists = False

'CHECK TO SEE IF CATCHSUM EXISTS AND ASK TO MAKE IT IF IT DOES NOT

strSQL = "SELECT name FROM sysobjects WHERE xtype = 'U' AND name = 'CatchSum'"

rst = New ADODB.Recordset

rst.Open(strSQL, curconn)

flgExists = False

Do Until rst.EOF

flgExists = True

rst.MoveNext()

Loop

If flgMakeCatchSum = False And flgExists = False Then

'no choice table must be made

flgMakeCatchSum = True

End If

'=================================================================================================================================

If flgMakeCatchSum = True Then

'Needs CatchSum to work which it updates

'Creates CatchSum from Catch and CellData (must have area and FAO fields)

'a slow procedure

'drop CatchSum if it exists before remaking

Try

strSQL = "drop TABLE Catchsum"

rst = New ADODB.Recordset

rst.Open(strSQL, curconn)

Catch ex As System.Exception

End Try

frm.Noticefrm(1, "Creating CatchSum...")

' Make the CatchSum table

strSQL = "CREATE TABLE [dbo].[CatchSum] "

strSQL = strSQL + "( IYear INT Not Null, "

strSQL = strSQL + " Taxonkey INT Not Null, "

strSQL = strSQL + " CNumber INT Not Null, "

strSQL = strSQL + " FAO INT Not Null, "

strSQL = strSQL + " [Catch] FLOAT "

strSQL = strSQL + " ) "

rst = New ADODB.Recordset

rst.Open(strSQL, curconn)

strSQL = "INSERT INTO CatchSum (Taxonkey, FAO, CNumber, IYear, [Catch] ) "

strSQL = strSQL + "SELECT [Catch].Taxonkey, World.FAO, [Catch].CNumber, [Catch].IYear, SUM([Catch].CatchRate * World.Area) AS [Catch] "

strSQL = strSQL + "FROM [Catch] INNER JOIN World ON [Catch].Cell = World.Seq "

strSQL = strSQL + "GROUP BY [Catch].IYear, [Catch].CNumber, World.FAO, [Catch].Taxonkey "

strSQL = strSQL + "ORDER BY [Catch].IYear, [Catch].CNumber, World.FAO, [Catch].Taxonkey "

rst = New ADODB.Recordset

rst.Open(strSQL, curconn)

'build primary key index

strSQL = "ALTER TABLE CatchSum ADD CONSTRAINT PK_CatchSum PRIMARY KEY CLUSTERED ( IYear, Taxonkey, CNumber, FAO)"

rst = New ADODB.Recordset

rst.Open(strSQL, curconn)

End If 'make CatcSum general summary of catch from cell catch in Catch table (to make sure all combinations used for gear known)

'=================================================================================================================================

'==================================================================================================================================

If flgMakeGearCatchSum = True Then

'Make the GearCatchSum Table from CatchSum and add gear associated.

'drop GearCatchSum if it exists before remaking

Try

strSQL = "drop Table GearCatchSum"

rst = New ADODB.Recordset

rst.Open(strSQL, curconn)

Catch ex As System.Exception

End Try

strSQL = "SELECT TaxonNom.SuperTarget, TaxonNom.TargetGrpNum, CatchSum.Taxonkey, CatchSum.FAO, CatchSum.CNumber, CatchSum.IYear, "

strSQL = strSQL + "CatchSum.Catch INTO GearCatchSum "

strSQL = strSQL + "FROM CatchSum INNER JOIN TaxonNom ON CatchSum.Taxonkey = TaxonNom.TaxonKey"

rst = New ADODB.Recordset

rst.Open(strSQL, curconn)

For i = 1 To 5

strSQL = "Alter table GearCatchSum "

strSQL = strSQL + Constants.vbLf + Constants.vbCr 'note needs linefeed/cr to be parsed properly

strSQL = strSQL + "add Gear" + CStr(i) + " int "

rst = New ADODB.Recordset

rst.Open(strSQL, curconn)

strSQL = "Alter table GearCatchSum "

strSQL = strSQL + Constants.vbLf + Constants.vbCr

strSQL = strSQL + "add Prop" + CStr(i) + " float null "

rst = New ADODB.Recordset

rst.Open(strSQL, curconn)

Next i

strSQL = "Alter table GearCatchSum "

strSQL = strSQL + Constants.vbLf + Constants.vbCr

strSQL = strSQL + "add Pedigree nvarchar(50) null "

rst = New ADODB.Recordset

rst.Open(strSQL, curconn)

strSQL = "CREATE INDEX " + "IndYear ON GearCatchSum(IYear)"

rst = New ADODB.Recordset

rst.Open(strSQL, curconn)

strSQL = "CREATE INDEX " + "IndTG ON GearCatchSum(TargetGrpNum)"

rst = New ADODB.Recordset

rst.Open(strSQL, curconn)

strSQL = "CREATE INDEX " + "IndCNumber ON GearCatchSum(CNumber)"

rst = New ADODB.Recordset

rst.Open(strSQL, curconn)

strSQL = "CREATE INDEX " + "IndTaxon ON GearCatchSum(Taxonkey)"

rst = New ADODB.Recordset

rst.Open(strSQL, curconn)

strSQL = "CREATE INDEX " + "IndSTG ON GearCatchSum(SuperTarget)"

rst = New ADODB.Recordset

rst.Open(strSQL, curconn)

strSQL = "CREATE INDEX " + "IndFAO ON GearCatchSum(FAO)"

rst = New ADODB.Recordset

rst.Open(strSQL, curconn)

Ret = ShrinkDatabase(frmAllo, "CATCH")

frm.Noticefrm(2, "Make CatchGear...")

End If 'end of making GearCatchSum Table

'===============================================================================================================================

If flgFillGearCatchSum = True Then

'Check that there are no nulls in gear proportion fields.. make them 0

strSQL = "Update GearTable Set GearTable.Gear1B = 0 where GearTable.Gear1B is Null"

rst = New ADODB.Recordset

rst.Open(strSQL, curconn)

strSQL = "Update GearTable Set GearTable.Gear2B = 0 where GearTable.Gear2B is Null"

rst = New ADODB.Recordset

rst.Open(strSQL, curconn)

strSQL = "Update GearTable Set GearTable.Gear3B = 0 where GearTable.Gear3B is Null"

rst = New ADODB.Recordset

rst.Open(strSQL, curconn)

strSQL = "Update GearTable Set GearTable.Gear4B = 0 where GearTable.Gear4B is Null"

rst = New ADODB.Recordset

rst.Open(strSQL, curconn)

strSQL = "Update GearTable Set GearTable.Gear5B = 0 where GearTable.Gear5B is Null"

rst = New ADODB.Recordset

rst.Open(strSQL, curconn)

strSQL = "Update GearTable Set GearTable.ProGear1 = 0 where GearTable.ProGear1 is Null"

rst = New ADODB.Recordset

rst.Open(strSQL, curconn)

strSQL = "Update GearTable Set GearTable.ProGear2 = 0 where GearTable.ProGear2 is Null"

rst = New ADODB.Recordset

rst.Open(strSQL, curconn)

strSQL = "Update GearTable Set GearTable.ProGear3 = 0 where GearTable.ProGear3 is Null"

rst = New ADODB.Recordset

rst.Open(strSQL, curconn)

strSQL = "Update GearTable Set GearTable.ProGear4 = 0 where GearTable.ProGear4 is Null"

rst = New ADODB.Recordset

rst.Open(strSQL, curconn)

strSQL = "Update GearTable Set GearTable.ProGear5 = 0 where GearTable.ProGear5 is Null"

rst = New ADODB.Recordset

rst.Open(strSQL, curconn)

'there is no gear data for TG 10 so we will make that = TG 11

P1:

'=============================================== TG ONLY LEVEL 1 =========================================================

'Get the list of TGs to use from GearCatchSum

Ret = ShrinkDatabase(frmAllo, "CATCH")

frm.Noticefrm(2, "Make CatchGear...")

strSQL = "SELECT TaxonNom.TargetGrpNum, Sum(GearCatchSum.Catch) AS Catch "

strSQL = strSQL + "FROM GearCatchSum INNER JOIN TaxonNom ON GearCatchSum.TaxonKey = TaxonNom.TaxonKey "

strSQL = strSQL + "GROUP BY TaxonNom.TargetGrpNum "

strSQL = strSQL + "ORDER BY TaxonNom.TargetGrpNum "

'Get Records

rst = New ADODB.Recordset

rst.Open(strSQL, curconn)

Do Until rst.EOF

'If rst.Fields!TargetGrpNum.value = 10 Then Stop

frm.Noticefrm(1, "1: TG=" + CStr(rst.Fields!TargetGrpNum.Value))

'Get records for the TG

strSQL = "SELECT TaxonNom.TargetGrpNum, GearTable.Gear1B, GearTable.Gear2B, GearTable.Gear3B, GearTable.Gear4B, GearTable.Gear5B, "

strSQL = strSQL + "GearTable.ProGear1, GearTable.ProGear2, GearTable.ProGear3, GearTable.ProGear4, GearTable.ProGear5, GearTable.Quality "

strSQL = strSQL + "FROM GearTable INNER JOIN TaxonNom ON GearTable.TaxonKey = TaxonNom.TaxonKey "

'NOTE: no data for TG 9 or 10 so far

If rst.Fields!TargetGrpNum.Value = 10 Then 'there is no gear data for TG 10 so we will make that = TG 11

strSQL = strSQL + "WHERE TaxonNom.TargetGrpNum=11 "

ElseIf rst.Fields!TargetGrpNum.Value = 9 Then 'there is no gear data for TG 9 so we will make that = TG 8

strSQL = strSQL + "WHERE TaxonNom.TargetGrpNum=8 "

Else

strSQL = strSQL + "WHERE TaxonNom.TargetGrpNum=" + CStr(rst.Fields!TargetGrpNum.Value)

End If

'Get Gear Recordsrst = New ADODB.Recordset

rst2 = New ADODB.Recordset

rst2.Open(strSQL, curconn)

'If rst2.RecordCount = 0 Then GoTo nextTG

ans = WeightRecords(rst2, MaxGears, GearM, ProM)

If ans = 0 Then GoTo nextTG

'Process results

strSQL = "UPDATE GearCatchSum SET "

strSQL = strSQL + "GearCatchSum.Gear1 =" + CStr(GearM(1)) + ", GearCatchSum.Prop1 =" + CStr(ProM(1)) + ", "

strSQL = strSQL + "GearCatchSum.Gear2 =" + CStr(GearM(2)) + ", GearCatchSum.Prop2 =" + CStr(ProM(2)) + ", "

strSQL = strSQL + "GearCatchSum.Gear3 =" + CStr(GearM(3)) + ", GearCatchSum.Prop3 =" + CStr(ProM(3)) + ", "

strSQL = strSQL + "GearCatchSum.Gear4 =" + CStr(GearM(4)) + ", GearCatchSum.Prop4 =" + CStr(ProM(4)) + ", "

strSQL = strSQL + "GearCatchSum.Gear5 =" + CStr(GearM(5)) + ", GearCatchSum.Prop5 =" + CStr(ProM(5)) + ", "

strSQL = strSQL + "GearCatchSum.Pedigree = '1' "

strSQL = strSQL + "WHERE GearCatchSum.TargetGrpNum=" + CStr(rst.Fields!TargetGrpNum.Value)

'Modify Summary Gear Records

rst2 = New ADODB.Recordset

rst2.Open(strSQL, curconn)

nextTG:

rst.MoveNext()

Loop

'==================================================================================================================================

P2A:

'=============================================== TG AND FAO LEVEL 2A =========================================================

'Get the list of TG x FAO to use from GearCatchSum

Ret = ShrinkDatabase(frmAllo, "CATCH")

frm.Noticefrm(2, "Make CatchGear...")

strSQL = "SELECT GearCatchSum.TargetGrpNum, GearCatchSum.FAO, Sum(GearCatchSum.Catch) AS Catch "

strSQL = strSQL + "from GearCatchSum "

strSQL = strSQL + "GROUP BY GearCatchSum.TargetGrpNum, GearCatchSum.FAO "

strSQL = strSQL + "ORDER BY GearCatchSum.TargetGrpNum, GearCatchSum.FAO "

'Get Records

rst = New ADODB.Recordset

rst.Open(strSQL, curconn)

Do Until rst.EOF

'do until rst.eof

frm.Noticefrm(1, "2A: TG=" + CStr(rst.Fields!TargetGrpNum.Value) + " FAO=" + CStr(rst.Fields!FAO.Value))

'Get records for the TG

strSQL = "SELECT TaxonNom.TargetGrpNum, GearTable.Gear1B, GearTable.Gear2B, GearTable.Gear3B, GearTable.Gear4B, GearTable.Gear5B, "

strSQL = strSQL + "GearTable.ProGear1, GearTable.ProGear2, GearTable.ProGear3, GearTable.ProGear4, GearTable.ProGear5, GearTable.Quality "

strSQL = strSQL + "FROM GearTable INNER JOIN TaxonNom ON GearTable.TaxonKey = TaxonNom.TaxonKey "

'NOTE: no data for TG 9 or 10 so far

If rst.Fields!TargetGrpNum.Value = 10 Then 'there is no gear data for TG 10 so we will make that = TG 11

strSQL = strSQL + "WHERE TaxonNom.TargetGrpNum=11 AND "

ElseIf rst.Fields!TargetGrpNum.Value = 9 Then 'there is no gear data for TG 9 so we will make that = TG 8

strSQL = strSQL + "WHERE TaxonNom.TargetGrpNum=8 AND "

Else

strSQL = strSQL + "WHERE TaxonNom.TargetGrpNum=" + CStr(rst.Fields!TargetGrpNum.Value) + " AND "

End If

strSQL = strSQL + "GearTable.FAO=" + CStr(rst.Fields!FAO.Value)

'Get Gear Records

rst2 = New ADODB.Recordset

rst2.Open(strSQL, curconn)

'rst2.MoveLast()

'If rst2.RecordCount = 0 Then GoTo nextTGFAO '*****************

Try

rst2.MoveFirst()

Catch

GoTo nextTGFAO

End Try

ans = WeightRecords(rst2, MaxGears, GearM, ProM)

If ans = 0 Then GoTo nextTGFAO '******************************

'Process results

strSQL = "UPDATE GearCatchSum SET "

strSQL = strSQL + "GearCatchSum.Gear1 =" + CStr(GearM(1)) + ", GearCatchSum.Prop1 =" + CStr(ProM(1)) + ", "

strSQL = strSQL + "GearCatchSum.Gear2 =" + CStr(GearM(2)) + ", GearCatchSum.Prop2 =" + CStr(ProM(2)) + ", "

strSQL = strSQL + "GearCatchSum.Gear3 =" + CStr(GearM(3)) + ", GearCatchSum.Prop3 =" + CStr(ProM(3)) + ", "

strSQL = strSQL + "GearCatchSum.Gear4 =" + CStr(GearM(4)) + ", GearCatchSum.Prop4 =" + CStr(ProM(4)) + ", "

strSQL = strSQL + "GearCatchSum.Gear5 =" + CStr(GearM(5)) + ", GearCatchSum.Prop5 =" + CStr(ProM(5)) + ", "

strSQL = strSQL + "GearCatchSum.Pedigree = '2A' "

strSQL = strSQL + "WHERE GearCatchSum.TargetGrpNum=" + CStr(rst.Fields!TargetGrpNum.Value) + " and "

strSQL = strSQL + "GearCatchSum.FAO=" + CStr(rst.Fields!FAO.Value)

'Modify Summary Gear Records

rst2 = New ADODB.Recordset

rst2.Open(strSQL, curconn)

nextTGFAO:

rst.MoveNext()

Loop

'==================================================================================================================================

P3A:

'=============================================== TG AND FAO AND COUNTRY LEVEL 3A =========================================================

'Get the list of TG x FAO x COUNTRY to use from GearCatchSum

Ret = ShrinkDatabase(frmAllo, "CATCH")

frm.Noticefrm(2, "Make CatchGear...")

strSQL = "SELECT GearCatchSum.TargetGrpNum, GearCatchSum.FAO, GearCatchSum.CNumber, Sum(GearCatchSum.Catch) AS Catch "

strSQL = strSQL + "from GearCatchSum "

strSQL = strSQL + "GROUP BY GearCatchSum.TargetGrpNum, GearCatchSum.FAO, GearCatchSum.CNumber "

strSQL = strSQL + "ORDER BY GearCatchSum.TargetGrpNum, GearCatchSum.FAO, GearCatchSum.CNumber "

'Get Records

rst = New ADODB.Recordset

rst.Open(strSQL, curconn)

'rst.MoveLast()

'NumRec = rst.RecordCount

'rst.MoveFirst()

Do Until rst.EOF

frm.Noticefrm(1, "3A: TG=" + CStr(rst.Fields!TargetGrpNum.Value) + " FAO=" + CStr(rst.Fields!FAO.Value) + " COUNTRY=" + CStr(rst.Fields!CNumber.Value))

'Get records for the TG

strSQL = "SELECT TaxonNom.TargetGrpNum, GearTable.Gear1B, GearTable.Gear2B, GearTable.Gear3B, GearTable.Gear4B, GearTable.Gear5B, "

strSQL = strSQL + "GearTable.ProGear1, GearTable.ProGear2, GearTable.ProGear3, GearTable.ProGear4, GearTable.ProGear5, GearTable.Quality "

strSQL = strSQL + "FROM GearTable INNER JOIN TaxonNom ON GearTable.TaxonKey = TaxonNom.TaxonKey "

'NOTE: no data for TG 9 or 10 so far

If rst.Fields!TargetGrpNum.Value = 10 Then 'there is no gear data for TG 10 so we will make that = TG 11

strSQL = strSQL + "WHERE TaxonNom.TargetGrpNum=11 AND "

ElseIf rst.Fields!TargetGrpNum.Value = 9 Then 'there is no gear data for TG 9 so we will make that = TG 8

strSQL = strSQL + "WHERE TaxonNom.TargetGrpNum=8 AND "

Else

strSQL = strSQL + "WHERE TaxonNom.TargetGrpNum=" + CStr(rst.Fields!TargetGrpNum.Value) + " AND "

End If

strSQL = strSQL + "GearTable.FAO=" + CStr(rst.Fields!FAO.Value) + " and "

strSQL = strSQL + "GearTable.CNumber=" + CStr(rst.Fields!CNumber.Value)

'Get Gear Records

rst2 = New ADODB.Recordset

rst2.Open(strSQL, curconn)

'rst2.MoveLast()

'If rst2.RecordCount = 0 Then GoTo nextTGFAOCount '*****************

Try

rst2.MoveFirst()

Catch

GoTo nextTGFAOCount

End Try

ans = WeightRecords(rst2, MaxGears, GearM, ProM)

If ans = 0 Then GoTo nextTGFAOCount '******************************

'Process results

strSQL = "UPDATE GearCatchSum SET "

strSQL = strSQL + "GearCatchSum.Gear1 =" + CStr(GearM(1)) + ", GearCatchSum.Prop1 =" + CStr(ProM(1)) + ", "

strSQL = strSQL + "GearCatchSum.Gear2 =" + CStr(GearM(2)) + ", GearCatchSum.Prop2 =" + CStr(ProM(2)) + ", "

strSQL = strSQL + "GearCatchSum.Gear3 =" + CStr(GearM(3)) + ", GearCatchSum.Prop3 =" + CStr(ProM(3)) + ", "

strSQL = strSQL + "GearCatchSum.Gear4 =" + CStr(GearM(4)) + ", GearCatchSum.Prop4 =" + CStr(ProM(4)) + ", "

strSQL = strSQL + "GearCatchSum.Gear5 =" + CStr(GearM(5)) + ", GearCatchSum.Prop5 =" + CStr(ProM(5)) + ", "

strSQL = strSQL + "GearCatchSum.Pedigree = '3A' "

strSQL = strSQL + "WHERE GearCatchSum.TargetGrpNum=" + CStr(rst.Fields!TargetGrpNum.Value) + " and "

strSQL = strSQL + "GearCatchSum.FAO=" + CStr(rst.Fields!FAO.Value) + " and "

strSQL = strSQL + "GearCatchSum.CNumber=" + CStr(rst.Fields!CNumber.Value)

'Modify Summary Gear Records

rst2 = New ADODB.Recordset

rst2.Open(strSQL, curconn)

nextTGFAOCount:

rst.MoveNext()

Loop

'==================================================================================================================================

P4A:

'=============================================== TG AND FAO AND COUNTRY AND YEAR LEVEL 4A =========================================================

'Get the list of TG x FAO x COUNTRY x YEAR to use from GearCatchSum

Ret = ShrinkDatabase(frmAllo, "CATCH")

frm.Noticefrm(2, "Make CatchGear...")

strSQL = "SELECT GearCatchSum.TargetGrpNum, GearCatchSum.FAO, GearCatchSum.CNumber, GearCatchSum.IYear, Sum(GearCatchSum.Catch) AS Catch "

strSQL = strSQL + "from GearCatchSum "

strSQL = strSQL + "GROUP BY GearCatchSum.TargetGrpNum, GearCatchSum.FAO, GearCatchSum.CNumber, GearCatchSum.IYear "

strSQL = strSQL + "ORDER BY GearCatchSum.TargetGrpNum, GearCatchSum.FAO, GearCatchSum.CNumber, GearCatchSum.IYear "

'Get Records

rst = New ADODB.Recordset

rst.Open(strSQL, curconn)

Do Until rst.EOF

frm.Noticefrm(1, "4A: TG=" + CStr(rst.Fields!TargetGrpNum.Value) + " FAO=" + CStr(rst.Fields!FAO.Value) + " COUNTRY=" + CStr(rst.Fields!CNumber.Value) + " YEAR=" + CStr(rst.Fields!IYear.Value))

'Get records for the TG

strSQL = "SELECT TaxonNom.TargetGrpNum, GearTable.Gear1B, GearTable.Gear2B, GearTable.Gear3B, GearTable.Gear4B, GearTable.Gear5B, "

strSQL = strSQL + "GearTable.ProGear1, GearTable.ProGear2, GearTable.ProGear3, GearTable.ProGear4, GearTable.ProGear5, GearTable.Quality "

strSQL = strSQL + "FROM GearTable INNER JOIN TaxonNom ON GearTable.TaxonKey = TaxonNom.TaxonKey "

'NOTE: no data for TG 9 or 10 so far

If rst.Fields!TargetGrpNum.Value = 10 Then 'there is no gear data for TG 10 so we will make that = TG 11

strSQL = strSQL + "WHERE TaxonNom.TargetGrpNum=11 AND "

ElseIf rst.Fields!TargetGrpNum.Value = 9 Then 'there is no gear data for TG 9 so we will make that = TG 8

strSQL = strSQL + "WHERE TaxonNom.TargetGrpNum=8 AND "

Else

strSQL = strSQL + "WHERE TaxonNom.TargetGrpNum=" + CStr(rst.Fields!TargetGrpNum.Value) + " AND "

End If

strSQL = strSQL + "GearTable.FAO=" + CStr(rst.Fields!FAO.Value) + " and "

strSQL = strSQL + "GearTable.CNumber=" + CStr(rst.Fields!CNumber.Value) + " and "

strSQL = strSQL + "GearTable.FirstYear <=" + CStr(rst.Fields!IYear.Value) + " and "

strSQL = strSQL + "GearTable.LastYear >=" + CStr(rst.Fields!IYear.Value) + " and "

strSQL = strSQL + "(GearTable.FirstYear >" + CStr(CATCHparas.minYear) + " or GearTable.LastYear < 9999)"

'Get Gear Records

rst2 = New ADODB.Recordset

rst2.Open(strSQL, curconn)

'rst2.MoveLast()

'If rst2.RecordCount = 0 Then GoTo nextTGFAOCountYr '*****************

Try

rst2.MoveFirst()

Catch

GoTo nextTGFAOCountYr

End Try

ans = WeightRecords(rst2, MaxGears, GearM, ProM)

If ans = 0 Then GoTo nextTGFAOCountYr '******************************

'Process results

strSQL = "UPDATE GearCatchSum SET "

strSQL = strSQL + "GearCatchSum.Gear1 =" + CStr(GearM(1)) + ", GearCatchSum.Prop1 =" + CStr(ProM(1)) + ", "

strSQL = strSQL + "GearCatchSum.Gear2 =" + CStr(GearM(2)) + ", GearCatchSum.Prop2 =" + CStr(ProM(2)) + ", "

strSQL = strSQL + "GearCatchSum.Gear3 =" + CStr(GearM(3)) + ", GearCatchSum.Prop3 =" + CStr(ProM(3)) + ", "

strSQL = strSQL + "GearCatchSum.Gear4 =" + CStr(GearM(4)) + ", GearCatchSum.Prop4 =" + CStr(ProM(4)) + ", "

strSQL = strSQL + "GearCatchSum.Gear5 =" + CStr(GearM(5)) + ", GearCatchSum.Prop5 =" + CStr(ProM(5)) + ", "

strSQL = strSQL + "GearCatchSum.Pedigree = '4A' "

strSQL = strSQL + "WHERE GearCatchSum.TargetGrpNum=" + CStr(rst.Fields!TargetGrpNum.Value) + " and "

strSQL = strSQL + "GearCatchSum.FAO=" + CStr(rst.Fields!FAO.Value) + " and "

strSQL = strSQL + "GearCatchSum.CNumber=" + CStr(rst.Fields!CNumber.Value) + " and "

strSQL = strSQL + "GearCatchSum.IYear=" + CStr(rst.Fields!IYear.Value)

'Modify Summary Gear Records

rst2 = New ADODB.Recordset

rst2.Open(strSQL, curconn)

nextTGFAOCountYr:

rst.MoveNext()

Loop

'==================================================================================================================================

P2B:

'=============================================== TG AND COUNTRY LEVEL 2B =========================================================

'Get the list of TG x COUNTRY to use from GearCatchSum

Ret = ShrinkDatabase(frmAllo, "CATCH")

frm.Noticefrm(2, "Make CatchGear...")

strSQL = "SELECT GearCatchSum.TargetGrpNum, GearCatchSum.CNumber, Sum(GearCatchSum.Catch) AS Catch "

strSQL = strSQL + "from GearCatchSum "

strSQL = strSQL + "where GearCatchSum.Pedigree='1' or GearCatchSum.Pedigree='2A' "

strSQL = strSQL + "GROUP BY GearCatchSum.TargetGrpNum, GearCatchSum.CNumber "

strSQL = strSQL + "ORDER BY GearCatchSum.TargetGrpNum, GearCatchSum.CNumber "

'Get Records

rst = New ADODB.Recordset

rst.Open(strSQL, curconn)

Do Until rst.EOF

frm.Noticefrm(1, "2B: TG=" + CStr(rst.Fields!TargetGrpNum.Value) + " COUNTRY=" + CStr(rst.Fields!CNumber.Value))

'Get records for the TG

strSQL = "SELECT TaxonNom.TargetGrpNum, GearTable.Gear1B, GearTable.Gear2B, GearTable.Gear3B, GearTable.Gear4B, GearTable.Gear5B, "

strSQL = strSQL + "GearTable.ProGear1, GearTable.ProGear2, GearTable.ProGear3, GearTable.ProGear4, GearTable.ProGear5, GearTable.Quality "

strSQL = strSQL + "FROM GearTable INNER JOIN TaxonNom ON GearTable.TaxonKey = TaxonNom.TaxonKey "

'NOTE: no data for TG 9 or 10 so far

If rst.Fields!TargetGrpNum.Value = 10 Then 'there is no gear data for TG 10 so we will make that = TG 11

strSQL = strSQL + "WHERE TaxonNom.TargetGrpNum=11 AND "

ElseIf rst.Fields!TargetGrpNum.Value = 9 Then 'there is no gear data for TG 9 so we will make that = TG 8

strSQL = strSQL + "WHERE TaxonNom.TargetGrpNum=8 AND "

Else

strSQL = strSQL + "WHERE TaxonNom.TargetGrpNum=" + CStr(rst.Fields!TargetGrpNum.Value) + " AND "

End If

strSQL = strSQL + "GearTable.CNumber=" + CStr(rst.Fields!CNumber.Value) + " and "

strSQL = strSQL + "GearTable.Origcountcode<>0 "

'Get Gear Records

rst2 = New ADODB.Recordset

rst2.Open(strSQL, curconn)

'rst2.MoveLast()

'If rst2.RecordCount = 0 Then GoTo nextTGCount '*****************

Try

rst2.MoveFirst()

Catch

GoTo nextTGCount

End Try

ans = WeightRecords(rst2, MaxGears, GearM, ProM)

If ans = 0 Then GoTo nextTGCount '******************************

'Process results

strSQL = "UPDATE GearCatchSum SET "

strSQL = strSQL + "GearCatchSum.Gear1 =" + CStr(GearM(1)) + ", GearCatchSum.Prop1 =" + CStr(ProM(1)) + ", "

strSQL = strSQL + "GearCatchSum.Gear2 =" + CStr(GearM(2)) + ", GearCatchSum.Prop2 =" + CStr(ProM(2)) + ", "

strSQL = strSQL + "GearCatchSum.Gear3 =" + CStr(GearM(3)) + ", GearCatchSum.Prop3 =" + CStr(ProM(3)) + ", "

strSQL = strSQL + "GearCatchSum.Gear4 =" + CStr(GearM(4)) + ", GearCatchSum.Prop4 =" + CStr(ProM(4)) + ", "

strSQL = strSQL + "GearCatchSum.Gear5 =" + CStr(GearM(5)) + ", GearCatchSum.Prop5 =" + CStr(ProM(5)) + ", "

strSQL = strSQL + "GearCatchSum.Pedigree = '2B' "

strSQL = strSQL + "WHERE GearCatchSum.TargetGrpNum=" + CStr(rst.Fields!TargetGrpNum.Value) + " and "

strSQL = strSQL + "GearCatchSum.CNumber=" + CStr(rst.Fields!CNumber.Value)

'Modify Summary Gear Records

rst2 = New ADODB.Recordset

rst2.Open(strSQL, curconn)

nextTGCount:

rst.MoveNext()

Loop

'==================================================================================================================================

P3B:

'=============================================== TG AND COUNTRY AND YEAR LEVEL 3B =========================================================

'Get the list of TG x COUNTRY x YEAR to use from GearCatchSum

Ret = ShrinkDatabase(frmAllo, "CATCH")

frm.Noticefrm(2, "Make CatchGear...")

strSQL = "SELECT GearCatchSum.TargetGrpNum, GearCatchSum.CNumber, GearCatchSum.IYear, Sum(GearCatchSum.Catch) AS Catch "

strSQL = strSQL + "from GearCatchSum "

strSQL = strSQL + "where GearCatchSum.Pedigree='2B' "

strSQL = strSQL + "GROUP BY GearCatchSum.TargetGrpNum, GearCatchSum.CNumber, GearCatchSum.IYear "

strSQL = strSQL + "ORDER BY GearCatchSum.TargetGrpNum, GearCatchSum.CNumber, GearCatchSum.IYear;"

'Get Records

rst = New ADODB.Recordset

rst.Open(strSQL, curconn)

Do Until rst.EOF

frm.Noticefrm(1, "3B: TG=" + CStr(rst.Fields!TargetGrpNum.Value) + " COUNTRY=" + CStr(rst.Fields!CNumber.Value) + " YEAR=" + CStr(rst.Fields!IYear.Value))

'Get records for the TG

strSQL = "SELECT TaxonNom.TargetGrpNum, GearTable.Gear1B, GearTable.Gear2B, GearTable.Gear3B, GearTable.Gear4B, GearTable.Gear5B, "

strSQL = strSQL + "GearTable.ProGear1, GearTable.ProGear2, GearTable.ProGear3, GearTable.ProGear4, GearTable.ProGear5, GearTable.Quality "

strSQL = strSQL + "FROM GearTable INNER JOIN TaxonNom ON GearTable.TaxonKey = TaxonNom.TaxonKey "

'NOTE: no data for TG 9 or 10 so far

If rst.Fields!TargetGrpNum.Value = 10 Then 'there is no gear data for TG 10 so we will make that = TG 11

strSQL = strSQL + "WHERE TaxonNom.TargetGrpNum=11 AND "

ElseIf rst.Fields!TargetGrpNum.Value = 9 Then 'there is no gear data for TG 9 so we will make that = TG 8

strSQL = strSQL + "WHERE TaxonNom.TargetGrpNum=8 AND "

Else

strSQL = strSQL + "WHERE TaxonNom.TargetGrpNum=" + CStr(rst.Fields!TargetGrpNum.Value) + " AND "

End If

strSQL = strSQL + "GearTable.CNumber=" + CStr(rst.Fields!CNumber.Value) + " and "

strSQL = strSQL + "GearTable.Origcountcode<>0 and "

strSQL = strSQL + "GearTable.FirstYear <=" + CStr(rst.Fields!IYear.Value) + " and "

strSQL = strSQL + "GearTable.LastYear >=" + CStr(rst.Fields!IYear.Value) + " and "

strSQL = strSQL + "(GearTable.FirstYear >" + CStr(CATCHparas.minYear) + " or GearTable.LastYear < 9999)"

'Get Gear Records

rst2 = New ADODB.Recordset

rst2.Open(strSQL, curconn)

'rst2.MoveLast()

'If rst2.RecordCount = 0 Then GoTo nextTGCountYr '*****************

Try

rst2.MoveFirst()

Catch

GoTo nextTGCountYr

End Try

ans = WeightRecords(rst2, MaxGears, GearM, ProM)

If ans = 0 Then GoTo nextTGCountYr '******************************

'Process results

strSQL = "UPDATE GearCatchSum SET "

strSQL = strSQL + "GearCatchSum.Gear1 =" + CStr(GearM(1)) + ", GearCatchSum.Prop1 =" + CStr(ProM(1)) + ", "

strSQL = strSQL + "GearCatchSum.Gear2 =" + CStr(GearM(2)) + ", GearCatchSum.Prop2 =" + CStr(ProM(2)) + ", "

strSQL = strSQL + "GearCatchSum.Gear3 =" + CStr(GearM(3)) + ", GearCatchSum.Prop3 =" + CStr(ProM(3)) + ", "

strSQL = strSQL + "GearCatchSum.Gear4 =" + CStr(GearM(4)) + ", GearCatchSum.Prop4 =" + CStr(ProM(4)) + ", "

strSQL = strSQL + "GearCatchSum.Gear5 =" + CStr(GearM(5)) + ", GearCatchSum.Prop5 =" + CStr(ProM(5)) + ", "

strSQL = strSQL + "GearCatchSum.Pedigree = '2B' "

strSQL = strSQL + "WHERE GearCatchSum.TargetGrpNum=" + CStr(rst.Fields!TargetGrpNum.Value) + " and "

strSQL = strSQL + "GearCatchSum.CNumber=" + CStr(rst.Fields!CNumber.Value) + " and "

strSQL = strSQL + "GearCatchSum.Iyear=" + CStr(rst.Fields!IYear.Value)

'Modify Summary Gear Records

rst2 = New ADODB.Recordset

rst2.Open(strSQL, curconn)

nextTGCountYr:

rst.MoveNext()

Loop

'==================================================================================================================================

P3C:

'=============================================== TG AND FAO AND YEAR LEVEL 3C =========================================================

'Get the list of TG x FAO x YEAR to use from GearCatchSum

Ret = ShrinkDatabase(frmAllo, "CATCH")

frm.Noticefrm(2, "Make CatchGear...")

strSQL = "SELECT GearCatchSum.TargetGrpNum, GearCatchSum.FAO, GearCatchSum.IYear, Sum(GearCatchSum.Catch) AS Catch "

strSQL = strSQL + "from GearCatchSum "

strSQL = strSQL + "where GearCatchSum.Pedigree='2B' "

strSQL = strSQL + "GROUP BY GearCatchSum.TargetGrpNum, GearCatchSum.FAO, GearCatchSum.IYear "

strSQL = strSQL + "ORDER BY GearCatchSum.TargetGrpNum, GearCatchSum.FAO, GearCatchSum.IYear;"

'Get Records

rst = New ADODB.Recordset

rst.Open(strSQL, curconn)

'rst.MoveLast()

'NumRec = rst.RecordCount

'rst.MoveFirst()

Do Until rst.EOF

frm.Noticefrm(1, "3C: TG=" + CStr(rst.Fields!TargetGrpNum.Value) + " FAO=" + CStr(rst.Fields!FAO.Value) + " YEAR=" + CStr(rst.Fields!IYear.Value))

'Get records for the TG

strSQL = "SELECT TaxonNom.TargetGrpNum, GearTable.Gear1B, GearTable.Gear2B, GearTable.Gear3B, GearTable.Gear4B, GearTable.Gear5B, "

strSQL = strSQL + "GearTable.ProGear1, GearTable.ProGear2, GearTable.ProGear3, GearTable.ProGear4, GearTable.ProGear5, GearTable.Quality "

strSQL = strSQL + "FROM GearTable INNER JOIN TaxonNom ON GearTable.TaxonKey = TaxonNom.TaxonKey "

'NOTE: no data for TG 9 or 10 so far

If rst.Fields!TargetGrpNum.Value = 10 Then 'there is no gear data for TG 10 so we will make that = TG 11

strSQL = strSQL + "WHERE TaxonNom.TargetGrpNum=11 AND "

ElseIf rst.Fields!TargetGrpNum.Value = 9 Then 'there is no gear data for TG 9 so we will make that = TG 8

strSQL = strSQL + "WHERE TaxonNom.TargetGrpNum=8 AND "

Else

strSQL = strSQL + "WHERE TaxonNom.TargetGrpNum=" + CStr(rst.Fields!TargetGrpNum.Value) + " AND "

End If

strSQL = strSQL + "GearTable.FAO=" + CStr(rst.Fields!FAO.Value) + " and "

strSQL = strSQL + "GearTable.FirstYear <=" + CStr(rst.Fields!IYear.Value) + " and "

strSQL = strSQL + "GearTable.LastYear >=" + CStr(rst.Fields!IYear.Value) + " and "

strSQL = strSQL + "(GearTable.FirstYear >" + CStr(CATCHparas.minYear) + " or GearTable.LastYear < 9999)"

'Get Gear Records

rst2 = New ADODB.Recordset

rst2.Open(strSQL, curconn)

'rst2.MoveLast()

'If rst2.RecordCount = 0 Then GoTo nextTGFAOYr '*****************

Try

rst2.MoveFirst()

Catch

GoTo nextTGFAOYr

End Try

ans = WeightRecords(rst2, MaxGears, GearM, ProM)

If ans = 0 Then GoTo nextTGFAOYr '******************************

'Process results

strSQL = "UPDATE GearCatchSum SET "

strSQL = strSQL + "GearCatchSum.Gear1 =" + CStr(GearM(1)) + ", GearCatchSum.Prop1 =" + CStr(ProM(1)) + ", "

strSQL = strSQL + "GearCatchSum.Gear2 =" + CStr(GearM(2)) + ", GearCatchSum.Prop2 =" + CStr(ProM(2)) + ", "

strSQL = strSQL + "GearCatchSum.Gear3 =" + CStr(GearM(3)) + ", GearCatchSum.Prop3 =" + CStr(ProM(3)) + ", "

strSQL = strSQL + "GearCatchSum.Gear4 =" + CStr(GearM(4)) + ", GearCatchSum.Prop4 =" + CStr(ProM(4)) + ", "

strSQL = strSQL + "GearCatchSum.Gear5 =" + CStr(GearM(5)) + ", GearCatchSum.Prop5 =" + CStr(ProM(5)) + ", "

strSQL = strSQL + "GearCatchSum.Pedigree = '2A' "

strSQL = strSQL + "WHERE GearCatchSum.TargetGrpNum=" + CStr(rst.Fields!TargetGrpNum.Value) + " and "

strSQL = strSQL + "GearCatchSum.FAO=" + CStr(rst.Fields!FAO.Value) + " and "

strSQL = strSQL + "GearCatchSum.Iyear=" + CStr(rst.Fields!IYear.Value)

'Modify Summary Gear Records

rst2 = New ADODB.Recordset

rst2.Open(strSQL, curconn)

nextTGFAOYr:

rst.MoveNext()

Loop

'==================================================================================================================================

P5:

'=============================================== Tax LEVEL 5 =========================================================

'Get the list of Taxonkey to use from GearCatchSum

Ret = ShrinkDatabase(frmAllo, "CATCH")

frm.Noticefrm(2, "Make CatchGear...")

strSQL = "SELECT GearCatchSum.Taxonkey, Sum(GearCatchSum.Catch) AS Catch "

strSQL = strSQL + "from GearCatchSum "

strSQL = strSQL + "where (GearCatchSum.Pedigree='1' or GearCatchSum.Pedigree='2A' or GearCatchSum.Pedigree='2B') "

strSQL = strSQL + "GROUP BY GearCatchSum.Taxonkey "

strSQL = strSQL + "ORDER BY GearCatchSum.Taxonkey;"

'Get Records

rst = New ADODB.Recordset

rst.Open(strSQL, curconn)

'rst.MoveLast()

' NumRec = rst.RecordCount

'Try

rst.MoveFirst()

'Catch

'End Try

Do Until rst.EOF

frm.Noticefrm(1, "5: Taxon=" + CStr(rst.Fields!TaxonKey.Value))

'Get records for the Tax

strSQL = "SELECT GearTable.Taxonkey, GearTable.Gear1B, GearTable.Gear2B, GearTable.Gear3B, GearTable.Gear4B, GearTable.Gear5B, "

strSQL = strSQL + "GearTable.ProGear1, GearTable.ProGear2, GearTable.ProGear3, GearTable.ProGear4, GearTable.ProGear5, GearTable.Quality "

strSQL = strSQL + "FROM GearTable "

strSQL = strSQL + "WHERE GearTable.Taxonkey=" + CStr(rst.Fields!TaxonKey.Value)

'Get Gear Records

rst2 = New ADODB.Recordset

rst2.Open(strSQL, curconn)

'rst2.MoveLast()

'If rst2.RecordCount = 0 Then GoTo nextTax '*****************

Try

rst2.MoveFirst()

Catch

GoTo nextTax

End Try

ans = WeightRecords(rst2, MaxGears, GearM, ProM)

If ans = 0 Then GoTo nextTax '******************************

'Process results

strSQL = "UPDATE GearCatchSum SET "

strSQL = strSQL + "GearCatchSum.Gear1 =" + CStr(GearM(1)) + ", GearCatchSum.Prop1 =" + CStr(ProM(1)) + ", "

'strSQL = strSQL + "GearCatchSum.Gear1 =" + CStr(GearM(1)) + ", GearCatchSum.Prop1 =1, "

strSQL = strSQL + "GearCatchSum.Gear2 =" + CStr(GearM(2)) + ", GearCatchSum.Prop2 =" + CStr(ProM(2)) + ", "

strSQL = strSQL + "GearCatchSum.Gear3 =" + CStr(GearM(3)) + ", GearCatchSum.Prop3 =" + CStr(ProM(3)) + ", "

strSQL = strSQL + "GearCatchSum.Gear4 =" + CStr(GearM(4)) + ", GearCatchSum.Prop4 =" + CStr(ProM(4)) + ", "

strSQL = strSQL + "GearCatchSum.Gear5 =" + CStr(GearM(5)) + ", GearCatchSum.Prop5 =" + CStr(ProM(5)) + ", "

strSQL = strSQL + "GearCatchSum.Pedigree = '5' "

strSQL = strSQL + "WHERE GearCatchSum.Taxonkey=" + CStr(rst.Fields!TaxonKey.Value)

'Modify Summary Gear Records

rst2 = New ADODB.Recordset

rst2.Open(strSQL, curconn)

nextTax:

rst.MoveNext()

Loop

'==================================================================================================================================

P6A:

'=============================================== Tax AND FAO LEVEL 6A =========================================================

'Get the list of Taxonkey x FAO to use from GearCatchSum

Ret = ShrinkDatabase(frmAllo, "CATCH")

frm.Noticefrm(2, "Make CatchGear...")

strSQL = "SELECT GearCatchSum.Taxonkey, GearCatchSum.FAO, Sum(GearCatchSum.Catch) AS Catch "

strSQL = strSQL + "from GearCatchSum "

strSQL = strSQL + "where (GearCatchSum.Pedigree='1' or GearCatchSum.Pedigree='2A' or GearCatchSum.Pedigree='2B' or GearCatchSum.Pedigree='5') "

strSQL = strSQL + "GROUP BY GearCatchSum.Taxonkey, GearCatchSum.FAO "

strSQL = strSQL + "ORDER BY GearCatchSum.Taxonkey, GearCatchSum.FAO;"

'Get Records

rst = New ADODB.Recordset

rst.Open(strSQL, curconn)

'rst.MoveLast()

'NumRec = rst.RecordCount

'rst.MoveFirst()

Do Until rst.EOF

frm.Noticefrm(1, "6A: Taxon=" + CStr(rst.Fields!TaxonKey.Value) + " FAO=" + CStr(rst.Fields!FAO.Value))

'Get records for the Tax and FAO

strSQL = "SELECT GearTable.Taxonkey, GearTable.Gear1B, GearTable.Gear2B, GearTable.Gear3B, GearTable.Gear4B, GearTable.Gear5B, "

strSQL = strSQL + "GearTable.ProGear1, GearTable.ProGear2, GearTable.ProGear3, GearTable.ProGear4, GearTable.ProGear5, GearTable.Quality "

strSQL = strSQL + "FROM GearTable "

strSQL = strSQL + "WHERE GearTable.Taxonkey=" + CStr(rst.Fields!TaxonKey.Value) + " AND "

strSQL = strSQL + "GearTable.FAO=" + CStr(rst.Fields!FAO.Value)

'Get Gear Records

rst2 = New ADODB.Recordset

rst2.Open(strSQL, curconn)

'rst2.MoveLast()

'If rst2.RecordCount = 0 Then GoTo nextTaxFAO '*****************

Try

rst2.MoveFirst()

Catch

GoTo nextTaxFAO

End Try

ans = WeightRecords(rst2, MaxGears, GearM, ProM)

If ans = 0 Then GoTo nextTaxFAO '******************************

'Process results

strSQL = "UPDATE GearCatchSum SET "

strSQL = strSQL + "GearCatchSum.Gear1 =" + CStr(GearM(1)) + ", GearCatchSum.Prop1 =" + CStr(ProM(1)) + ", "

strSQL = strSQL + "GearCatchSum.Gear2 =" + CStr(GearM(2)) + ", GearCatchSum.Prop2 =" + CStr(ProM(2)) + ", "

strSQL = strSQL + "GearCatchSum.Gear3 =" + CStr(GearM(3)) + ", GearCatchSum.Prop3 =" + CStr(ProM(3)) + ", "

strSQL = strSQL + "GearCatchSum.Gear4 =" + CStr(GearM(4)) + ", GearCatchSum.Prop4 =" + CStr(ProM(4)) + ", "

strSQL = strSQL + "GearCatchSum.Gear5 =" + CStr(GearM(5)) + ", GearCatchSum.Prop5 =" + CStr(ProM(5)) + ", "

strSQL = strSQL + "GearCatchSum.Pedigree = '6A' "

strSQL = strSQL + "WHERE GearCatchSum.Taxonkey=" + CStr(rst.Fields!TaxonKey.Value) + " and "

strSQL = strSQL + "GearCatchSum.FAO=" + CStr(rst.Fields!FAO.Value)

'Modify Summary Gear Records

rst2 = New ADODB.Recordset

rst2.Open(strSQL, curconn)

nextTaxFAO:

rst.MoveNext()

Loop

'==================================================================================================================================

P7A:

'=============================================== Tax AND FAO AND COUNTRY LEVEL 7A =========================================================

'Get the list of Tax x FAO x COUNTRY to use from GearCatchSum

Ret = ShrinkDatabase(frmAllo, "CATCH")

frm.Noticefrm(2, "Make CatchGear...")

strSQL = "SELECT GearCatchSum.Taxonkey, GearCatchSum.FAO, GearCatchSum.CNumber, Sum(GearCatchSum.Catch) AS Catch "

strSQL = strSQL + "from GearCatchSum "

strSQL = strSQL + "GROUP BY GearCatchSum.Taxonkey, GearCatchSum.FAO, GearCatchSum.CNumber "

strSQL = strSQL + "ORDER BY GearCatchSum.Taxonkey, GearCatchSum.FAO, GearCatchSum.CNumber;"

'Get Records

rst = New ADODB.Recordset

rst.Open(strSQL, curconn)

'rst.MoveLast()

'NumRec = rst.RecordCount

'rst.MoveFirst()

Do Until rst.EOF

frm.Noticefrm(1, "7A: Taxon=" + CStr(rst.Fields!TaxonKey.Value) + " FAO=" + CStr(rst.Fields!FAO.Value) + " COUNTRY=" + CStr(rst.Fields!CNumber.Value))

'Get records for the Taxonkey

strSQL = "SELECT GearTable.Taxonkey, GearTable.Gear1B, GearTable.Gear2B, GearTable.Gear3B, GearTable.Gear4B, GearTable.Gear5B, "

strSQL = strSQL + "GearTable.ProGear1, GearTable.ProGear2, GearTable.ProGear3, GearTable.ProGear4, GearTable.ProGear5, GearTable.Quality "

strSQL = strSQL + "FROM GearTable "

strSQL = strSQL + "WHERE GearTable.Taxonkey=" + CStr(rst.Fields!TaxonKey.Value) + " AND "

strSQL = strSQL + "GearTable.FAO=" + CStr(rst.Fields!FAO.Value) + " and "

strSQL = strSQL + "GearTable.CNumber=" + CStr(rst.Fields!CNumber.Value)

'Get Gear Records

rst2 = New ADODB.Recordset

rst2.Open(strSQL, curconn)

'rst2.MoveLast()

'If rst2.RecordCount = 0 Then GoTo nextTaxFAOCount '*****************

Try

rst2.MoveFirst()

Catch

GoTo nextTaxFAOCount

End Try

ans = WeightRecords(rst2, MaxGears, GearM, ProM)

If ans = 0 Then GoTo nextTaxFAOCount '******************************

'Process results

strSQL = "UPDATE GearCatchSum SET "

strSQL = strSQL + "GearCatchSum.Gear1 =" + CStr(GearM(1)) + ", GearCatchSum.Prop1 =" + CStr(ProM(1)) + ", "

strSQL = strSQL + "GearCatchSum.Gear2 =" + CStr(GearM(2)) + ", GearCatchSum.Prop2 =" + CStr(ProM(2)) + ", "

strSQL = strSQL + "GearCatchSum.Gear3 =" + CStr(GearM(3)) + ", GearCatchSum.Prop3 =" + CStr(ProM(3)) + ", "

strSQL = strSQL + "GearCatchSum.Gear4 =" + CStr(GearM(4)) + ", GearCatchSum.Prop4 =" + CStr(ProM(4)) + ", "

strSQL = strSQL + "GearCatchSum.Gear5 =" + CStr(GearM(5)) + ", GearCatchSum.Prop5 =" + CStr(ProM(5)) + ", "

strSQL = strSQL + "GearCatchSum.Pedigree = '7A' "

strSQL = strSQL + "WHERE GearCatchSum.Taxonkey=" + CStr(rst.Fields!TaxonKey.Value) + " and "

strSQL = strSQL + "GearCatchSum.FAO=" + CStr(rst.Fields!FAO.Value) + " and "

strSQL = strSQL + "GearCatchSum.CNumber=" + CStr(rst.Fields!CNumber.Value)

'Modify Summary Gear Records

rst2 = New ADODB.Recordset

rst2.Open(strSQL, curconn)

nextTaxFAOCount:

rst.MoveNext()

Loop

'==================================================================================================================================

P8A:

'=============================================== Tax AND FAO AND COUNTRY AND YEAR LEVEL 8A =========================================================

'Get the list of Tax x FAO x COUNTRY x YEAR to use from GearCatchSum

Ret = ShrinkDatabase(frmAllo, "CATCH")

frm.Noticefrm(2, "Make CatchGear...")

strSQL = "SELECT GearCatchSum.Taxonkey, GearCatchSum.FAO, GearCatchSum.CNumber, GearCatchSum.IYear, Sum(GearCatchSum.Catch) AS Catch "

strSQL = strSQL + "from GearCatchSum "

strSQL = strSQL + "GROUP BY GearCatchSum.Taxonkey, GearCatchSum.FAO, GearCatchSum.CNumber, GearCatchSum.IYear "

strSQL = strSQL + "ORDER BY GearCatchSum.Taxonkey, GearCatchSum.FAO, GearCatchSum.CNumber, GearCatchSum.IYear;"

'Get Records

rst = New ADODB.Recordset

rst.Open(strSQL, curconn)

'rst.MoveLast()

'NumRec = rst.RecordCount

'rst.MoveFirst()

Do Until rst.EOF

frm.Noticefrm(1, "8A: Taxon=" + CStr(rst.Fields!TaxonKey.Value) + " FAO=" + CStr(rst.Fields!FAO.Value) + " COUNTRY=" + CStr(rst.Fields!CNumber.Value) + " YEAR=" + CStr(rst.Fields!IYear.Value))

'Get records for the Taxonkey

strSQL = "SELECT GearTable.Taxonkey, GearTable.Gear1B, GearTable.Gear2B, GearTable.Gear3B, GearTable.Gear4B, GearTable.Gear5B, "

strSQL = strSQL + "GearTable.ProGear1, GearTable.ProGear2, GearTable.ProGear3, GearTable.ProGear4, GearTable.ProGear5, GearTable.Quality "

strSQL = strSQL + "FROM GearTable "

strSQL = strSQL + "WHERE GearTable.Taxonkey=" + CStr(rst.Fields!TaxonKey.Value) + " AND "

strSQL = strSQL + "GearTable.FAO=" + CStr(rst.Fields!FAO.Value) + " and "

strSQL = strSQL + "GearTable.CNumber=" + CStr(rst.Fields!CNumber.Value) + " and "

strSQL = strSQL + "GearTable.FirstYear <=" + CStr(rst.Fields!IYear.Value) + " and "

strSQL = strSQL + "GearTable.LastYear >=" + CStr(rst.Fields!IYear.Value) + " and "

strSQL = strSQL + "(GearTable.FirstYear >" + CStr(CATCHparas.minYear) + " or GearTable.LastYear < 9999)"

'Get Gear Records

rst2 = New ADODB.Recordset

rst2.Open(strSQL, curconn)

'rst2.MoveLast()

'If rst2.RecordCount = 0 Then GoTo nextTaxFAOCountYr '*****************

Try

rst2.MoveFirst()

Catch

GoTo nextTaxFAOCountYr

End Try

ans = WeightRecords(rst2, MaxGears, GearM, ProM)

If ans = 0 Then GoTo nextTaxFAOCountYr '******************************

'Process results

strSQL = "UPDATE GearCatchSum SET "

strSQL = strSQL + "GearCatchSum.Gear1 =" + CStr(GearM(1)) + ", GearCatchSum.Prop1 =" + CStr(ProM(1)) + ", "

strSQL = strSQL + "GearCatchSum.Gear2 =" + CStr(GearM(2)) + ", GearCatchSum.Prop2 =" + CStr(ProM(2)) + ", "

strSQL = strSQL + "GearCatchSum.Gear3 =" + CStr(GearM(3)) + ", GearCatchSum.Prop3 =" + CStr(ProM(3)) + ", "

strSQL = strSQL + "GearCatchSum.Gear4 =" + CStr(GearM(4)) + ", GearCatchSum.Prop4 =" + CStr(ProM(4)) + ", "

strSQL = strSQL + "GearCatchSum.Gear5 =" + CStr(GearM(5)) + ", GearCatchSum.Prop5 =" + CStr(ProM(5)) + ", "

strSQL = strSQL + "GearCatchSum.Pedigree = '8A' "

strSQL = strSQL + "WHERE GearCatchSum.Taxonkey=" + CStr(rst.Fields!TaxonKey.Value) + " and "

strSQL = strSQL + "GearCatchSum.FAO=" + CStr(rst.Fields!FAO.Value) + " and "

strSQL = strSQL + "GearCatchSum.CNumber=" + CStr(rst.Fields!CNumber.Value) + " and "

strSQL = strSQL + "GearCatchSum.IYear=" + CStr(rst.Fields!IYear.Value)

'Modify Summary Gear Records

rst2 = New ADODB.Recordset

rst2.Open(strSQL, curconn)

nextTaxFAOCountYr:

rst.MoveNext()

Loop

'==================================================================================================================================

P6B:

'=============================================== Tax AND COUNTRY LEVEL 6B =========================================================

'Get the list of Tax x COUNTRY to use from GearCatchSum

Ret = ShrinkDatabase(frmAllo, "CATCH")

frm.Noticefrm(2, "Make CatchGear...")

strSQL = "SELECT GearCatchSum.Taxonkey, GearCatchSum.CNumber, Sum(GearCatchSum.Catch) AS Catch "

strSQL = strSQL + "from GearCatchSum "

strSQL = strSQL + "where GearCatchSum.Pedigree='5' or GearCatchSum.Pedigree='6A' "

strSQL = strSQL + "GROUP BY GearCatchSum.Taxonkey, GearCatchSum.CNumber "

strSQL = strSQL + "ORDER BY GearCatchSum.Taxonkey, GearCatchSum.CNumber;"

'Get Records

rst = New ADODB.Recordset

rst.Open(strSQL, curconn)

'rst.MoveLast()

'NumRec = rst.RecordCount

'rst.MoveFirst()

Do Until rst.EOF

frm.Noticefrm(1, "6B: Taxon=" + CStr(rst.Fields!TaxonKey.Value) + " COUNTRY=" + CStr(rst.Fields!CNumber.Value))

'Get records for the TG

strSQL = "SELECT GearTable.Taxonkey, GearTable.Gear1B, GearTable.Gear2B, GearTable.Gear3B, GearTable.Gear4B, GearTable.Gear5B, "

strSQL = strSQL + "GearTable.ProGear1, GearTable.ProGear2, GearTable.ProGear3, GearTable.ProGear4, GearTable.ProGear5, GearTable.Quality "

strSQL = strSQL + "FROM GearTable "

strSQL = strSQL + "WHERE GearTable.Taxonkey=" + CStr(rst.Fields!TaxonKey.Value) + " AND "

strSQL = strSQL + "GearTable.CNumber=" + CStr(rst.Fields!CNumber.Value) + " and "

strSQL = strSQL + "GearTable.Origcountcode<>0 "

'Get Gear Records

rst2 = New ADODB.Recordset

rst2.Open(strSQL, curconn)

'rst2.MoveLast()

'If rst2.RecordCount = 0 Then GoTo nextTaxCount '*****************

Try

rst2.MoveFirst()

Catch

GoTo nextTaxCount

End Try

ans = WeightRecords(rst2, MaxGears, GearM, ProM)

If ans = 0 Then GoTo nextTaxCount '******************************

'Process results

strSQL = "UPDATE GearCatchSum SET "

strSQL = strSQL + "GearCatchSum.Gear1 =" + CStr(GearM(1)) + ", GearCatchSum.Prop1 =" + CStr(ProM(1)) + ", "

strSQL = strSQL + "GearCatchSum.Gear2 =" + CStr(GearM(2)) + ", GearCatchSum.Prop2 =" + CStr(ProM(2)) + ", "

strSQL = strSQL + "GearCatchSum.Gear3 =" + CStr(GearM(3)) + ", GearCatchSum.Prop3 =" + CStr(ProM(3)) + ", "

strSQL = strSQL + "GearCatchSum.Gear4 =" + CStr(GearM(4)) + ", GearCatchSum.Prop4 =" + CStr(ProM(4)) + ", "

strSQL = strSQL + "GearCatchSum.Gear5 =" + CStr(GearM(5)) + ", GearCatchSum.Prop5 =" + CStr(ProM(5)) + ", "

strSQL = strSQL + "GearCatchSum.Pedigree = '6B' "

strSQL = strSQL + "WHERE GearCatchSum.Taxonkey=" + CStr(rst.Fields!TaxonKey.Value) + " and "

strSQL = strSQL + "GearCatchSum.CNumber=" + CStr(rst.Fields!CNumber.Value)

'Modify Summary Gear Records

rst2 = New ADODB.Recordset

rst2.Open(strSQL, curconn)

nextTaxCount:

rst.MoveNext()

Loop

'==================================================================================================================================

P7B:

'=============================================== Tax AND COUNTRY AND YEAR LEVEL 7B =========================================================

'Get the list of Tax x COUNTRY x YEAR to use from GearCatchSum

Ret = ShrinkDatabase(frmAllo, "CATCH")

frm.Noticefrm(2, "Make CatchGear...")

strSQL = "SELECT GearCatchSum.Taxonkey, GearCatchSum.CNumber, GearCatchSum.IYear, Sum(GearCatchSum.Catch) AS Catch "

strSQL = strSQL + "from GearCatchSum "

strSQL = strSQL + "where GearCatchSum.Pedigree='6B' "

strSQL = strSQL + "GROUP BY GearCatchSum.Taxonkey, GearCatchSum.CNumber, GearCatchSum.IYear "

strSQL = strSQL + "ORDER BY GearCatchSum.Taxonkey, GearCatchSum.CNumber, GearCatchSum.IYear;"

'Get Records

rst = New ADODB.Recordset

rst.Open(strSQL, curconn)

'rst.MoveLast()

'NumRec = rst.RecordCount

'rst.MoveFirst()

Do Until rst.EOF

frm.Noticefrm(1, "7B: Taxon=" + CStr(rst.Fields!TaxonKey.Value) + " COUNTRY=" + CStr(rst.Fields!CNumber.Value) + " YEAR=" + CStr(rst.Fields!IYear.Value))

'Get records for the TG

strSQL = "SELECT GearTable.Taxonkey, GearTable.Gear1B, GearTable.Gear2B, GearTable.Gear3B, GearTable.Gear4B, GearTable.Gear5B, "

strSQL = strSQL + "GearTable.ProGear1, GearTable.ProGear2, GearTable.ProGear3, GearTable.ProGear4, GearTable.ProGear5, GearTable.Quality "

strSQL = strSQL + "FROM GearTable "

strSQL = strSQL + "WHERE GearTable.Taxonkey=" + CStr(rst.Fields!TaxonKey.Value) + " AND "

strSQL = strSQL + "GearTable.CNumber=" + CStr(rst.Fields!CNumber.Value) + " and "

strSQL = strSQL + "GearTable.Origcountcode<>0 and "

strSQL = strSQL + "GearTable.FirstYear <=" + CStr(rst.Fields!IYear.Value) + " and "

strSQL = strSQL + "GearTable.LastYear >=" + CStr(rst.Fields!IYear.Value) + " and "

strSQL = strSQL + "(GearTable.FirstYear >" + CStr(CATCHparas.minYear) + " or GearTable.LastYear < 9999)"

'Get Gear Records

rst2 = New ADODB.Recordset

rst2.Open(strSQL, curconn)

'rst2.MoveLast()

'If rst2.RecordCount = 0 Then GoTo nextTaxCountYr '*****************

Try

rst2.MoveFirst()

Catch

GoTo nextTaxCountYr

End Try

ans = WeightRecords(rst2, MaxGears, GearM, ProM)

If ans = 0 Then GoTo nextTaxCountYr '******************************

'Process results

strSQL = "UPDATE GearCatchSum SET "

strSQL = strSQL + "GearCatchSum.Gear1 =" + CStr(GearM(1)) + ", GearCatchSum.Prop1 =" + CStr(ProM(1)) + ", "

strSQL = strSQL + "GearCatchSum.Gear2 =" + CStr(GearM(2)) + ", GearCatchSum.Prop2 =" + CStr(ProM(2)) + ", "

strSQL = strSQL + "GearCatchSum.Gear3 =" + CStr(GearM(3)) + ", GearCatchSum.Prop3 =" + CStr(ProM(3)) + ", "

strSQL = strSQL + "GearCatchSum.Gear4 =" + CStr(GearM(4)) + ", GearCatchSum.Prop4 =" + CStr(ProM(4)) + ", "

strSQL = strSQL + "GearCatchSum.Gear5 =" + CStr(GearM(5)) + ", GearCatchSum.Prop5 =" + CStr(ProM(5)) + ", "

strSQL = strSQL + "GearCatchSum.Pedigree = '7B' "

strSQL = strSQL + "WHERE GearCatchSum.Taxonkey=" + CStr(rst.Fields!TaxonKey.Value) + " and "

strSQL = strSQL + "GearCatchSum.CNumber=" + CStr(rst.Fields!CNumber.Value) + " and "

strSQL = strSQL + "GearCatchSum.Iyear=" + CStr(rst.Fields!IYear.Value)

'Modify Summary Gear Records

rst2 = New ADODB.Recordset

rst2.Open(strSQL, curconn)

nextTaxCountYr:

rst.MoveNext()

Loop

'==================================================================================================================================

P7C:

'=============================================== Tax AND FAO AND YEAR LEVEL 7C =========================================================

'Get the list of Tax x FAO x YEAR to use from GearCatchSum

Ret = ShrinkDatabase(frmAllo, "CATCH")

frm.Noticefrm(2, "Make CatchGear...")

strSQL = "SELECT GearCatchSum.Taxonkey, GearCatchSum.FAO, GearCatchSum.IYear, Sum(GearCatchSum.Catch) AS Catch "

strSQL = strSQL + "from GearCatchSum "

strSQL = strSQL + "where GearCatchSum.Pedigree='6A' "

strSQL = strSQL + "GROUP BY GearCatchSum.Taxonkey, GearCatchSum.FAO, GearCatchSum.IYear "

strSQL = strSQL + "ORDER BY GearCatchSum.Taxonkey, GearCatchSum.FAO, GearCatchSum.IYear;"

'Get Records

rst = New ADODB.Recordset

rst.Open(strSQL, curconn)

'rst.MoveLast()

'NumRec = rst.RecordCount

'rst.MoveFirst()

Do Until rst.EOF

frm.Noticefrm(1, "7C: Taxon=" + CStr(rst.Fields!TaxonKey.Value) + " FAO=" + CStr(rst.Fields!FAO.Value) + " YEAR=" + CStr(rst.Fields!IYear.Value))

'Get records for the TG

strSQL = "SELECT GearTable.Taxonkey, GearTable.Gear1B, GearTable.Gear2B, GearTable.Gear3B, GearTable.Gear4B, GearTable.Gear5B, "

strSQL = strSQL + "GearTable.ProGear1, GearTable.ProGear2, GearTable.ProGear3, GearTable.ProGear4, GearTable.ProGear5, GearTable.Quality "

strSQL = strSQL + "FROM GearTable "

strSQL = strSQL + "WHERE GearTable.Taxonkey=" + CStr(rst.Fields!TaxonKey.Value) + " AND "

strSQL = strSQL + "GearTable.FAO=" + CStr(rst.Fields!FAO.Value) + " and "

strSQL = strSQL + "GearTable.FirstYear <=" + CStr(rst.Fields!IYear.Value) + " and "

strSQL = strSQL + "GearTable.LastYear >=" + CStr(rst.Fields!IYear.Value) + " and "

strSQL = strSQL + "(GearTable.FirstYear >" + CStr(CATCHparas.minYear) + " or GearTable.LastYear < 9999)"

'Get Gear Records

rst2 = New ADODB.Recordset

rst2.Open(strSQL, curconn)

'rst2.MoveLast()

'If rst2.RecordCount = 0 Then GoTo nextTaxFAOYr '*****************

Try

rst2.MoveFirst()

Catch

GoTo nextTaxFAOYr

End Try

ans = WeightRecords(rst2, MaxGears, GearM, ProM)

If ans = 0 Then GoTo nextTaxFAOYr '******************************

'Process results

strSQL = "UPDATE GearCatchSum SET "

strSQL = strSQL + "GearCatchSum.Gear1 =" + CStr(GearM(1)) + ", GearCatchSum.Prop1 =" + CStr(ProM(1)) + ", "

strSQL = strSQL + "GearCatchSum.Gear2 =" + CStr(GearM(2)) + ", GearCatchSum.Prop2 =" + CStr(ProM(2)) + ", "

strSQL = strSQL + "GearCatchSum.Gear3 =" + CStr(GearM(3)) + ", GearCatchSum.Prop3 =" + CStr(ProM(3)) + ", "

strSQL = strSQL + "GearCatchSum.Gear4 =" + CStr(GearM(4)) + ", GearCatchSum.Prop4 =" + CStr(ProM(4)) + ", "

strSQL = strSQL + "GearCatchSum.Gear5 =" + CStr(GearM(5)) + ", GearCatchSum.Prop5 =" + CStr(ProM(5)) + ", "

strSQL = strSQL + "GearCatchSum.Pedigree = '2A' "

strSQL = strSQL + "WHERE GearCatchSum.Taxonkey=" + CStr(rst.Fields!TaxonKey.Value) + " and "

strSQL = strSQL + "GearCatchSum.FAO=" + CStr(rst.Fields!FAO.Value) + " and "

strSQL = strSQL + "GearCatchSum.Iyear=" + CStr(rst.Fields!IYear.Value)

'Modify Summary Gear Records

rst2 = New ADODB.Recordset

rst2.Open(strSQL, curconn)

nextTaxFAOYr:

rst.MoveNext()

Loop

'==================================================================================================================================

End If 'GearCatchSum Filled

Ret = ShrinkDatabase(frmAllo, "CATCH")

frm.Noticefrm(2, "Make CatchGear...")

' FLATTEN GearCatchSum to produce GeartoSQL ==========================================================================================

If flgMakeGeartoSQL = True Then

'drop GeartoSQL if it exists before remaking

Try

strSQL = "drop Table GeartoSQL"

rst = New ADODB.Recordset

rst.Open(strSQL, curconn)

Catch ex As System.Exception

End Try

' Make the GeartoSQL table

strSQL = "CREATE TABLE [dbo].[GeartoSQL] "

strSQL = strSQL + "( IYear INT Not Null, "

strSQL = strSQL + " CNumber INT Not Null, "

strSQL = strSQL + " Taxonkey INT Not Null, "

strSQL = strSQL + " FAO INT Not Null, "

strSQL = strSQL + " IGear INT Not Null, "

strSQL = strSQL + " Prop FLOAT Not Null, "

strSQL = strSQL + " [Catch] Float Not Null, "

strSQL = strSQL + " Pedigree nvarchar(50) null "

strSQL = strSQL + " ) "

rst = New ADODB.Recordset

rst.Open(strSQL, curconn)

'Put data into GeartoSQL Table

strSQL = "INSERT INTO GeartoSQL (IYear, CNumber, Taxonkey, FAO, IGear, Prop, [Catch], Pedigree) "

strSQL = strSQL + "SELECT IYear, CNumber, Taxonkey, FAO, Gear1 as IGear, Prop1 as Prop, Prop1 * [Catch] AS [Catch], Pedigree "

strSQL = strSQL + " FROM GearCatchSum "

strSQL = strSQL + " WHERE Gear1>0 and Prop1>0 and [Catch]>0 "

strSQL = strSQL + " ORDER BY IYear, CNumber, Taxonkey "

'Write Records

rst = New ADODB.Recordset

rst.Open(strSQL, curconn)

strSQL = "INSERT INTO GeartoSQL (IYear, CNumber, Taxonkey, FAO, IGear, Prop, [Catch], Pedigree) "

strSQL = strSQL + "SELECT IYear, CNumber, Taxonkey, FAO, Gear2 as IGear, Prop2 as Prop, Prop2 * [Catch] AS [Catch], Pedigree "

strSQL = strSQL + "FROM GearCatchSum "

strSQL = strSQL + " WHERE Gear2>0 and Prop2>0 and [Catch]>0 "

strSQL = strSQL + " ORDER BY IYear, CNumber, Taxonkey "

'Write Records

rst = New ADODB.Recordset

rst.Open(strSQL, curconn)

strSQL = "INSERT INTO GeartoSQL (IYear, CNumber, Taxonkey, FAO, IGear, Prop, [Catch], Pedigree) "

strSQL = strSQL + "SELECT IYear, CNumber, Taxonkey, FAO, Gear3 as IGear, Prop3 as Prop, Prop3 * [Catch] AS [Catch], Pedigree "

strSQL = strSQL + "FROM GearCatchSum "

strSQL = strSQL + " WHERE Gear3>0 and Prop3>0 and [Catch]>0 "

strSQL = strSQL + " ORDER BY IYear, CNumber, Taxonkey "

'Write Records

rst = New ADODB.Recordset

rst.Open(strSQL, curconn)

strSQL = "INSERT INTO GeartoSQL (IYear, CNumber, Taxonkey, FAO, IGear, Prop, [Catch], Pedigree) "

strSQL = strSQL + "SELECT IYear, CNumber, Taxonkey, FAO, Gear4 as IGear, Prop4 as Prop, Prop4 * [Catch] AS [Catch], Pedigree "

strSQL = strSQL + "FROM GearCatchSum "

strSQL = strSQL + " WHERE Gear4>0 and Prop4>0 and [Catch]>0 "

strSQL = strSQL + " ORDER BY IYear, CNumber, Taxonkey "

'Write Records

rst = New ADODB.Recordset

rst.Open(strSQL, curconn)

strSQL = "INSERT INTO GeartoSQL (IYear, CNumber, Taxonkey, FAO, IGear, Prop, [Catch], Pedigree) "

strSQL = strSQL + "SELECT IYear, CNumber, Taxonkey, FAO, Gear5 as IGear, Prop5 as Prop, Prop5 * [Catch] AS [Catch], Pedigree "

strSQL = strSQL + "FROM GearCatchSum "

strSQL = strSQL + " WHERE Gear5>0 and Prop5>0 and [Catch]>0 "

strSQL = strSQL + " ORDER BY IYear, CNumber, Taxonkey "

'Write Records

rst = New ADODB.Recordset

rst.Open(strSQL, curconn)

'build primary key index

strSQL = "ALTER TABLE GeartoSQL ADD CONSTRAINT PK_GeartoSQL PRIMARY KEY CLUSTERED ( IYear, CNumber, Taxonkey, FAO, IGear)"

rst = New ADODB.Recordset

rst.Open(strSQL, curconn)

End If 'GeartoSQL Created

Ret = ShrinkDatabase(frmAllo, "CATCH")

frm.Noticefrm(2, "Make CatchGear...")

TableMakeCatchGear:

'drop CatchGear (if exists)

If flgMakeCatchGear = True Then

Try

strSQL = " Drop Table CatchGear"

rst = New ADODB.Recordset

rst.Open(strSQL, curconn)

Catch ex As Exception

End Try

Ret = ShrinkDatabase(frmAllo, "CATCH")

Delay(300) 'ALLOW ANOTHER 5 MIN TO MAKE SURE LOG SHRUNK

'This table is by cell so it is a huge breakdown of catch by cell by gear as well as country, year and taxonkey!

' Make the CatchSum table

strSQL = "CREATE TABLE [dbo].[CatchGear] "

strSQL = strSQL + "( IYear INT Not Null, "

strSQL = strSQL + " CNumber INT Not Null, "

strSQL = strSQL + " Taxonkey INT Not Null, "

strSQL = strSQL + " EEZ INT Not Null, "

strSQL = strSQL + " Cell INT Not Null, "

strSQL = strSQL + " Gear INT Not Null, "

strSQL = strSQL + " Catchrate FLOAT Not Null "

strSQL = strSQL + " ) "

rst = New ADODB.Recordset

rst.Open(strSQL, curconn)

For IYear = StartYear To EndYear

strSQL = "INSERT INTO CatchGear (IYear, CNumber, Taxonkey, EEZ, Cell, Gear, Catchrate) "

strSQL = strSQL + "SELECT [Catch].IYear AS IYear, [Catch].CNumber AS CNumber, [Catch].Taxonkey AS Taxonkey, [Catch].EEZ AS EEZ, [Catch].Cell AS Cell, GeartoSQL.IGear AS Gear, "

strSQL = strSQL + "[Catch].CatchRate * GeartoSQL.Prop AS Catchrate "

strSQL = strSQL + "FROM GeartoSQL INNER JOIN "

strSQL = strSQL + "[Catch] ON GeartoSQL.CNumber = [Catch].CNumber AND GeartoSQL.Taxonkey = [Catch].Taxonkey AND GeartoSQL.IYear = [Catch].IYear INNER JOIN "

strSQL = strSQL + "World ON [Catch].Cell = World.Seq AND Geartosql.FAO = World.FAO "

strSQL = strSQL + "WHERE (Catch.IYear =" + CStr(IYear) + ") "

strSQL = strSQL + "ORDER BY [Catch].IYear, [Catch].CNumber, [Catch].Taxonkey, [Catch].EEZ, [Catch].Cell, GeartoSQL.IGear"

rst = New ADODB.Recordset

rst.Open(strSQL, curconn)

Ret = ShrinkDatabase(frmAllo, "CATCH")

Next IYear

Ret = ShrinkDatabase(frmAllo, "CATCH")

frm.Noticefrm(2, "Indexing CatchGear...")

IndexCatchGear:

'SQL to add index but will on work if Gear is “nullable”

'sometimes not unique so will not work

'strSQL = "ALTER TABLE CatchGear WITH NOCHECK ADD CONSTRAINT [PK_CatchG] PRIMARY KEY CLUSTERED (IYear, CNumber, Taxonkey, EEZ, Gear, Cell)"

'rst = New ADODB.Recordset

'rst.Open(strSQL, curconn)

'--create cell index as often joining with world table

strSQL = "CREATE CLUSTERED INDEX idx_cell ON CatchGear (IYear, CNumber, Taxonkey, EEZ, Gear, Cell)"

rst = New ADODB.Recordset

rst.Open(strSQL, curconn)

End If

Ret = ShrinkDatabase(frmAllo, "CATCH")

frm.Noticefrm(2, "Make CatchGear completed")

frm.Noticefrm(1, "Finished Gear...")

endgear:

Windows.Forms.Cursor.Current = System.Windows.Forms.Cursors.Default

rst = Nothing

curconn = Nothing

End Function

**7. Estimating Unreported Catch**

Function ProcessCatchExport(ByVal frm As Form1) As Boolean

Dim InputFile As String

Dim FolderName As String

Dim CellDataFile As String

Dim Count As Long = 0

Dim RecordCount(LastYear - FirstYear) As Long

Dim LSTonnes(LastYear - FirstYear) As Double

Dim SSFTonnes(LastYear - FirstYear) As Double

Dim IUUTonnes(LastYear - FirstYear) As Double

Dim DiscardTonnes(LastYear - FirstYear) As Double

Dim SSFCell(MaxCell) As Boolean

Dim SSFCellCR(MaxCell, (LastYear - FirstYear) + 1) As Double

ReDim YearName(13) 'one for each 5-year block starting with Data5054... ending with Data1519

ReDim FirstYr(13)

ReDim LastYr(13)

Dim CountryNames(999) As String

Dim CountryHDI(999) As Single

Dim CountryReportRate(999) As Single

Dim CountrySSFCR(999) As Single

Dim GearFile As String

Dim GearNames(1650) As String

Dim TaxonFile As String

ReDim IUUTime(LastYear - FirstYear)

'THIS IS USED ONLY TO CREATE THE FILES RECORDING TOTAL OF CATCH IN SSF CANDIDATE CELLS FOR LATER USE

Dim CreatingSSFTotals As Boolean = False

'%%%%%%%%%%%%%%%%%%%%%%%%%%%%%%%%%%%%%%%%%%%%%%%%%%%%%%%%%%

FolderName = "E:/RWatson/CatchforWeb/"

InputFile = FolderName + "CatchGear.csv"

CellDataFile = FolderName + "CellData.csv"

GrandTotalFile = FolderName + "GrandTotals.csv"

Dim swGrandTotalFile = New IO.StreamWriter(GrandTotalFile)

swGrandTotalFile.WriteLine("Year,Records,LS,SSF,IUU,Discards")

'Sept 2016

'Takes an csv output of CatchGear and processes it for publication

'data has to be in the 5-year blocks required for processing and output

'2nd version

Windows.Forms.Cursor.Current = Windows.Forms.Cursors.WaitCursor

frm.ToolStripStatusLabel1.Text = "Preparing Files..."

Application.DoEvents()

'FILE NAMES AND RANGES OF YEARS

YearName(0) = "5054"

FirstYr(0) = 1950

LastYr(0) = 1954

YearName(1) = "5559"

FirstYr(1) = 1955

LastYr(1) = 1959

YearName(2) = "6064"

FirstYr(2) = 1960

LastYr(2) = 1964

YearName(3) = "6569"

FirstYr(3) = 1965

LastYr(3) = 1969

YearName(4) = "7074"

FirstYr(4) = 1970

LastYr(4) = 1974

YearName(5) = "7579"

FirstYr(5) = 1975

LastYr(5) = 1979

YearName(6) = "8084"

FirstYr(6) = 1980

LastYr(6) = 1984

YearName(7) = "8589"

FirstYr(7) = 1985

LastYr(7) = 1989

YearName(8) = "9094"

FirstYr(8) = 1990

LastYr(8) = 1994

YearName(9) = "9599"

FirstYr(9) = 1995

LastYr(9) = 1999

YearName(10) = "0004"

FirstYr(10) = 2000

LastYr(10) = 2004

YearName(11) = "0509"

FirstYr(11) = 2005

LastYr(11) = 2009

YearName(12) = "1014"

FirstYr(12) = 2010

LastYr(12) = 2014

YearName(13) = "1519"

FirstYr(13) = 2015

LastYr(13) = 2019

'============================

'IUU Factors =============================================================

'Agnew DJ, Pearce J, Pramod G, Peatman T, Watson R, Beddington JR, et al. Estimating the worldwide extent of illegal fishing. PLoS One. 2009;4(2):e4570. doi: 10.1371/journal.pone.0004570.

'Time Factor

For Year = 0 To LastYear - FirstYear

If Year < 1995 Then

IUUTime(Year) = 0.73

ElseIf Year > 1994 And Year < 2000 Then

IUUTime(Year) = 0.7

Else

IUUTime(Year) = 0.64

End If

Next Year

'Area Factor - one for every FAO major statistical area

IUUArea(18) = 0.1 'Arctic

IUUArea(21) = 2.87 'Atlantic, Northwest

IUUArea(27) = 1.38 'Atlantic, Northeast

IUUArea(31) = 0.81 'Atlantic, Western Central

IUUArea(34) = 14.38 'Atlantic, Eastern Central

IUUArea(37) = 7.27 'Mediterrean and Black Sea

IUUArea(41) = 6.99 'Atlantic, Southwest

IUUArea(47) = 7.27 'Atlantic, Southeast

IUUArea(48) = 0.1 'Atlantic, Antartic

IUUArea(51) = 8.75 'Indian Ocean, Western

IUUArea(57) = 11.65 'Indian Ocean, Eastern

IUUArea(58) = 0.1 'Indian Ocean, Antarctic

IUUArea(61) = 5.62 'Pacific, Northwest

IUUArea(67) = 2.72 'Pacific, Northeast

IUUArea(71) = 16.68 'Pacific, Western Central

IUUArea(77) = 4.07 'Pacific, Eastern Central

IUUArea(81) = 0.41 'Pacific, Southwest

IUUArea(87) = 7.31 'Pacific, Southeast

IUUArea(88) = 0.1 'Pacific, Antarctic

IUUArea(98) = 0.1 'Antarctic nei

IUUArea(99) = 2 'Marine areas outside the Antarctic

'Taxa Factor ' based on ISSCAAP

IUUTaxa(23) = 1.62 'Salmonids

IUUTaxa(24) = 0.29 'Shads

IUUTaxa(25) = 1.62 'Diadromous Fishes

IUUTaxa(31) = 0.09 'Flounders

IUUTaxa(32) = 0.24 'Gadiformes

IUUTaxa(33) = 0.06 'Periformes

IUUTaxa(34) = 0.06 'Mixed Order

IUUTaxa(35) = 0.21 'Herrings, sardines, anchovies

IUUTaxa(36) = 0.05 'Tunas, bonitos, billfishes

IUUTaxa(37) = 0.17 'Pelagics surface mostly

IUUTaxa(38) = 0.06 'Marine fishes nei

IUUTaxa(39) = 0.06 'Finfishes

IUUTaxa(42) = 0.29 'Crabs

IUUTaxa(43) = 0.58 'Lobsters, spiny-rock lobsters

IUUTaxa(44) = 0.29 'Craylets, squat lobsters

IUUTaxa(45) = 0.41 'Shrimps, prawns

IUUTaxa(46) = 2.07 'Krill

IUUTaxa(47) = 2.07 'Marine crustaceans

IUUTaxa(52) = 0.52 'Gastropods

IUUTaxa(53) = 4.7 'Oysters

IUUTaxa(54) = 4.7 'Mussels

IUUTaxa(55) = 0.98 'Scallops

IUUTaxa(56) = 0.52 'Clams, cockles, arkshells

IUUTaxa(57) = 0.25 'Cephalopods

IUUTaxa(58) = 0.52 'Marine molluscs

IUUTaxa(74) = 0.25 'Sea Squirt

IUUTaxa(75) = 2.07 'Horseshoe crab

IUUTaxa(76) = 0.98 'Starfishes & Urchins

IUUTaxa(95) = 0.52 'Ex Mollusca

'=========================================================================================================

'DISCARD RATES % ======================================================================

Discards(100) = 0.1 'without gear 0.1

Discards(110) = 0.1 'grasping with hand 0.1

Discards(120) = 0.1 'by diving 0.1

Discards(213) = 0.1 'raking devices 0.1

Discards(214) = 0.1 'tongs(0.1)

Discards(221) = 0.1 'spears and lances 0.1

Discards(225) = 0.1 'harpoons(0.1)

Discards(312) = 60 'explosives(60)

Discards(400) = 1.4 'Lines(1.4)

Discards(420) = 2 'with gorges or hooks 2

Discards(421) = 2 'handlines(2)

Discards(422) = 1.4 'set lines 1.4

Discards(423) = 1.4 'drift lines 1.4

Discards(424) = 1.4 'troll lines 1.4

Discards(432) = 0.1 'pilks or jigs 0.1

Discards(433) = 0.1 'squid hooks 0.1

Discards(440) = 0.4 'pole line tuna 0.4

Discards(450) = 28.5 'longline tuna 28.5

Discards(500) = 23.2 'traps(23.2)

Discards(540) = 23.2 'tubular traps 23.2

Discards(551) = 23.2 'pots(23.2)

Discards(552) = 23.2 'conical and drum-like traps 23.2

Discards(553) = 23.2 'box-like traps 23.2

Discards(560) = 23.2 'trapping gear 23.2

Discards(563) = 23.2 'pound nets 23.2

Discards(700) = 23.2 'bagnets(23.2)

Discards(830) = 28.3 'dredges(28.3)

Discards(831) = 28.3 'hand dredges 28.3

Discards(840) = 62.3 'bottom trawls 62.3

Discards(850) = 3.4 'mid-water trawls 3.4

Discards(900) = 1.2 'seine nets 1.2

Discards(920) = 0.1 'genuine seine net 0.1

Discards(921) = 0.1 'beach seines 0.1

Discards(922) = 1.2 'boat seines 1.2

Discards(1000) = 0.1 'surrounding nets 0.1

Discards(1010) = 0.1 'lampara-like nets 0.1

Discards(1020) = 1.2 'purse seines 1.2

Discards(1030) = 0.1 'ring nets 0.1

Discards(1050) = 5.1 'purse seine tuna 5.1

Discards(1100) = 0.1 'drive-in nets 0.1

Discards(1200) = 0.1 'liftnets or dipnets 0.1

Discards(1210) = 0.1 'hand liftnets 0.1

Discards(1320) = 0.1 'cast nets, with or without pockets 0.1

Discards(1400) = 0.5 'gillnets(0.5)

Discards(1410) = 0.5 'set gillnets 0.5

Discards(1420) = 0.5 'driftnets(0.5)

Discards(1500) = 0.5 'tangle nets 0.5

Discards(1540) = 0.5 'trammel nets 0.5

'===============================================================================

'READ CELL Data =============================================================

Dim srCell As New IO.StreamReader(CellDataFile)

InStr = srCell.ReadLine ' read labels

'Seq Lon Lat TArea Area Bathy_Min FAO LME Distance

Do While srCell.Peek <> -1

InStr = srCell.ReadLine

StrBits = InStr.Split(",")

Seq = CLng(StrBits(0))

CellData(Seq).Lon = CSng(StrBits(1))

CellData(Seq).Lat = CSng(StrBits(2))

CellData(Seq).Row = CSng(StrBits(3))

CellData(Seq).Col = CSng(StrBits(4))

CellData(Seq).TArea = CSng(StrBits(5))

CellData(Seq).Area = CSng(StrBits(6))

CellData(Seq).BathyMin = CLng(StrBits(7))

CellData(Seq).FAO = CLng(StrBits(8))

CellData(Seq).LME = CLng(StrBits(9))

CellData(Seq).Distance = CSng(StrBits(10))

'decide on SSF based on rules in Chuenpagdee, R., Liguori, L., Palomares, M.L.D., Pauly, D. (2006) Bottom up, global estimates of small-scale marine fisheries catches. Fisheries Centre Research Reports, 105pp.

'areas of the Antarctic have no SSF so cells set to False

'areas too deep - with a min Depth > 200 m area set to False

'areas to far from shore - > 50 km are set to False

'all catch provided by SAUP for the Arctic is deemed to be SSF

If CellData(Seq).Distance <= 50 And CellData(Seq).BathyMin <= 200 And (CellData(Seq).FAO <> 58 And CellData(Seq).FAO <> 88 And CellData(Seq).FAO <> 48) Then

CellData(Seq).SSF = True

Else

CellData(Seq).SSF = False

End If

Loop '==============================================================================

'Read Country Data ================================================================

CountryFile = FolderName + "CountrySSF.csv"

Dim srCountry As New IO.StreamReader(CountryFile)

InStr = srCountry.ReadLine ' read labels

'CNumber CountryName CorruptionIndexRecent CorruptionPercentileRecent HDIavg200611 GINICo EEZ SSFReportRate

Do While srCountry.Peek <> -1

InStr = srCountry.ReadLine

StrBits = InStr.Split(",")

CNumber = CLng(StrBits(0))

CountryNames(CNumber) = StrBits(1)

CountryHDI(CNumber) = CSng(StrBits(4))

CountryReportRate(CNumber) = CSng(StrBits(7))

'Chuenpagdee, R., Liguori, L., Palomares, M.L.D., Pauly, D. (2006) Bottom up, global estimates of small-scale marine fisheries catches. Fisheries Centre Research Reports, 105pp.

'Development Index’ (HDI). This index, developed by the United Nations Development Program (UNDP, 2000), defines countries

'as high HDI (≥ 0.8), medium (0.5 ≤ HDI < 0.8), and low HDI (< 0.5). HDI measures a country’s

If CountryHDI(CNumber) >= 0.8 Then 'High

CountrySSFCR(CNumber) = 0.77 't sq km

ElseIf CountryHDI(CNumber) >= 0.5 And CountryHDI(CNumber) < 0.8 Then

CountrySSFCR(CNumber) = 1.26 'Medium

Else

CountrySSFCR(CNumber) = 2.93 'Low

End If

Loop

'READ GEAR DATA ====================================================

'ICode, Gear, SuperCode, SuperName

GearFile = FolderName + "GearData.csv"

Dim srGear As New IO.StreamReader(GearFile)

InStr = srGear.ReadLine ' read labels

Do While srGear.Peek <> -1

InStr = srGear.ReadLine

StrBits = InStr.Split(",")

Gear = CLng(StrBits(0))

GearNames(Gear) = StrBits(1).Trim

Loop

'====================================================================

'TAXONOMIC DATA =====================================================

'TaxonKey,TaxonName,CommonName,TargetGrpNum,SuperTarget

TaxonFile = FolderName + "TaxonData.csv"

Dim srTaxon As New IO.StreamReader(TaxonFile)

InStr = srTaxon.ReadLine ' read labels

Do While srTaxon.Peek <> -1

InStr = srTaxon.ReadLine

StrBits = InStr.Split(",")

Taxonkey = CLng(StrBits(0))

If TaxaLookup(Taxonkey) = 0 Then

'add it

TaxaUsed = TaxaUsed + 1

TaxaLookup(Taxonkey) = TaxaUsed

End If

TaxonNames(TaxaUsed) = StrBits(1).Trim

CommonNames(TaxaUsed) = StrBits(2).Trim

ISSCAAPS(TaxaUsed) = CLng(StrBits(3))

Loop '====================================================================

For J = 0 To 12 '==================================================================================

CatchInFile = FolderName + "CatchExport" + YearName(J) + ".csv"

CatchOutFile = FolderName + "CatchPublic" + YearName(J) + ".csv"

CheckFile = FolderName + "CheckTotals" + YearName(J) + ".csv"

Dim swCheckFile = New IO.StreamWriter(CheckFile)

frm.ToolStripStatusLabel1.Text = "Years " + YearName(J)

Application.DoEvents()

'TOTAL CR IN VALID SSF CELLS READ IN 5-YEAR BLOCKS ================================

'Current total catch rates in SSF cells annually - MADE NOW OR PREPARED EARLIER

SSFCellCRFile = FolderName + "SSFCellCR" + YearName(J) + ".csv"

If CreatingSSFTotals Then

'SSF FILES MADE NOW

Else

'read existing totals PREPARED EARLIER

Dim srCR As New IO.StreamReader(SSFCellCRFile)

InStr = srCR.ReadLine ' read labels

'IYear Cell CR

Do While srCR.Peek <> -1

InStr = srCR.ReadLine

StrBits = InStr.Split(",")

Year = CLng(StrBits(0))

Seq = CLng(StrBits(1))

SSFCellCR(Seq, Year - FirstYear) = CDbl(StrBits(2))

Loop

'===============================================================================

End If

Dim swCatchOut As New IO.StreamWriter(CatchOutFile)

swCatchOut.WriteLine("Year, Seq, Lat, Lon, OceanArea, CNumber, CountryName, Taxonkey, TaxonName, CommonName, Gear, GearName, LSF_CR, SSF_CR, IUU_CR, Discards_CR")

Count = 0

Dim srInput As New IO.StreamReader(CatchInFile)

InStr = srInput.ReadLine ' labels

Do While srInput.Peek <> -1

InStr = srInput.ReadLine

StrBits = InStr.Split(",")

'IYear CNumber Taxonkey Cell Gear Catchrate

'1950 276 100039 24942 840 1.3364797135796E-05

Count = Count + 1

If Count < 100000 Then

If Count Mod 1000 = 0 Then

If CreatingSSFTotals Then

frm.ToolStripStatusLabel1.Text = "SSF Only Years " + YearName(J) + ": " + Count.ToString

Else

frm.ToolStripStatusLabel1.Text = "Years " + YearName(J) + ": " + Count.ToString

End If

Application.DoEvents()

Else

GoTo NoDisplay

End If

Else

If Count Mod 100000 = 0 Then

If CreatingSSFTotals Then

frm.ToolStripStatusLabel1.Text = "SSF Only Years " + YearName(J) + ": " + (Count / 1000000).ToString("F1") + "M"

Else

frm.ToolStripStatusLabel1.Text = "Years " + YearName(J) + ": " + (Count / 1000000).ToString("F1") + "M"

End If

Application.DoEvents()

End If

End If

NoDisplay:

Year = CLng(StrBits(0))

CNumber = CLng(StrBits(1))

Taxonkey = CLng(StrBits(2))

Seq = CLng(StrBits(3))

Gear = CLng(StrBits(4))

CR = CDbl(StrBits(5))

IndexYear = Year - FirstYear

FAO = CellData(Seq).FAO

OceanArea = CellData(Seq).Area

Lat = CellData(Seq).Lat

Lon = CellData(Seq).Lon

CountryName = CountryNames(CNumber)

GearName = GearNames(Gear)

TaxonName = TaxonNames(TaxaLookup(Taxonkey))

CommonName = CommonNames(TaxaLookup(Taxonkey))

ISSCAAP = ISSCAAPS(TaxaLookup(Taxonkey))

'ALL FISHING IN AREA 18 PROVIDED BY SAUP IS NOT LARGE SCALE THEREFORE THOSE GEAR ASSOCIATIONS

'MUST BE ASSOCIATED IF LARGE SCALE GEAR

'CHANGE BOTTOM TRAWL AND MID-WATER TRAWLS TO GILLNET (MOSTLY COREGONIDS CAUGHT WITH THE TRAWL ASSOCIATION)

If FAO = 18 Then

If Gear = 840 Or Gear = 850 Then

'ALTER GEAR TO GILLNET

Gear = 1400

End If

End If

'Assignment of SSF CR ===================================================================================

If CellData(Seq).FAO = 18 Then

'SAUP data from Arctic and all reported CR is SSF

LSCR = 0

SSFCR = CR

'IF MAKING SSF FILES

If CreatingSSFTotals Then

SSFCellCR(Seq, IndexYear) = SSFCellCR(Seq, IndexYear) + CR

End If

Else

'requires a cell with CellData(Seq).SSF = TRUE and large scale gear (ie not tuna purse seine or similar)

'note Antarctic FAO areas (48 and 48 and 88) already marked as False for SSF

If CellData(Seq).SSF = False Or Gear = 450 Or Gear = 1050 Then

SSFCR = 0

LSCR = CR 'all catch deemed to be large scale and none as small scale

Else

'the problem is that there is a target SSF_CR based on the country HDI but the number of valid records for CR is not known

'therefore the sum of CR for each valid cell must be known and the SSF_CR for each record here prorated as part of that toward the target CR

'further I found that the global estimates were exceeded if you used the published values for SSF_CR from the paper so these have to be

'prorated on the assumption that this is an area adjustment - ie only 45% of the inshore area is fished at the SSF_CR estimated (the rest not fished likely)

'the other assumption is that none, some or all of the SSF catch might be already reported in the CR (via FAO etc)

'Arctic is a special case were all catch is SSF and CR = 0

'Antarctic has no SSF so all CR is retained

'others depend on the Corruption Percentile Recent already calculated in the Country data file

'cr due to HDI category * proportion of cr of this records in this cell/year * prop of area represented by cr in paper

If CellData(Seq).SSF Then 'for valid ssf cells

If CreatingSSFTotals Then

'IF MAKING SSF FILES

SSFCellCR(Seq, IndexYear) = SSFCellCR(Seq, IndexYear) + CR

Else

If SSFCellCR(Seq, IndexYear) < CR - 0.0001 Then Stop

SSFCR = CountrySSFCR(CNumber) * (CR / SSFCellCR(Seq, IndexYear)) * 0.45

Reported = SSFCR * CountryReportRate(CNumber) 'amount of SSF already reported in the CR

LSCR = CR - Reported 'remove portion assumed reported in CR based on Corruption of country assumed related to likelihood of reporting

If LSCR < 0 Then LSCR = 0 ' in some areas all catch will be SSF

End If

End If

End If

End If

If CreatingSSFTotals Then GoTo Skipwriting

'Assignment of IUU =======================================================================================

'Agnew DJ, Pearce J, Pramod G, Peatman T, Watson R, Beddington JR, et al. Estimating the worldwide extent of illegal fishing. PLoS One. 2009;4(2):e4570. doi: 10.1371/journal.pone.0004570.

'proportion assumed to be on all fishing

IUUCR = (LSCR + SSFCR) * ((20 * IUUTime(IndexYear) * IUUArea(FAO) * IUUTaxa(ISSCAAP)) / 100)

'Assignment of Discards ==================================================================================

'Kelleher K. Discards in the world’s marine fisheries. An update. Rome: FAO, 2005 Contract No.: 470.

'proportion assumed only occuring in LS fisheries

DiscardCR = LSCR * (Discards(Gear) / 100)

'Totals for the Year

RecordCount(IndexYear) = RecordCount(IndexYear) + 1

LSTonnes(IndexYear) = LSTonnes(IndexYear) + (LSCR * OceanArea)

SSFTonnes(IndexYear) = SSFTonnes(IndexYear) + (SSFCR * OceanArea)

IUUTonnes(IndexYear) = IUUTonnes(IndexYear) + (IUUCR * OceanArea)

DiscardTonnes(IndexYear) = DiscardTonnes(IndexYear) + (DiscardCR * OceanArea)

'print out result ==========================================================================

'"Year, Seq, Lat, Lon, OceanArea, CNumber, CountryName, Taxonkey, TaxonName, CommonName, Gear, GearName,

' LSF_CR, SSF_CR, IUU_CR, Discards_CR"

LineOut = CStr(Year) + "," + CStr(Seq) + "," + CStr(Lat) + "," + CStr(Lon) + "," + OceanArea.ToString("F2") + ","

LineOut = LineOut + CStr(CNumber) + "," + CountryName + "," + CStr(Taxonkey) + ","

LineOut = LineOut + TaxonName + "," + CommonName + "," + CStr(Gear) + "," + GearName + ","

If LSCR = 0 Then

LineOut = LineOut + "0,"

Else

LineOut = LineOut + LSCR.ToString("F8") + ","

End If

If SSFCR = 0 Then

LineOut = LineOut + "0,"

Else

LineOut = LineOut + SSFCR.ToString("F8") + ","

End If

If IUUCR = 0 Then

LineOut = LineOut + "0,"

Else

LineOut = LineOut + IUUCR.ToString("F8") + ","

End If

If DiscardCR = 0 Then

LineOut = LineOut + "0"

Else

LineOut = LineOut + DiscardCR.ToString("F8")

End If

swCatchOut.WriteLine(LineOut)

swCatchOut.Flush()

Skipwriting:

Loop

If CreatingSSFTotals Then GoTo SkiptoJ

swCheckFile.WriteLine("Year,Records,LS,SSF,IUU,Discards")

For I = FirstYr(J) To LastYr(J)

IndexYear = I - FirstYear

swCheckFile.WriteLine(I.ToString + "," + RecordCount(IndexYear).ToString + "," + LSTonnes(IndexYear).ToString("F2") + "," + SSFTonnes(IndexYear).ToString("F2") + "," + IUUTonnes(IndexYear).ToString("F2") + "," + DiscardTonnes(IndexYear).ToString("F2"))

swCheckFile.Flush()

swGrandTotalFile.WriteLine(I.ToString + "," + RecordCount(IndexYear).ToString + "," + LSTonnes(IndexYear).ToString("F2") + "," + SSFTonnes(IndexYear).ToString("F2") + "," + IUUTonnes(IndexYear).ToString("F2") + "," + DiscardTonnes(IndexYear).ToString("F2"))

swGrandTotalFile.Flush()

Next

swCheckFile.Close()

SkiptoJ:

If CreatingSSFTotals Then

Dim swSSFTotals As New IO.StreamWriter(SSFCellCRFile)

swSSFTotals.WriteLine("IYear,Cell,CR")

For I = FirstYr(J) To LastYr(J)

IndexYear = I - FirstYear

For Cell = 1 To MaxCell

If SSFCellCR(Cell, IndexYear) > 0 Then

swSSFTotals.WriteLine(Str(I) + "," + Str(Cell) + "," + Str(SSFCellCR(Cell, IndexYear)))

swSSFTotals.Flush()

End If

Next

Next

swSSFTotals.Close()

End If

Next J

swGrandTotalFile.Close()

frm.ToolStripStatusLabel1.Text = "Finished..."

Application.DoEvents()

Windows.Forms.Cursor.Current = Windows.Forms.Cursors.Default

End Function
